# Hippocampal Ripple Diversity organises Neuronal Reactivation Dynamics in the Offline Brain

**DOI:** 10.1016/j.neuron.2025.09.012

**Published:** 2025-10-02

**Authors:** Manfredi Castelli, Vítor Lopes-dos-Santos, Giuseppe P. Gava, Renaud Lambiotte, David Dupret

**Affiliations:** 1 Medical Research Council Brain Network Dynamics Unit, Nuffield Department of Clinical Neurosciences, University of Oxford, UK; 2 Mathematical Institute, University of Oxford, UK

## Abstract

Hippocampal ripples are highly synchronized neuronal population patterns reactivating past waking experiences in the offline brain. Whether the level, structure, and content of ripple-nested activity are consistent across consecutive events or are tuned in each event remains unclear. By profiling individual ripples using laminar currents in the mouse hippocampus during sleep/rest, we identified Rad^sink^ and LM^sink^ ripples featuring current sinks in *stratum radiatum* versus *stratum lacunosum-moleculare*, respectively. These two ripple profiles recruit neurons differently. Rad^sink^ ripples integrate recent motifs of waking coactivity, combining superficial and deep CA1 principal cells into denser, higher-dimensional patterns that undergo hour-long stable reactivation. In contrast, LM^sink^ ripples contain core motifs of prior coactivity, engaging deep cells in sparser, lower-dimensional patterns that undergo a reactivation drift to gradually update their pre-existing content for recent wakefulness. We propose that ripple-by-ripple diversity supports parallel reactivation channels for integrating recent wakefulness while updating prior representations.

## Introduction

The brain has a remarkable ability to retain prior knowledge while continuously integrating new information. How neuronal populations coordinate these operations to enable seamless updating of brain networks remains a fundamental biological question. The offline states of sleep and rest could support this process by providing windows for the reorganization and stabilization of neural activity patterns ^1–3^. Hippocampal ripples have emerged as critical network events for facilitating offline processing of neuronal population activity ^4,5^.

Hippocampal ripples are transient network events characterized by high-frequency oscillations (100–250 Hz) in the local field potentials (LFPs) of the CA1 pyramidal cell layer (*stratum pyramidale*) ^6–8^. These events, prevalent during sleep and rest, are paired with a prominent negative deflection in the LFPs of the *stratum radiatum* – the sharp wave – reflecting CA3-to-CA1 inputs ^4,9,10^. The sharp-wave/ripple (SWR) complex has a well-defined laminar current source density (CSD) profile featuring a sink in the CA1 *stratum radiatum* ^4,6,10,11^. Ripples represent the most synchronous pattern of neuronal population activity in the mammalian brain, providing discrete windows during which hippocampal principal cells discharge action potentials collectively. This synchrony facilitates the “reactivation” of coactivity patterns from waking experiences for further offline processing ^12–21^.

The relevance of ripples has been demonstrated in studies showing that their suppression destabilizes hippocampal activity patterns and impairs memory ^8,22–27^, while promoting ripples and their associated spiking activity improves memory retention ^28,29^. Beyond their canonical role in memory, ripple-nested spiking is also linked to planning, inference, and even metabolism ^5,14,15,30–32^. Moreover, ripples provide a framework for brain-wide interactions, with both intra- and extra-hippocampal inputs shaping their electrophysiological features ^33,34^. Yet, ripples have typically been treated as a homogenous network pattern, raising the question of how they support diverse functions. Recent evidence suggests that hippocampal ripples are not uniform but exhibit significant variability in their electrophysiological expression, forming a continuum of features in the LFP waveform space ^35,36^. This variability may reflect differences in how inputs are organised along the somato-dendritic axis of CA1 principal cells ^37,38^. However, this diversity remains underexplored, as traditional analyses rely on averaging spectral characteristics, potentially masking important differences in these events ^34–36^. Identifying the ripple-by-ripple variability in activity levels, structural organization, and coactivity content is essential for understanding how these events support hippocampal computations.

To investigate hippocampal ripple diversity, we explored the CSD underlying each individual ripple across the CA1 layers. This revealed two laminar profiles that we refer to as Rad^sink^ versus LM^sink^ ripples, for *stratum pyramidale* events paired with a dominant current sink in *stratum radiatum* versus those with a dominant current sink in *stratum lacunosum-moleculare*. These profiles engage CA1 and CA3 neurons differently, hosting distinct millisecond-timescale population coactivity motifs. LM^sink^ ripples feature small, stronger coactivity motifs primarily involving deep-sublayer CA1 pyramidal cells. Following waking experience, Rad^sink^ ripples append recently coactive cells, predominantly from the superficial CA1 pyramidal sublayer, to these core motifs, yielding denser, higher-dimensional population patterns that are consistently reactivated over hour-long sleep/rest. Meanwhile, the pre-existing coactivity backbone nested in LM^sink^ ripples, formed by sparser, lower-dimensional patterns, gradually disengages throughout sleep/rest, slowly drifting to reflect recent wakefulness. Collectively, these findings reveal two ripple profiles with distinct population activity levels, structural organization, and neuronal content that cooperatively balance the integration of recent experiences with the updating of neuronal priors in the offline hippocampus.

## Results

### Profiling individual hippocampal ripples using their laminar currents

**Figure 1:**
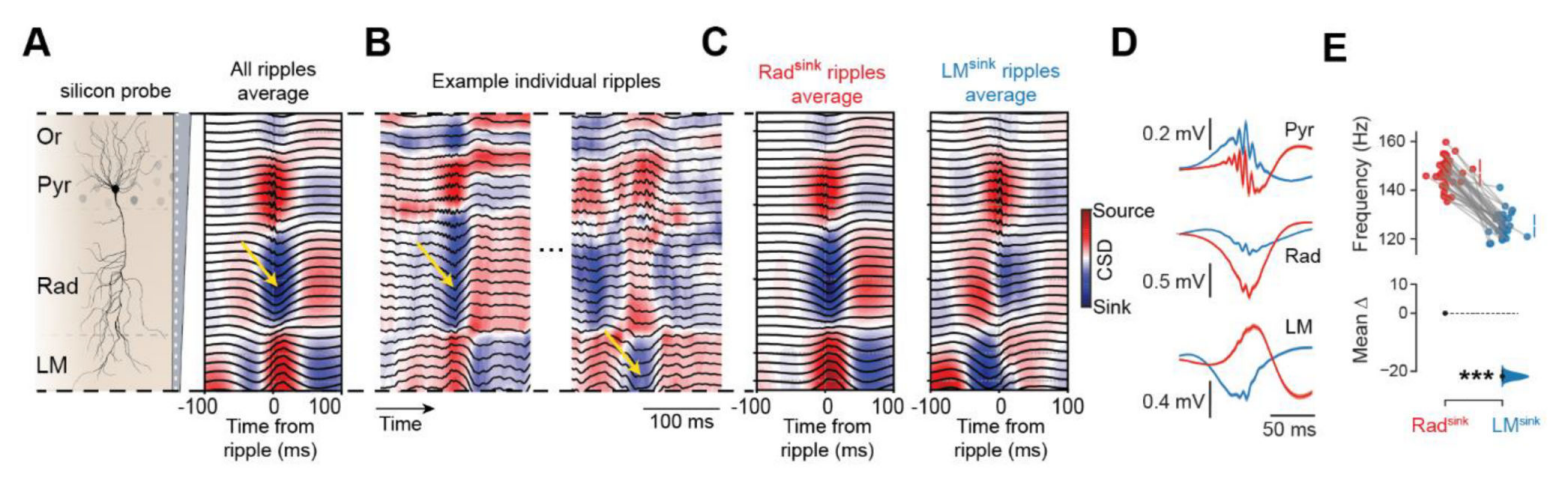
Laminar current profiles reveal two types of hippocampal CA1 ripples. **(A)** Average CSD (colour-coded map) and LFP waveform (black traces) for ripples recorded in *stratum pyramidale* using a silicon probe spanning CA1 layers (Or, *oriens*; Pyr, *pyramidale*; Rad, *radiatum*; LM, *lacunosum-moleculare*). **(B)** Instantaneous CSD and LFP waveform for two example ripples from one sleep session. **(C)** Average CSD and LFP waveform for ripples with a dominant current sink either in *radiatum* (Rad^sink^ ripples) or *lacunosum-moleculare* (LM^sink^ ripples). LM^sink^ ripples featured a double-sink profile: a first sink in *lacunosum-moleculare* preceding the ripple peak by an average of 17.3 ms (95% CI: 20.7–12.3 ms), and a second sink in *stratum radiatum* that occurred on average 12.4 ms after the ripple peak (95% CI: 8.8–16.2 ms). For this ripple type, the *lacunosum-moleculare* sink was significantly stronger than the *radiatum* sink (p = 0.018; paired bootstrap test; n = 38 sleep/rest sessions from five mice). **(D)** Average LFPs waveform for Rad^sink^ (red) and LM^sink^ (blue) ripples recorded in the pyramidal, radiatum, and lacunosum-moleculare layers. To visualise differences in ripple frequencies, LFP waveforms were referenced to the highest ripple-peak. *Shaded area* around each trace, 95% CIs. **(E)** Estimation plot showing the effect size for the difference in average ripple frequency between Rad^sink^ and LM^sink^ ripples. *Top panel*, raw data distributions (each dot represents a sleep session). *Bottom panel*, mean difference (*black dot*, mean; *black ticks*, 95% confidence intervals; *coloured area*, bootstrapped error distribution). ***p<0.001. (A and B) Yellow arrows show current sinks. (D and E) data shown are from n = 38 sleep/rest sessions from five mice.

We began investigating ripple diversity by simultaneously recording LFPs across CA1 layers using silicon probes (Table S1). This setup enabled ripple detection in the LFPs of *stratum pyramidale* and CSD analysis of laminar currents [Figures 1A-C and S1A; 52.2 total hours of sleep/rest from 5 mice; 38 sleep/rest sessions; mean duration (IQR): 82.4 (59.5 – 105.9) minutes per sleep/rest session]. In line with existing knowledge ^4^, the average ripple profile displayed a current sink in *stratum radiatum* and a current source in *stratum lacunosum-moleculare* (Figure 1A). However, individual ripples showed considerable variability in their CSD profiles (Figures 1B and S1B,C). Most individual ripples featured a dominant current sink in *stratum radiatum* [mean proportion (95% CI): 0.81 (0.76–0.87); Figures 1B and S1B,D,E], consistent with the grand average (Figure 1A). In addition to these canonical events, a subset of ripples exhibited a distinct CSD profile with a dominant current sink in *stratum lacunosum-moleculare* around the ripple peak [mean proportion (95% CI): 0.16 (0.12–0.20); Figures 1A,B and S1C-E]. A small fraction of ripples showed a dominant sink in *stratum oriens* [mean proportion (95% CI): 0.02 (0.01–0.03); Figures S1D-E]. The sink in *stratum oriens* was strongly correlated with the sink in *stratum radiatum* (Figure S1F,G). The distribution of ripples across these different current profiles was stable between pre- and post-exploration sleep/rest sessions (Figure S1E). During exploration, ripple events were too sparse to evaluate the ratio of dominant sinks.

We studied ripple variability using each event’s CSD signature, defined as the CSD signal within a 50-ms window centred on the ripple power peak. This allowed representing each ripple as a curve characterizing its laminar current profile along the CA1 somato-dendritic axis (Figure S1B,C). Principal component analysis of these CSD signatures unveiled two distinct laminar profiles forming a continuum (Figures 1C and S2A-E). One end of this continuum featured ripples characterized by a dominant sink in *stratum radiatum* (Rad), hereafter referred to as “Rad^sink^ ripples” (Figures 1C and S2D,E). Toward the other end, ripples exhibited progressively stronger current sinks in *stratum lacunosum-moleculare* (Figures 1C and S2D,E). Ripples at that end also exhibited a second, weaker sink in the *stratum radiatum* that emerged after the dominant *lacunosum-moleculare* sink; we refer to these as “LM^sink^ ripples” (Figure 1C). These profiles were consistently observed regardless of how LFP and the CSD signals were referenced to ripple events (Figure S2F-I). Ripples between the Rad^sink^ and LM^sink^ profiles are referred to as “intermediate ripples.”

In terms of LFP waveforms, Rad^sink^ ripples exhibited the well-described negative sharp-wave in the *stratum radiatum*, while LM^sink^ ripples showed a positive deflection in the *stratum pyramidale* accompanied by a negative deflection in the *stratum lacunosum-moleculare* (Figure 1D). Compared to Rad^sink^, LM^sink^ ripples exhibited lower frequency [Figures 1E and S2J-L; mean frequency (95% CI): Rad^sink^, 147.1 (145.3 – 148.8) Hz; LM^sink^, 125.4 (124.0 – 126.9) Hz; p < 10^-5^; paired bootstrap test]. This frequency difference persisted across sleep and pre-REM epochs, suggesting it does not reflect state transitions (Figure S2M,N). Rad^sink^ ripples showed higher power (Figure S2L,O) and longer duration (Figure S2P). The occurrence rates of Rad^sink^ and LM^sink^ ripples remained stable throughout NREM sleep (Figure S2Q,R). Further, ripples of the same profile tended to occur in bursts, while transitions between profiles occurred less frequently than expected by chance (Figure S2S,T).

### Cortical state bias in hippocampal ripple laminar profiles

**Figure 2:**
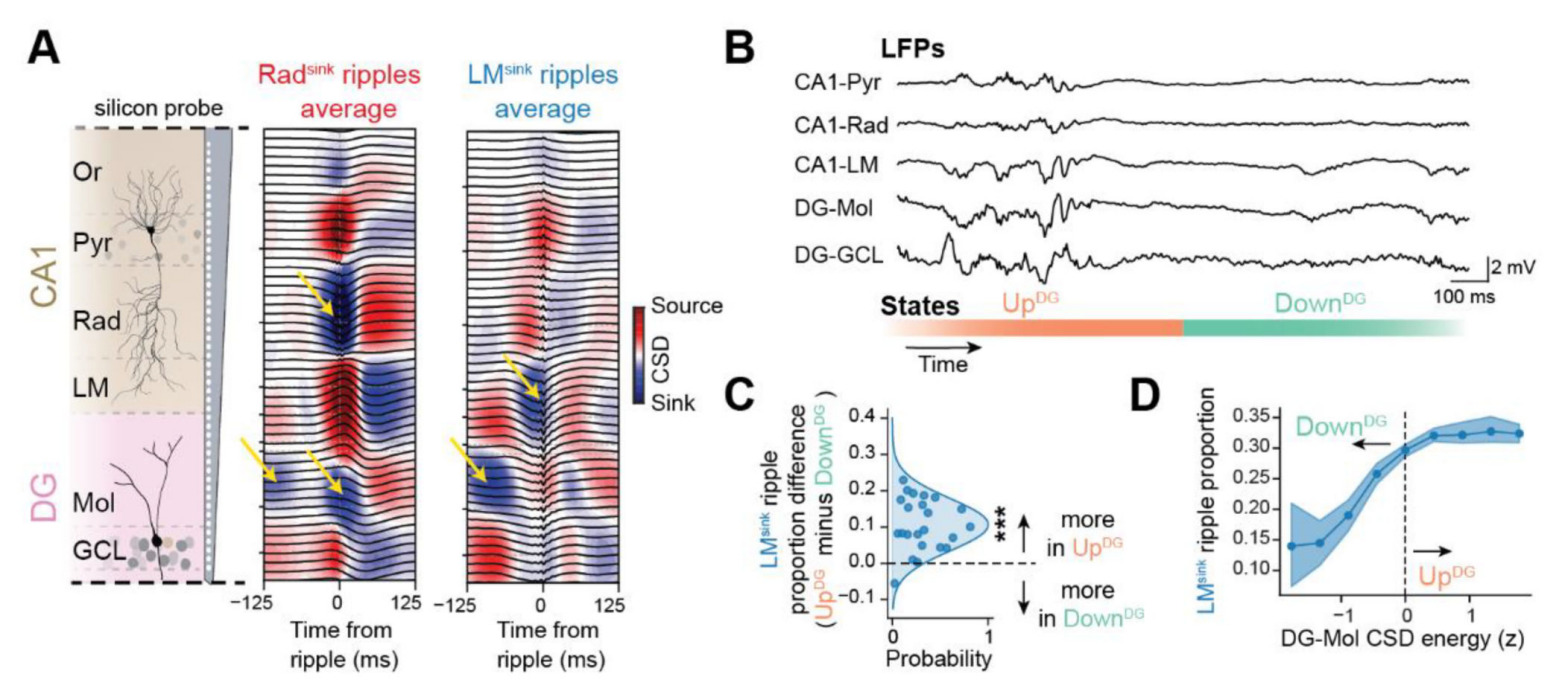
Up^DG^ and Down^DG^ states bias the laminar profile of ripple currents. **(A)** Average CSDs and LFP waveforms for Rad^sink^ and LM^sink^ ripples recorded using a silicon probe spanning layers along the CA1-to-DG axis (Or, *oriens*; Pyr, *pyramidale*; Rad, *radiatum*; LM, *lacunosum-moleculare*; Mol, *moleculare*; GCL, *granule cell layer*). Yellow arrows show current sinks. **(B)** Example raw LFPs simultaneously recorded from the CA1 stratum pyramidale, radiatum, and lacunosum-moleculare; and the DG stratum moleculare and granule cell layer during a detected Up^DG^ to Down^DG^ transition. **(C)** Difference in the proportion of ripples classified as LM^sink^ between Down^DG^ and Up^DG^ states. Each dot represents one sleep session. A significantly higher proportion of LM^sink^ ripples occurred during Up^DG^ states compared to Down^DG^ (mean difference > 0; p < 10^-5^; paired bootstrap test). **(D)** Proportion of ripples classified as LM^sink^ as a function of CSD energy in the DG molecular layer. Positive x-values correspond to Up^DG^ states; negative x-values reflect Down^DG^ states. *Shaded area*, 95% CI. *** p <0.001. (C and D) data shown are from n = 23 sleep/rest sessions from five mice.

To assess how Rad^sink^ and LM^sink^ ripples relate to broader sleep-related network dynamics, we analyzed their occurrence across cortical Up and Down states. These states were inferred from the relationship between entorhinal cortex (EC) activation and CSD signals in the dentate gyrus (DG) molecular layers ^11,39,40^ (Figures 2A,B and S3A). Both ripple types increased in incidence during Up^DG^ compared to Down^DG^ states (Figure S3B). However, the proportion of ripples classified as LM^sink^ significantly increased in Up^DG^ states (Figure 2C,D). Further, Rad^sink^ ripples peaked in occurrence shortly after the onset of Up^DG^ states, whereas LM^sink^ ripples were more evenly distributed throughout each state (Figure S3B).

These findings show that ripple occurrence generally increases during cortical Up states, consistent with previous reports ^41,42^. Additionally, the CSD profile associated with cortical Up states shows a relative increase of ripples with a lacunosum-moleculare sink, aligning with EC-to-CA1 projections targeting this layer ^43,44^. These observations suggest that offline EC activation not only increases the occurrence of hippocampal ripples but also biases their laminar current profile.

### Inferring ripple CSD laminar profiles from CA1 stratum pyramidale LFPs

**Figure 3:**
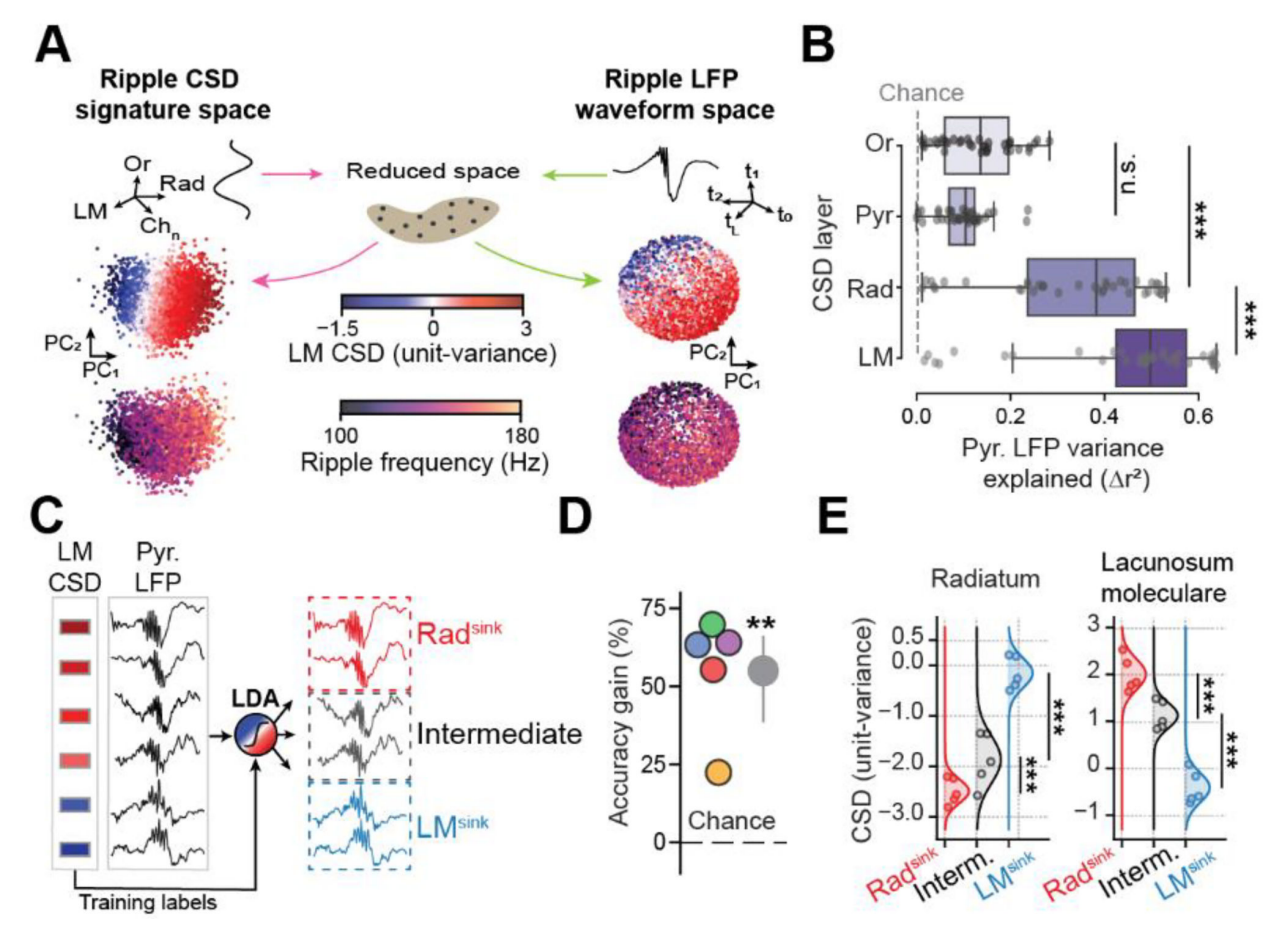
Latent CSD information embedded within CA1 stratum pyramidale LFPs. **(A)** Ripple-triggered CSDs and pyramidal layer LFP waveforms define a continuum capturing laminar and spectral ripple features. Top: schematic illustration of dimensionality reduction applied to ripple-triggered CSD profiles across CA1 channels (left; each channel as one dimension) and to pyramidal layer LFP ripple waveforms (right; each time point as one dimension). Middle and bottom: example projections from a single sleep session onto the first two principal components (PCs) of each space. Each dot represents one ripple, colour-coded by CSD in stratum lacunosum-moleculare (middle) or ripple frequency (bottom). Both reduced spaces preserve information about laminar CSD and spectral content. **(B)** Variance explained in the ripple pyramidal layer LFP by the concurrent CSD in each CA1 layer. Each dot represents a sleep session. The lacunosum-moleculare CSD explains most of the variance (p = 1.2x10^-24^, one-way ANOVA; Rad versus LM: p = 5.7x10^-4^, Tukey post-hoc; n = 38 sleep/rest sessions). **(C)** Schematic of linear discriminant analysis (LDA) predicting the CSD in *lacunosum-moleculare* from the ripple LFP signals in *stratum pyramidale*. Each ripple LFP trace and its corresponding CSD signal used in the LDA classifier to distinguish Rad^sink^, intermediate, and LM^sink^ ripples. **(D)** LDA accuracy. Each dot represents one mouse left outside the training set (leave-one-out approach). *Dashed line*, chance level; *gray dot*, mean; *vertical ticks*, 95% CI. **(E)** Radiatum and lacunosum-moleculare CSD values in LDA-classified Rad^sink^, intermediate (interm.), and LM^sink^ ripples. For each leave-one-out model, we applied the trained model to the mouse left-out of training and computed the mean CSD (unit-variance) in each predicted ripple using a window centred at time of the sink. Each dot represents one mouse left-out of training (n = 5 mice). LDA models significantly discriminated ripples with a dominant radiatum current sink, those with an intermediate CSD profile, and those with a dominant lacunosum-moleculare sink (radiatum CSD, p = 1.8x10^-6^; lacunosum-moleculare CSD, p = 4.2x10^-7^; one-way ANOVA with Tukey post-hoc; n = 5 mice) *p < 0.05, **p < 0.01, ***p<0.001.

Given the differences between Rad^sink^ and LM^sink^ ripple LFP waveforms (Figure 1D), we asked whether the laminar current profile underlying a given ripple could be inferred from CA1 stratum pyramidale LFPs. Consistent with recent work ^35^, ripple events did not form discrete clusters based on either their laminar current profiles or their pyramidal layer LFP waveforms (Figure 3A). We tested whether pyramidal-layer LFPs predicted ripple-related CSD signals across the different CA1 strata (i.e., oriens, pyramidale, radiatum, and lacunosum-moleculare). Using linear regressions, we found that CSDs in all layers were predicted above chance and throughout sleep (Figures 3B and S3C). The CSD from stratum lacunosum-moleculare was the most strongly explained by pyramidal layer LFPs (Figure 3B). Similar results were obtained using the Structure Index ^45^ (Figure S3D). Accordingly, we trained a linear discriminant analysis (LDA) model to classify individual ripple events using their pyramidal LFP waveforms, with class labels (Rad^sink^, intermediate, and LM^sink^ ripples) defined by the corresponding LM CSD (Figures 3C and S3E-I). To evaluate generalizability, the model was iteratively trained on data from a set of mice and tested on a different mouse (i.e., a leave-one-mouse-out cross-validation approach). The classifier reliably distinguished ripple types based solely on the pyramidal LFP waveforms [Figure 3D,E; mean accuracy gains over shuffle controls (95% CI): 55.0% (38.5–66.2); p = 0.0014, one-tailed t-test; model cross-validated across five mice]. Performance was unaffected by the recording depth within the CA1 pyramidal layer (Figure S3J).

Overall, these results demonstrate that hippocampal ripples vary in their laminar current profiles, forming a continuum that includes events with a prominent lacunosum-moleculare sink, which was not evident in the grand average profile. Moreover, the pyramidal layer LFP waveform allows distinguishing Rad^sink^ versus LM^sink^ ripples. Having established the classification procedure using leave-one-mouse-out cross-validation (Figure 3C-E), we trained a final model on the full silicon probe dataset (all mice) for application to the tetrode-recorded datasets in subsequent analyses.

### Rad^sink^ versus LM^sink^ ripples exhibit distinct population activity patterns

**Figure 4:**
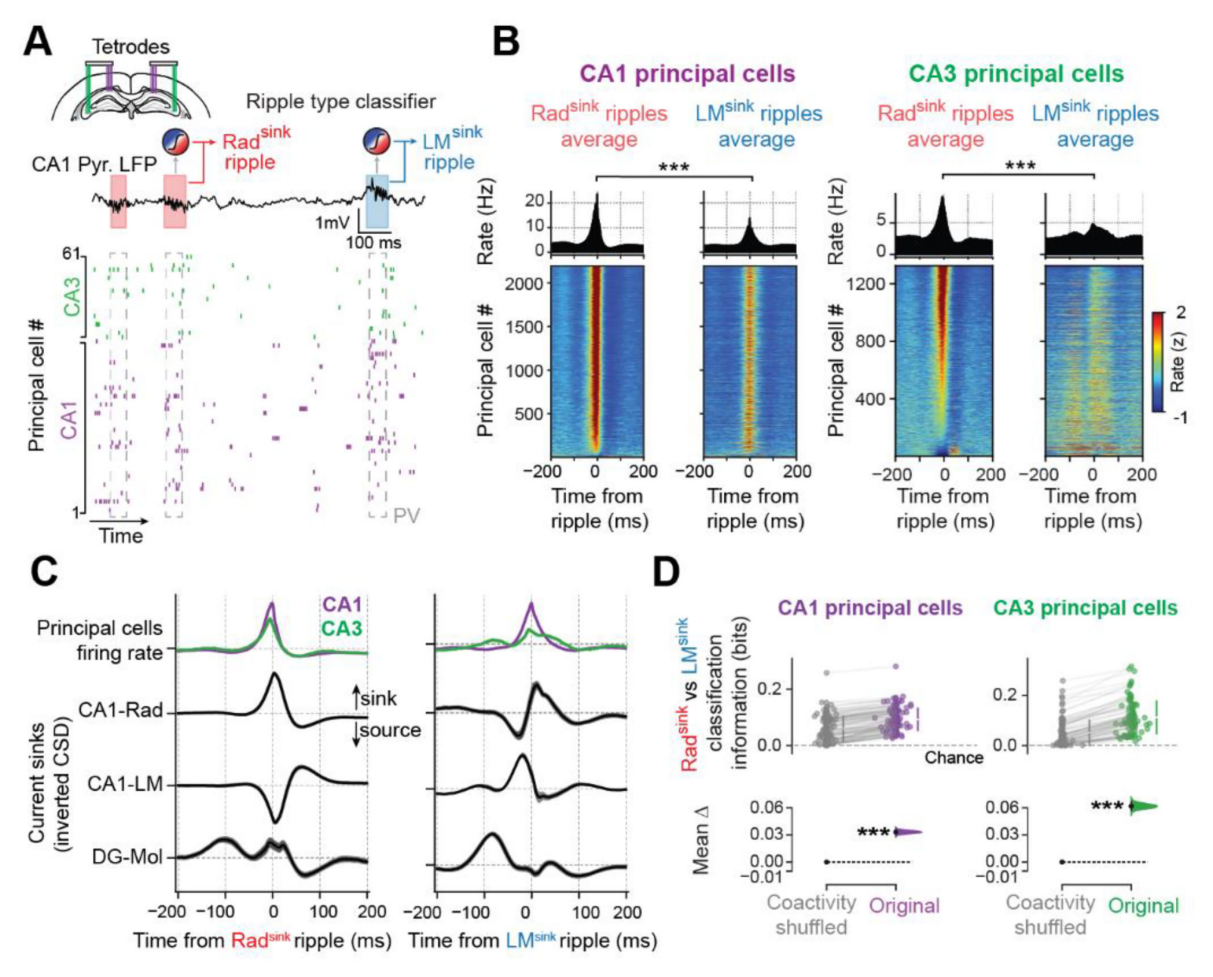
Principal cell firing response to Rad^sink^ versus LM^sink^ ripples. **(A)** Example dual-site 14-tetrode ensemble recording of CA1 and CA3 principal cells. *Top trace*, raw LFP signal from CA1 pyramidal layer. *Bottom raster plot*, (colour-coded) CA1 and CA3 principal cell spike trains (one cell per row; a 1-s sample recording for clarity). Population activity vector (PV) of individual ripple extracted using a 50-ms window centred at the ripple envelope peak. Tetrode-recorded Rad^sink^ and LM^sink^ ripples distinguished using the silicon probe-validated LDA (Figure 3C-E). **(B)** Triggered average firing response of CA1 and CA3 principal cells to Rad^sink^ and LM^sink^ ripples. *Top panels*, overall population average responses. *Bottom panels*, firing response (z-scored) of individual cells, sorted by their firing rates in Rad^sink^ ripples. **(C)** Ripple-triggered average firing of CA1 and CA3 principal cells during Rad^sink^ ripples (left) and LM^sink^ ripples (right), alongside inverted CSD traces (from the silicon probe dataset) in CA1 stratum radiatum (Rad), lacunosum-moleculare (LM), and DG molecular (Mol) layer. *Shaded areas around each trace*, 95% CIs (Rad and LM, n = 38 sleep/rest sessions; DG-Mol n = 23 sleep/rest sessions). **(D)** Estimation plots showing the difference in classifier accuracy (measured as mutual information) in discriminating Rad^sink^ and LM^sink^ ripples using ripple-nested PVs from CA1 (left) or CA3 (right). Each distribution observed in the real data (Original) is compared to chance level (dashed lines) and a surrogate distribution (coactivity shuffled) that preserved individual cell firing rates and overall population statistics while shuffling coactivity patterns (gray distributions). Each dot represents a single sleep session. For both CA1 and CA3, preserving population coactivity improved accuracy over both controls (CA1, p < 10^-5^; CA3, p < 10^-5^; paired bootstrap tests; n = 129 sleep/rest sessions for CA1, and n = 89 sleep/rest sessions for CA3). *p < 0.05, **p < 0.01, ***p<0.001.

We next tested whether Rad^sink^ and LM^sink^ ripples differentially engage neuronal spiking, using multichannel tetrode recordings simultaneously from CA1 and CA3 pyramidal layers [Figure 4A; mean principal cells per recording day (IQR): 42.9 (27.0 – 62.8); 3,521 total principal cells; 280 total hours of sleep/rest from 13 mice; 244 sleep/rest sessions; mean duration (IQR): 68.8 (48.0 – 90.2) minutes per sleep/rest session]. CA3 cells were monitored as they are the main upstream trigger for CA1 SWRs ^4^. Using the CSD-validated model (Figure 3C-E), we classified tetrode-recorded CA1 ripples as Rad^sink^ or LM^sink^ events [Figure 4A; mean number (IQR) of classified ripples per sleep/rest session: Rad^sink^, 1,062 (677.8 – 1,417.0); LM^sink^, 866.8 (490.8 – 1,146.3)]. Both ripple frequency and LFP waveform of tetrode-recorded Rad^sink^ and LM^sink^ ripples (Figure S3K,L) matched those recorded with silicon probes (Figure 1D,E).

CA1 principal cell activity increased in both Rad^sink^ and LM^sink^ ripples (Figure 4B), with higher firing rates in Rad^sink^ ripples [Figure S4A; mean peak rate (95% CI): Rad^sink^, 21.88 (21.27 – 22.50) Hz; LM^sink^, 13.12 (12.70 – 13.55) Hz; p < 10^-5^, paired bootstrap test; n = 2,196 CA1 principal cells]. CA1 principal cells fired at an earlier phase in LM^sink^ ripples yet maintaining similar coupling strength (Figure S4B,C). In CA3, principal cells also fired more in Rad^sink^ ripples [Figures 4B and S4D; mean peak rate (95% CI): Rad^sink^, 8.82 (8.14 – 9.54) Hz; LM^sink^, 5.18 (4.80 – 5.61) Hz; p < 10^-5^, paired bootstrap test; n = 1,325 CA3 principal cells]. CA3 principal cell firing further exhibited a distinct temporal pattern in LM^sink^ ripples, with an additional transient firing increase ~100 milliseconds before the ripple power peak (Figure 4B). CA3 interneurons also showed distinct firing patterns across ripple types (Figure S4E). In CA3, but not CA1, the interneuron-to-principal cell firing ratio was higher in LM^sink^ ripples (Figure S4F).

To examine the timing relationship between principal cell firing and laminar currents, we aligned the spiking activity (from the tetrode dataset) and the CSD signals (from the silicon probe dataset) to the ripple envelope peak, separately for Rad^sink^ and LM^sink^ ripples. In Rad^sink^ ripples, CA1 and CA3 principal cells showed strong firing response aligned to the radiatum sink (Figure 4C), consistent with previous work on canonical CA1 ripples ^10^. During LM^sink^ ripples, CA1 firing ramped up following the dominant lacunosum-moleculare sink and peaked before the radiatum sink reached its maximum (Figure 4C). This second LM^sink^ ripple sink in radiatum coincided with the rise in CA3 principal cell firing near the ripple peak. The pre-LM^sink^ ripple firing increase of CA3 principal cells (~100 ms before LM^sink^ ripple peak) coincided with a sink in the DG molecular layer (Figure 4C). Interestingly, the pre-LM^sink^ ripple firing rate of principal cells in CA3 (but not CA1) was strongly anti-correlated with their activity during Rad^sink^ ripples [mean Pearson correlation (95%CI): -0.34 (-0.39 – -0.28); Figure S4G,H], suggesting that these two profiles engage different cell populations.

To further explore this, we trained classifiers to discriminate ripple types using their instantaneous population activity vectors from CA1 or CA3 principal cells. As controls, we used surrogate population vectors that preserved both individual neurons firing rates (i.e., number of spikes each cell discharged across ripples) and population firing rates (i.e., number of spikes the population discharged in each ripple) while shuffling the combination of neurons coactive in each ripple (i.e., instantaneous population coactivity patterns). These (coactivity shuffled) control models still outperformed chance, indicating that some ripple-type information derived from firing rates (Figure 4D), consistent with the cross-ripple firing difference (Figures 4B and S4A,D). The observed CA1 and CA3 population coactivity patterns distinguished Rad^sink^ and LM^sink^ ripples, surpassing control models based on individual cells firing and population rates alone (Figure 4D), suggesting that these two ripple types engage distinct, non-redundant motifs of coactive neurons. Ripple classification was further improved when incorporating the full temporal structure of spike trains around each ripple event (Figure S4I-K and Table S2), especially in CA3, consistent with its strong pre-LM^sink^ and Rad^sink^ firing anti-correlation (Figure S4G,H). Together, these results indicate that LM^sink^ and Rad^sink^ ripples are associated with different spatiotemporal firing dynamics in both CA1 and CA3.

### Rad^sink^ ripples integrate additional neurons into core LM^sink^ coactivity motifs to form composite patterns

**Figure 5:**
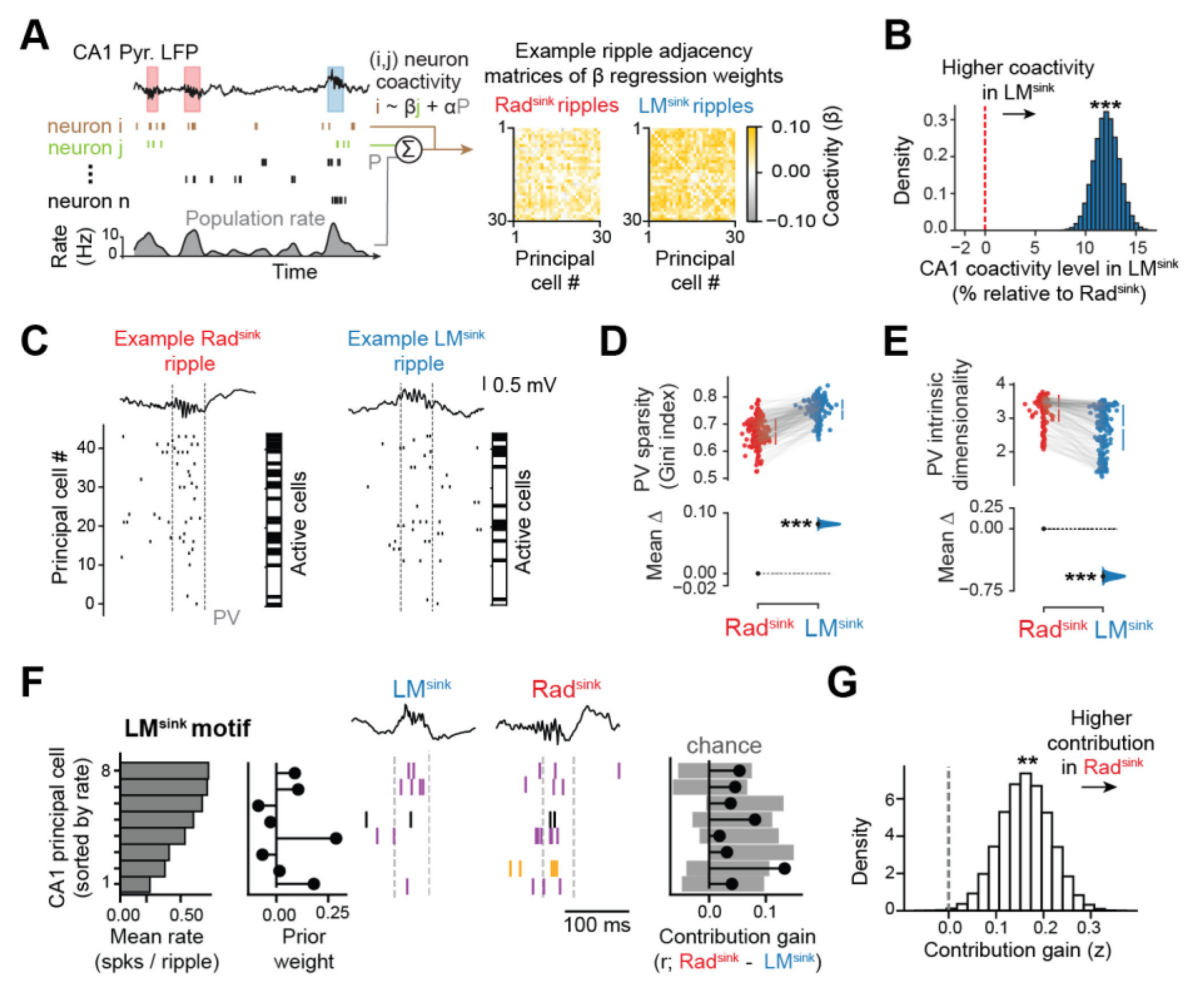
CA1 population coactivity level and structural organisation differ in Rad^sink^ versus LM^sink^ ripples. **(A)** Schematic outlining the measure of ripple-nested principal cell population-conditioned coactivity. *Left*, an example LFP trace from the CA1 pyramidal layer accompanies a raster plot of a subset of CA1 principal cells. Neuron *i* spiking is predicted by neuron *j* spiking while accounting for the population rate (*P*) using a linear regression model. *Right*, corresponding Rad^sink^ and LM^sink^ ripples adjacency matrices of beta coefficients. **(B)** Bootstrapped mean differences in population-conditioned coactivity of CA1 principal cells (LM^sink^ minus Rad^sink^; n = 1,580 cells). Difference expressed as a percentage relative to the mean coactivity in Rad^sink^ ripples. **(C)** Example raw LFP trace and raster plot of CA1 principal cells for one Rad^sink^ ripple and one LM^sink^ ripple, with the corresponding binarized population vectors (PVs) of active cells (black fields). **(D)** Estimation plot showing the difference in the sparsity (Gini index) of the population vectors nested in Rad^sink^ versus LM^sink^ ripples. Each point represents the mean sparsity of a single sleep session. **(E)** Estimation plots showing the difference in intrinsic dimensionality of population vectors in Rad^sink^ versus LM^sink^ ripples. Each point represents a single sleep session. **(F)** Example of a neuron joining an LM^sink^ core motif during Rad^sink^ ripples. Left: bar plot showing mean firing rates for a subset of CA1 principal cells (8 cells for clarity). Centre-left: coactivity motif detected during LM^sink^ ripples. Centre-right: raw LFP traces and raster plots for representative example individual LM^sink^ and Rad^sink^ ripples. Spikes from cells significantly contributing to the LM^sink^ motif shown in purple. Spikes from a non-contributing cell shown in black. Spikes from a cell not part of the LM^sink^ motif but with increased contribution during Rad^sink^ ripples shown in orange. Right: contribution gain to coactivity motif from LM^sink^ to Rad^sink^ ripples for each cell, shown alongside their null distribution (grey bars: 95% CI of surrogate contribution changes, controlling for excitability). Note that the ‘orange’ cell was not part of the LM^sink^ motif but significantly increased its contribution during Rad^sink^, indicating it was ‘aggregated’ onto the LM^sink^ motif. **(G)** Bootstrapped mean contribution gain of CA1 coactivity motifs (z-scored relative to surrogate distribution) from LM^sink^ to Rad^sink^ ripples. For each detected motif, the contribution gain of each cell quantified the increase in participation for that motif from LM^sink^ to Rad^sink^ ripples. For each motif, these chance-normalised contribution gains were averaged across all cells. Note that the mean of this gain is significantly positive, indicating that cells tended to be integrated into LM^sink^ motifs during Rad^sink^ ripples more than expected by their excitability (p = 2.5x10^-3^; bootstrap test; n = 231 motifs detected in 10 mice). *p < 0.05, **p < 0.01, ***p<0.001. (D and E) data shown are from n = 208 sleep/rest sessions from 13 mice.

We next characterised coactivity motifs in Rad^sink^ versus LM^sink^ ripples. For each ripple type, we measured the coactivity between cell pairs (*i, j*) using the regression coefficient from the prediction of neuron *i*’s spiking from neuron *j*, controlling for the remaining population (Figure 5A). CA1 principal cells showed higher coactivity in LM^sink^ ripples (Figures 5B and S5A,B; p < 10^-5^, paired bootstrap test), whereas CA3 principal cells showed higher coactivity in Rad^sink^ ripples (Figure S5A,C,D). Consistent with this, the graph-theoretical Structural Balance measure indicated that CA1 coactivity motifs were in LM^sink^ ripples were more stable, with no significant difference in CA3 (Figure S5E-H).

Compared to Rad^sink^, LM^sink^ ripples recruited a sparser CA1 principal cell population, as indicated by a higher Gini index (Figure 5C,D; p < 10^-5^, bootstrap test) and a lower proportion of active cells [Figure S5I; mean proportion of active CA1 cells (95% CI): Rad^sink^, 0.41 (0.40 – 0.42); LM^sink^, 0.28 (0.27 – 0.28); p < 10^-5^, paired bootstrap test]. Furthermore, LM^sink^ population vectors exhibited both lower intrinsic dimensionality ^46^ [Figure 5E; mean intrinsic dimensionality (95% CI): Rad^sink^, 3.29 (3.24 – 3.35); LM^sink^, 2.72 (2.63 – 2.81); p < 10^-5^, paired bootstrap test] and lower linear dimensionality (Figure S5J). This effect persisted across different cell sample sizes (Figure S5K) and was not explained by sparsity alone (Figure S5L). Thus, LM^sink^ ripples are characterized by sparse, low-dimensional population coactivity patterns, whereas Rad^sink^ ripples engage denser, higher-dimensional patterns.

LM^sink^ ripples have lower ripple power (Figure S2L,O), lower firing rates (Figures 4B and S4A), and sparser CA1 population recruitment (Figures 5D and S5I). However, LM^sink^ coactivity motifs did not simply reflect the engagement of higher-rate cells (likely those easiest to recruit with a weak drive) but instead reflected structured coactivity (i.e., the specific neurons that fire together) that was not solely driven by neurons’ excitability (Figure S6A-C).

We then tested whether the coactivity motifs in LM^sink^ ripples reoccur within the larger (denser) motifs observed in Rad^sink^ ripples. An asymmetrical Jaccard similarity coefficient indicated that LM^sink^ motifs are consistently re-expressed in Rad^sink^ ripples (Figure S6D-F), suggesting that Rad^sink^ events recruit the core motifs expressed in LM^sink^ ripples along with additional neurons. To explore this, we extracted individual LM^sink^ motifs from each sleep session using Independent Component Analysis ^47,48^ and quantified the contribution gain of each principal cell as the change in their cofiring with each LM^sink^ motif during Rad^sink^ ripples (Figure 5F). To control for firing rates and sparsity differences, we repeated the analysis using surrogate ripple activity that preserved both individual neuron rates per ripple type and total spike counts per event (Figure S6G). This confirmed that during Rad^sink^ ripples some individual neurons significantly increased their coactivity with LM^sink^ motifs (Figure 5G). Conversely, cell contribution gain from Rad^sink^ motifs in LM^sink^ ripples was significantly lower than expected from surrogates (Figure S6H). Moreover, the number of neurons significantly aggregated onto LM^sink^ motifs during Rad^sink^ ripples exceeded what was expected from the surrogate distributions (Figure S6I). These findings show that LM^sink^ ripples reflect sparse, low-dimensional ‘core’ motifs that reappear during Rad^sink^ ripples alongside additional neurons to form denser, higher-dimensional ‘composite’ patterns.

### Rad^sink^ and LM^sink^ ripples differentially recruit neurons across CA1 pyramidal sublayers

**Figure 6:**
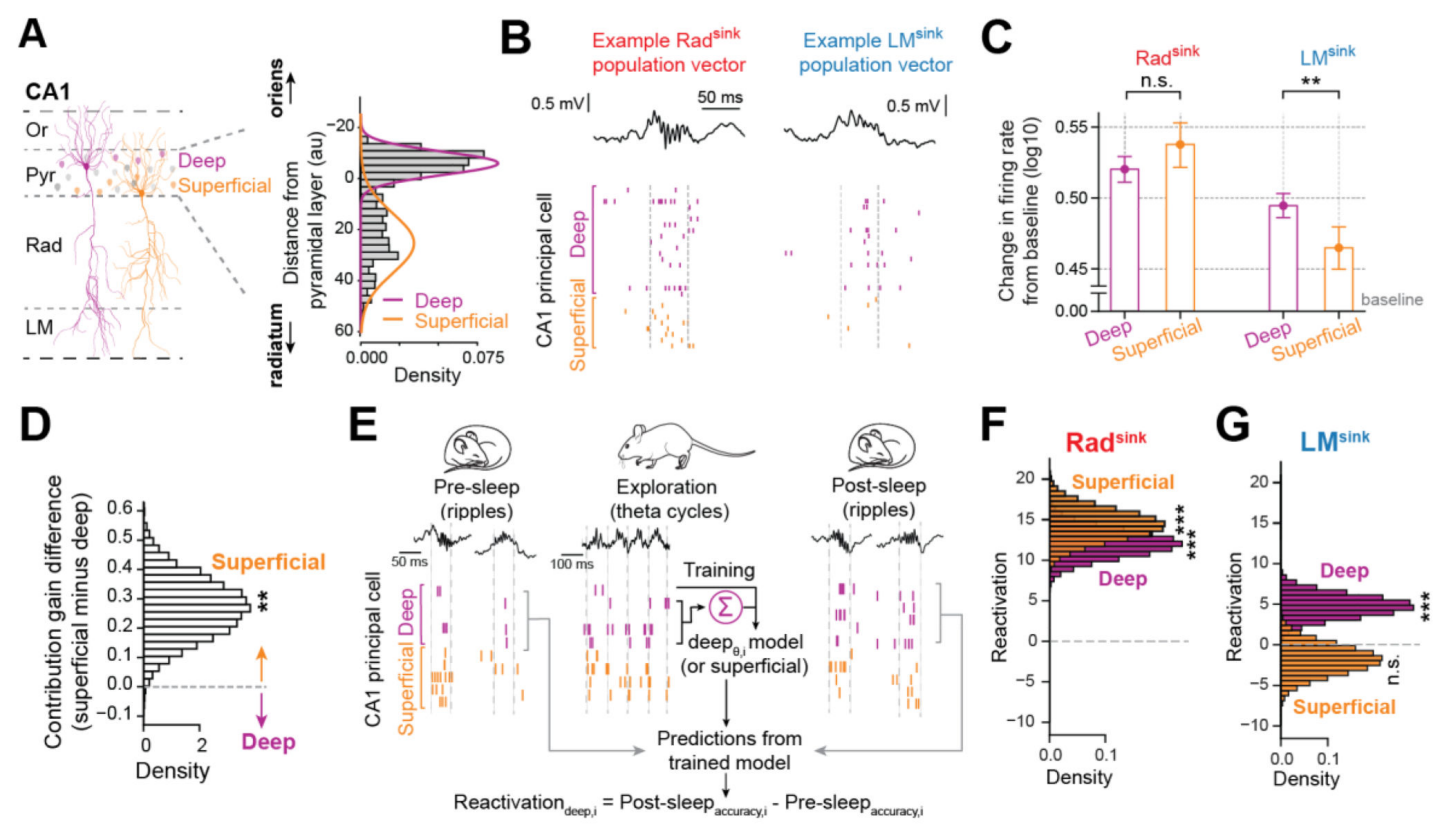
Firing response to Rad^sink^ and LM^sink^ ripples of principal cells in deep versus superficial CA1 pyramidal sublayers. **(A)** Distribution of CA1 principal cells recorded in the deep (purple) versus superficial (orange) pyramidal sublayers. **(B)** Example raw LFP traces and raster plots showing (colour-coded) spiking activity of deep and superficial cells in Rad^sink^ and LM^sink^ ripples. **(C)** Change in firing response of deep and superficial cells. In Rad^sink^ ripples, deep and superficial principal cells increase their firing rates similarly (relative to their pre-ripple baseline), whereas in LM^sink^ ripples, deep cells show a significantly greater firing increase compared to superficial cell (mean ± 95% CI; Rad^sink^, p = 0.10; LM^sink^, p = 3.2x10^-3^; bootstrap tests; n = 1,100 deep and 480 superficial cells). **(D)** Bootstrapped mean difference between CA1 sublayers (superficial minus deep) in contribution gain of principal cells to LM^sink^ motifs during Rad^sink^ ripples (measured as in Figure 5F,G). Superficial cells showed a significantly higher gain, indicating they are more likely to be integrated into the LM^sink^ motifs during Rad^sink^ ripples (p = 3.5x10^-3^; bootstrap test; n = 227 motifs with deep cells and n = 157 motifs with superficial). **(E-G)** Rad^sink^ ripples reactivate waking theta coactivity of both deep and superficial cells but LM^sink^ ripples selectively reactivate deep cells. Shown in (E) is a schematic of the method for computing offline reactivation. Using theta cycles in active exploration, a generalized linear model (GLM) was trained to predict the activity of each principal cell from the waking theta spiking activity of the other cells in the same sublayer. Each theta model was then applied to predict the response of its target cell during Rad^sink^ and LM^sink^ ripples of pre-exploration sleep versus post-exploration sleep. Reactivation was measured as the difference in model accuracy in post-exploration sleep relative to pre-exploration sleep. Shown in (F) is the bootstrapped mean difference in reactivation during Rad^sink^ ripples for deep and superficial cells. Both sublayers exhibit significant Rad^sink^ reactivation [all Ps < 10^-5^; 1-tailed bootstrap tests]. (G) as (F), but for LM^sink^ ripples. Note that only deep cells reactivate significantly during post-sleep LM^sink^ ripples [deep cells: p = 2.7x10^-4^; superficial cells: p = 0.84; 1-tailed bootstrap tests; n = 1,097 deep and 478 superficial cells]. *p < 0.05, **p < 0.01, ***p < 0.001. (A) show data from n = total 1,353 deep and 843 superficial cells from 13 mice.

Traditionally, hippocampal network function has been studied assuming CA1 principal cells as a homogeneous population. Recent research has revealed significant heterogeneity, segregating CA1 pyramidal cells into two subpopulations with distinct properties ^49–65^. For example, deep-sublayer CA1 pyramidal cells exhibit higher firing rates than those in the superficial sublayer during awake theta oscillations ^37,66^, whereas superficial-sublayer cells show higher firing rates during ripples ^37,51,67^ (see also Figure S6J,K). We assessed whether LM^sink^ and Rad^sink^ ripples differently engage deep and superficial cells (Figure 6A) ^37^. For each cell population, we calculated the change in firing rates from their pre-ripple baseline activity (using a 200 to 100 ms window before ripple power peak) to each event type. During Rad^sink^ ripples, superficial and deep cells increased their firing similarly (Figure 6B,C). However, during LM^sink^ ripples, deep cells showed higher firing increase than superficial cells (Figures 6B,C and S6L). Consistent with this, the cells aggregated onto LM^sink^-defined coactivity motifs during Rad^sink^ ripples (Figures 5F,G and S6I) were significantly biased toward the superficial sublayer (Figure 6D).

To examine offline reactivation during Rad^sink^ and LM^sink^ ripples, we trained generalized linear models to predict each principal cell’s activity during awake theta oscillations based on peer activity (Figure 6E). Reactivation was then assessed by applying each cell’s theta-coactivity model to predict its ripple-nested activity in post-exploration sleep/rest, controlling for pre-exploration sleep/rest. When applied across all CA1 principal cells (regardless of somatic location), both Rad^sink^ and LM^sink^ ripples showed significant reactivation of waking theta activity, with stronger levels in Rad^sink^ ripples (Figure S7A). When analyzing deep and superficial principal cells separately, both subpopulations reactivated their waking theta coactivity in Rad^sink^ ripples (Figure 6F), whereas only deep cells significantly reactivated in LM^sink^ ripples (Figure 6G). CA3 principal cells mirrored CA1 superficial cells, reactivating in Rad^sink^ but not in LM^sink^ ripples (Figure S7B).

### Temporal stability of Rad^sink^ recent patterns and gradual drift of LM^sink^ prior patterns

Although LM^sink^ ripples showed stronger coactivity (Figures 5B and S5B), theta-nested population patterns from recent experience were more strongly reactivated in Rad^sink^ ripples (Figure S7A). This was particularly observed in CA1 superficial cells (Figure 6F), which showed no detectable reactivation in LM^sink^ ripples (Figure 6G). This led us to hypothesize that LM^sink^ coactivity motifs are more biased toward pre-existing patterns, whereas Rad^sink^ coactivity is more influenced by recent wakefulness.

**Figure 7:**
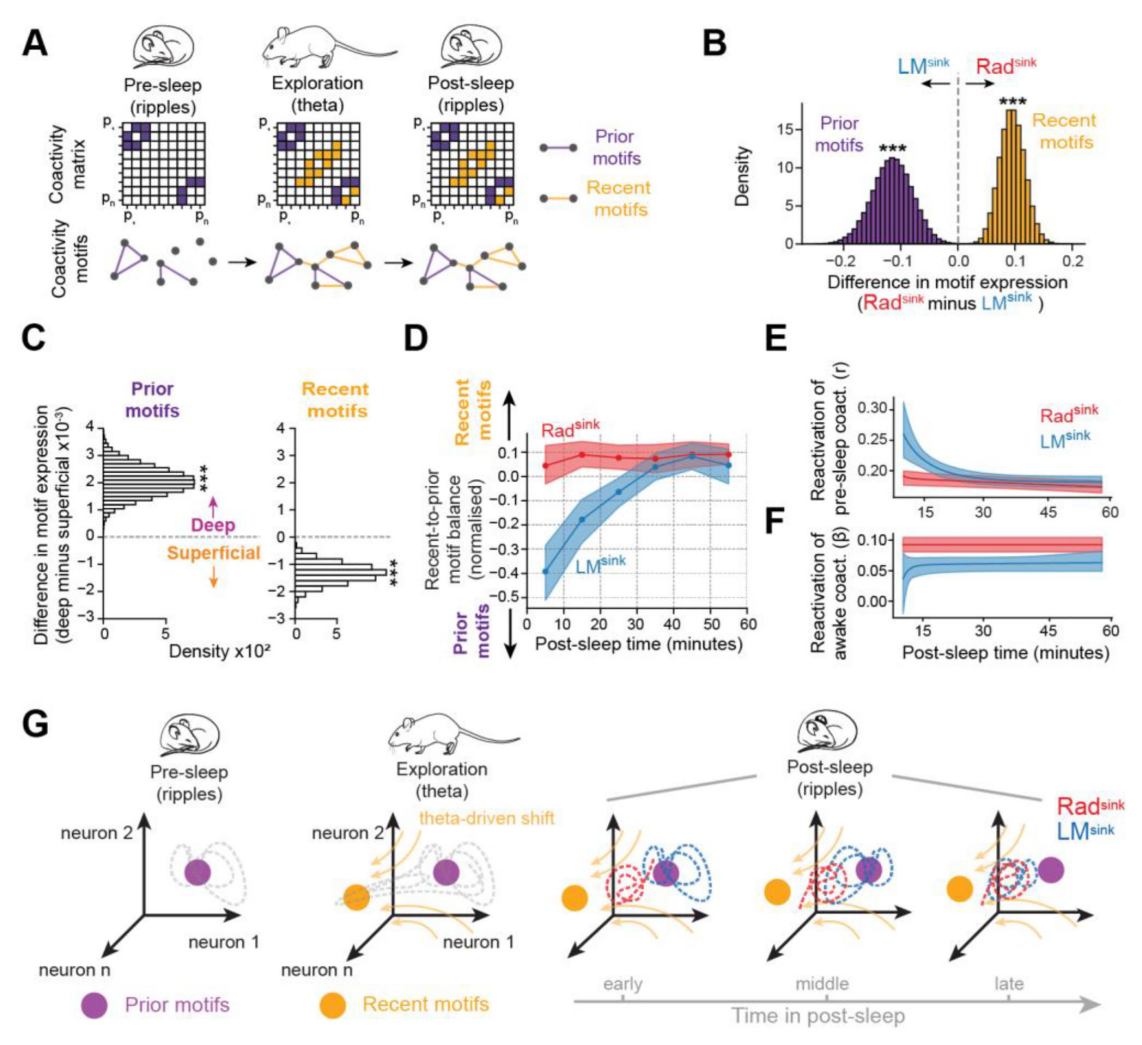
Prior versus recent coactivity motifs dynamics in Rad^sink^ and LM^sink^ ripples. **(A)** Schematic of the method identifying prior and recent coactivity motifs (see also Figure S7C-F). Coactivity matrices (top) and corresponding neuronal graph motifs (bottom) across pre-exploration sleep (pre-sleep ripples), exploration (theta cycles), and post-exploration sleep (post-sleep ripples). Purple squares denote cell pairs constituting prior coactivity motifs (i.e., already present in pre-sleep), while orange squares show coactivity relationships that selectively emerged during exploration (i.e., absent in pre-sleep). **(B)** Bootstrapped mean difference (Rad^sink^ minus LM^sink^) in motif expression during post-sleep for prior and recent motifs. Rad^sink^ ripples expressed recent coactivity motifs more strongly than LM^sink^ ripples (p < 10^-5^; two-sided bootstrap test), whereas LM^sink^ ripples more closely aligned with prior motifs (p = 6x10^-4^; two-sided bootstrap test). n = 140 pre- and post-sleep sessions pairs from 13 mice. **(C)** Bootstrapped mean difference in expression strength of prior (left) and recent (right) motifs between deep and superficial cells (relative to the mean of superficial cells). Deep cells were more aligned with prior motifs than superficial cells (p = 4x10^-5^; two-sided bootstrap test), whereas superficial cells expressed recent motifs more strongly (p = 8x10^-5^; two-sided bootstrap test). n = 43 pre- and post-sleep sessions pairs from 6 mice. **(D)** Time course of the recent-to-prior motif balance in Rad^sink^ and LM^sink^ ripples over the hour-long sleep/rest (normalized by subtracting the overall mean reactivation of that session). LM^sink^ showed an exponential increase [Bayesian Information Criterion (BIC): BIC_flat_ = - 1107.8; BIC_exp_= -1129.7], showing that LM^sink^ coactivity gradually became more biased towards recent motifs. Rad^sink^ ripples exhibited stable recent-to-prior motif balance throughout hour-long sleep/rest (BIC_flat_ = -1491.5; BIC_exp_= -1478.8). *Shaded areas*, 95% CI. **(E)** Reactivation time course of pre-exploration sleep coactivity, computed for Rad^sink^ and LM^sink^ ripples during post-exploration sleep. LM^sink^ ripples showed an exponential decrease (BIC_flat_ = -2854.6; BIC_exp_=-2855.4), suggesting gradual disengagement of prior coactivity motifs. Reactivation strength of pre-sleep coactivity was stable in Rad^sink^ ripples (BIC_flat_ = - 2987.5; BIC_exp_= -2976.8). *Shaded areas*, 95% CI (n = 10,000 exponential fit bootstraps using data from 116 pairs of pre- and post-sleep sessions from 13 mice). **(G)** Similar to (E) but showing reactivation time course of recent waking theta coactivity, computed for Rad^sink^ and LM^sink^ ripples throughout post-exploration sleep. Both LM^sink^ (BIC_flat_ = -2017.2; BIC_exp_= -2004.5) and Rad^sink^ (BIC_flat_ = -2255.6; BIC_exp_= -2248.5), reactivation values were stable and significantly above zero, suggesting that recent coactivity motif expression did not change over time_._ *Shaded areas*, 95% CI (n = 10,000 exponential fit bootstraps using data from 118 pairs of pre- and post-sleep sessions from 13 mice). **(H)** Schematic illustrating the drift of prior versus recent motifs in neural space across sleep and wake. Left: during pre-exploration sleep (ripples), CA1 neurons express coactivity motifs whose neural trajectories (dashed line) gravitates around a ‘backbone’ of prior motifs (purple circle). Centre: during wakefulness (theta cycles), experience-related motifs (orange circle) attract the neural trajectory (theta-driven shift). Right: in post-exploration sleep, Rad^sink^ ripples express recent motifs from the beginning. In contrast, LM^sink^ ripples initially align with prior motifs but then gradually drift toward recent motifs. *p < 0.05, **p < 0.01, ***p < 0.001.

To test this, we quantified how strongly post-exploration sleep ripples expressed coactivity motifs that were strengthened during preceding waking exploration relative to pre-exploration sleep (Figures 7A and S7C–F). This recent-to-prior motif balance showed that post-exploration Rad^sink^ ripple activity was more aligned with recent (waking theta) motifs than LM^sink^ activity, which remained more biased toward prior (pre-exploration sleep) motifs (Figure 7B). Similar results were obtained for CA3-CA1 neuron pairs (Figure S7G). Post-exploration sleep alignment with prior motifs in LM^sink^ ripples was stronger in deep CA1 principal cells, whereas alignment with recent motifs in Rad^sink^ ripples was stronger in superficial cells (Figure 7C).

We then analysed temporal dynamics of CA1 population patterns during extended sleep [mean duration (IQR): 86.25 (76.33 – 91.62) minutes per sleep; n = 118 pairs of pre- and post-exploration sleep sessions from 13 mice]. Although LM^sink^ ripple activity was more aligned with prior motifs when averaged across long (>60 min) post-exploration sleep periods, analysing it over time uncovered a progressive drift toward the recent motifs, reaching expression levels comparable to Rad^sink^ ripples within ~30 minutes [Figures 7D; time constant *τ* (95% CI), 13.12 (8.34 – 21.80) minutes]. In contrast, Rad^sink^ ripples showed no such gradual change (Figures 7D). The population drift observed in LM^sink^ ripples from prior to recent motifs over time was explained by ripple occurrence time and not by ripple occurrence frequency or changes in ripple population sparsity (Figure S7H-J).

These findings suggested that LM^sink^ ripple activity progressively transitioned across post-exploration sleep, drifting away from prior coactivity motifs to converge toward recent motifs (Figures 5D). However, it remained unclear whether this gradual shift in population motifs reflects a progressive disengagement from prior coactivity structure, an increasing expression of recent motifs, or a combination of both. To address this, we performed two complementary analyses on prior and recent motifs separately.

First, we examined whether the expression of prior coactivity motifs in LM^sink^ ripples declined over post-exploration sleep. To do so, we trained generalized linear models to predict each principal cell’s activity from its peers (as in Figure 6E) during pre-exploration sleep ripples and used their prediction accuracy when applied to post-exploration sleep Rad^sink^ or LM^sink^ ripples to quantify the reactivation strength of pre-sleep coactivity. Averaged over the entire post-exploration sleep, the reactivation strength of pre-sleep coactivity in LM^sink^ ripples was stronger than in Rad^sink^ ripples (Figure S7K). However, in line with the trend observed in the recent-to-prior motif balance analysis (Figures 7D), the reactivation of pre-exploration sleep coactivity declined exponentially in post-exploration sleep LM^sink^ ripples while it remained stable in Rad^sink^ ripples (Figure 7E). This confirmed a progressive disengagement of prior motifs in LM^sink^ ripples throughout post-exploration sleep reactivation.

Second, we examined whether the reactivation of recent coactivity motifs in post-exploration sleep LM^sink^ ripples increased over time. To test this, we measured the reactivation strength of wakefulness coactivity by computing the pairwise correlations of CA1 principal cell activity between exploration theta and post-exploration sleep ripples, regressing out the coactivity observed in pre-exploration sleep ripples. Both ripple types significantly reactivated recent waking theta coactivity, and this remained stable throughout post-exploration sleep (Figure 7F).

Thus, the population drift from prior to recent coactivity motifs in post-exploration sleep LM^sink^ ripples (Figure 7D) was largely explained by the gradual disengagement of prior (pre-exploration sleep) motifs over time (Figure 7E), increasing the ratio of recent-to-prior motif expression (Figure 7D). This prior motif disengagement was better accounted by the gradual shift in coactivity among deep CA1 principal cells (Figure S7L,M), drifting away from their initial stronger participation in prior motifs (Figure 7C). In sum, Rad^sink^ ripple coactivity showed stronger alignment with recent waking theta motifs from the beginning till the end of post-exploration sleep, whereas LM^sink^ ripple coactivity gradually drifted away from prior motifs toward recent motifs, eventually reaching expression levels comparable to Rad^sink^ ripples (Figure 7G).

## Discussion

**Figure 8:**
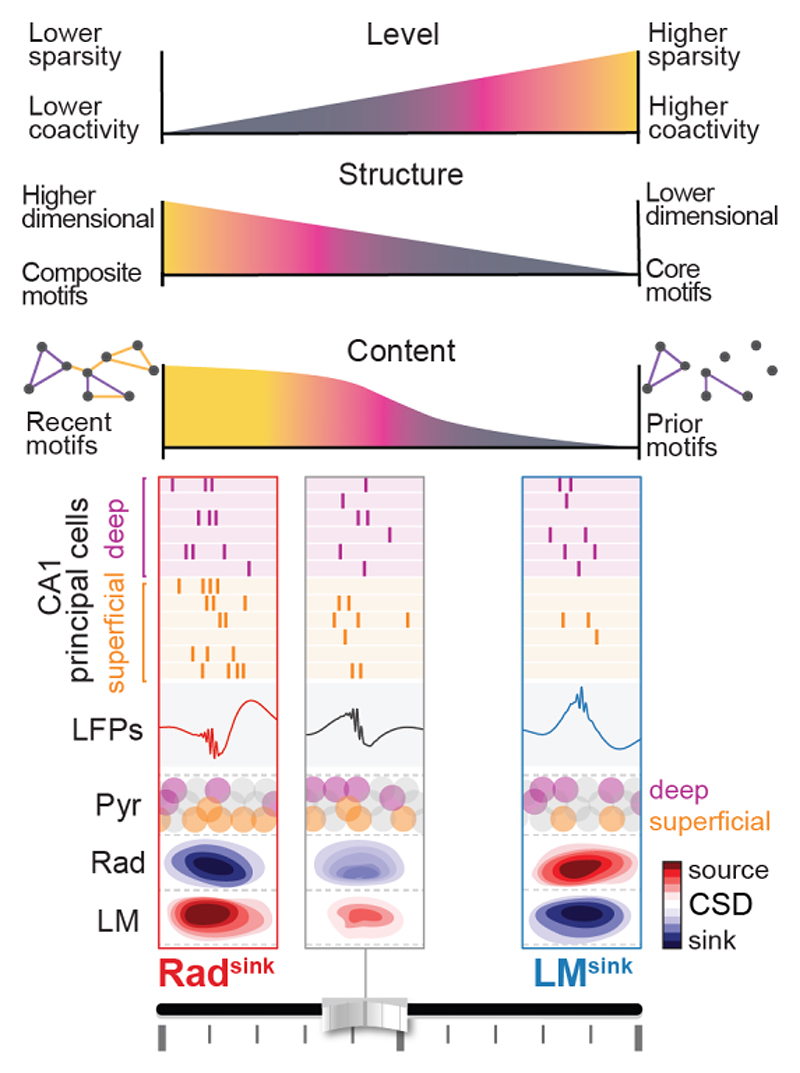
Summary schematic. Two ripple profiles, identified by their radially organised currents, exhibit distinct CA1 population activity level, structure, and content. Rad^sink^ ripples, with stronger *radiatum* current sinks, integrate recent motifs of waking coactivity, combining superficial with deep principal cells into composite and higher-dimensional population patterns that undergo stable reactivation over hour-long sleep/rest. In contrast, LM^sink^ ripples, with stronger *lacunosum-moleculare* current sinks, contain core motifs of pre-structured coactivity, engaging deep cells into lower-dimensional patterns that gradually drift from expressing prior to recent motifs throughout sleep. Dynamical tuning (depicted by the fader at the bottom) of population activity between these two ripple profiles could support parallel reactivation channels for integrating recent wakefulness while preserving prior representations.

In this study, we identified two ripple profiles that exhibit distinct laminar currents across the CA1 somato-dendritic axis and differentially recruit hippocampal neurons during offline periods of sleep/rest. The first profile, with a dominant current in stratum radiatum, combines superficial and deep CA1 principal cells into higher-dimensional composite patterns that undergo hour-long stable reactivation of recently expressed waking motifs of neuronal coactivity. The second ripple profile, with a dominant current in stratum lacunosum-moleculare, contains core motifs that primarily recruit deep cells in lower-dimensional patterns that undergo time-varying reactivation, gradually drifting over sleep from prior to recent coactivity spaces. Collectively, these findings reveal a diversity in hippocampal ripples that is linked to the offline organisation of neuronal reactivation. We propose that by tuning differences in the activity level, structural organisation, and neuronal content of population patterns (Figure 8), this ripple-by-ripple diversity supports two parallel processing channels for consolidating recent waking experience while updating prior memory representations in the offline hippocampus.

### Ripple-by-ripple variability reveals distinct laminar current profiles

We began our investigation by analysing the variability in CSD profiles of individual ripples. These profiles form a continuum rather than discrete clusters, aligning with recent work showing that ripple waveforms and features occupy a continuum of parameter-space ^35^. This might enable the dynamical modulation of the hippocampal population activity on a ripple-by-ripple basis using transient changes of currents in specific neural layers. The dominant contributors to this continuum are ripples associated with a stronger current sink in *stratum radiatum* (Rad^sink^ ripples), reflecting the canonical CSD profile of the grand average ripple. In addition to these are ripples that instead show a stronger current sink in *stratum lacunosum-moleculare* (LM^sink^ ripples), thus far hidden in the grand average. CA3 projections to CA1 *stratum radiatum* play a key role in sharp-wave/ripple generation ^4,68,69^, consistent with the observed large average input current in this layer (Figure 1A). Yet, both this study and previous work ^35^ reveal significant variability in current profiles associated with individual ripples. Using an unsupervised approach, we identified here a subset of ripples with a dominant current sink in *stratum lacunosum-moleculare* and slower ripple frequency. Interestingly, following the dominant sink in stratum lacunosum-moleculare, this subset of LM^sink^ ripples then exhibits a second sink in stratum radiatum following the ripple peak, suggesting a complex interplay of temporally distinct inputs shaping these events.

This is in line with recent work by Sebastian et al. ^35^, which reported a subset of ripples with a current sink in *stratum lacunosum-moleculare*, supporting the idea that cortical drive can influence ripple waveform shape. In line with our findings, a recent study also highlights that ripples lacking sharp waves (interpreted as events with stronger cortical influence) show reduced CSD energy in *stratum lacunosum-moleculare*, suggesting a decrease in the overall global current flow ^70^. Our results extend this observation by showing that ripples with weaker CA3 drive are associated with a dominant current sink in LM, supporting the presence of cortical input during ripples with no radiatum sharp waves. This LM^sink^ ripple profile likely implicates projections from the medial entorhinal cortex layer III (EC3) to CA1, suggesting a complex interplay of CA3 and EC3 inputs in CA1 ripple generation.

In line with this hypothesis, we found that the proportion of LM^sink^ events increased during Up^DG^ states, suggesting a greater contribution of EC-driven activity to this ripple profile. This also indicates that the overall diversity of ripple CSD profiles is biased and embedded within broader sleep-state dynamics. Consistent with these findings, removing CA3 inputs does not entirely prevent ripple generation; instead, slower-frequency ripples persist in the absence of CA3 ^69^, while lesions to the MEC are associated with changes in the proportion of high-to-low frequency ripples ^71^. Furthermore, recent findings indicate that EC inputs to CA1 can shape ripples characteristics ^60,71,72^. This transient tuning on an moment-by-moment basis across individual ripples is reminiscent of that previously observed across individual cycles of hippocampal theta oscillations during awake exploration ^57,73^. Similar to waking theta cycles, our findings show that the hippocampus dynamically tunes offline population activity on a ripple-by-ripple basis, in line with recent work suggesting that a temporal microstructure of sleep embeds the reactivation of co-existing hippocampal patterns ^27^.

### Rad^sink^ and LM^sink^ ripples differentially modulate hippocampal cells and circuits

We found that principal cells and interneurons in both CA1 and CA3 were recruited at varying levels across the two ripple profiles. Rad^sink^ ripples have higher firing rates and denser recruitment of neurons compared to LM^sink^ ripples. Furthermore, in CA3, beyond these quantitative differences in neuronal recruitment, we observed qualitatively different temporal firing responses in Rad^sink^ and LM^sink^ ripples. In particular, while Rad^sink^ ripples were associated with CA3 activity aligned to a current sink in stratum radiatum (Figure 4C), LM^sink^ ripples showed a distinct temporal pattern: the pre-LM^sink^ ripple increase in CA3 firing (~100 ms before ripple peak) coincided with a current sink in the DG molecular layer, followed by a later, sustained peak in CA3 activity that coincided with a radiatum sink toward the end of the ripple (Figure 4C). This delayed radiatum sink emerged only after both the lacunosum-moleculare current sink and CA1 principal cell firing had peaked (Figure 4C).

These findings further support the idea that these two ripple CSD profiles arise in the hippocampal circuit differently. The high recurrency in CA3 could enable strong excitatory currents converging to *stratum radiatum* during Rad^sink^ ripples, activating a larger number of neurons at higher firing rates and producing higher-frequency ripples. In contrast, during LM^sink^ ripples, CA3→CA1 inputs would be significantly weaker, either intrinsically or attenuated locally by interneurons within CA3 or CA1, promoting stronger differential current in *stratum lacunosum-moleculare*. These distal currents still recruit a population of neurons at lower firing rates than in Rad^sink^ ripples. This reduced recruitment may stem from weaker input currents from CA3 during LM^sink^ ripples or from the additional attenuation caused by the longer distance these currents must travel to generate somatic spikes in CA1, compared to the shorter path from *stratum radiatum*. Our findings establish that within the continuum of CSD profiles, CA1 and CA3 principal cells are modulated to varying degrees, reinforcing the dynamic nature of ripple-associated recruitment in the hippocampal circuitry.

The differences between Rad^sink^ and LM^sink^ ripples extend beyond modulation of activity levels to include distinct structural properties of the population patterns formed by principal cell coactivation in these events. Specifically, we show that different coactivity motifs are recruited across the two ripple profiles. CA1 principal cells contributing to the sparser LM^sink^ ripples formed lower-dimensional core motifs of high coactivity, which also reappear in higher-dimensional Rad^sink^ ripples alongside additional cells. Importantly, neuronal recruitment during LM^sink^ ripples reflects structured coactivity beyond what individual firing rate differences alone can explain (Figure S6A-C). These findings suggest that, despite potentially originating from parallel hippocampal circuits, CA1 principal cell population responses to Rad^sink^ and LM^sink^ ripples can influence each other.

### Distinct CA1 pyramidal sublayer dynamics during Rad^sink^ and LM^sink^ ripples

Recent studies have documented significant diversity among CA1 pyramidal cells, emphasizing that their somatic location in the deep versus superficial sublayers of the *stratum pyramidale* predicts distinct contributions to hippocampal network dynamics (e.g., ^37,49,51,57,65–67,74^). Overall, during ripples, superficial CA1 principal cells exhibit stronger changes in firing activity compared to deep cells ^37,49,51,67^. Here, we show that while Rad^sink^ ripples reflect the canonical pattern of superficial cells being more active than deep cells, LM^sink^ ripples exhibit the opposite trend, with deep cells being predominantly more active than their superficial counterparts. Furthermore, we found that during Rad^sink^ ripples CA1 primarily ‘aggregates’ superficial principal cells onto core LM^sink^ ripples motifs.

Importantly, while previous work showed that the offline reactivation of waking patterns in CA1 is mainly driven by superficial cells ^67^, we observed that this is not the case during LM^sink^ ripples. CA1 superficial cells and CA3 principal cells were not significantly reactivated during these events (Figures 6G and S7B). These findings align with earlier reports showing that CA1 deep cells receive stronger inputs from EC3, which projects to *stratum lacunosum-moleculare*; while CA1 superficial cells receive stronger inputs from CA3, which projects to *stratum radiatum* ^38,51,56,75^. Accordingly, we suggest that during Rad^sink^ ripples, offline reactivation of waking patterns primarily engages CA3 ensembles, which in turn bias CA1 to reactivate the associated higher-dimensional response integrating superficial CA1 cells.

Deep and superficial CA1 principal cells have also been shown to respond differently to behavioural experiences. Notably, in theta cycles marking wakeful exploration, deep cells exhibit higher firing rates and more stable activity patterns, whereas superficial cells show lower firing rates and adapt flexibly to new experiences ^37,49,67^. This difference could be partly attributed to stronger excitatory inputs that superficial cells receive from CA3, enabling them to respond to contextual changes ^38,51,59^. Consistent with these findings, we show that superficial cells drive in Rad^sink^ ripples of post-exploration sleep the expression of coactivity motifs expressed during the preceding exploratory behaviour. In contrast, the more rigid deep cells instantiate pre-existing motifs of coactivity in LM^sink^ ripples. Previous studies reported that CA1 superficial, but not deep, principal cells undergo postsynaptic potentiation in response to CA3 inputs after novel experience ^59^. These results suggest that CA3 enables CA1 superficial cells to rapidly and flexibly reorganise coactivity patterns in Rad^sink^ ripples following new waking experience. Conversely, the reduced activity of CA3 during LM^sink^ ripples could favour the expression of deep cell motifs.

### Ripple diversity for balancing integration of recent experiences and preservation of prior representations in the hippocampus

Why does the offline hippocampus co-process rapid integration of recent population responses and gradual adaptation of prior population responses? While the prompt integration of new information is essential, maintaining a stable underlying population activity structure could yet provide a “backbone” upon which finer-grain information processing can be built. Recent theoretical work suggests that the hippocampus integrates new sensory information using a pre-structured internal scaffold provided by EC, enabling scalable, flexible, and efficient memory storage ^76^. In line with this, the core motifs of deep cells associated here with LM^sink^ ripples would define such a pre-structured scaffold. Recent waking experience would then flexibly recruit coactivity motifs of CA1 superficial cells within the network. Through CA3-driven theta-nested activity, the network is transiently pushed out of the prior neural backbone to append new information (Figure 7G). Combining deep and superficial CA1 principal cells would allow enhancing the specificity and distinctiveness of newly-acquired representations. The resulting composite population patterns would be later reactivated offline, particularly during Rad^sink^ ripples. Meanwhile, LM^sink^ ripples would also continue expressing core activity motifs, which gradually drift to update the population backbone. Deep and superficial cells would therefore work synergistically towards the concomitant rapid integration and gradual refinement of hippocampal population representations. Moreover, the observed differences between ripple types in the offline dynamics of recent versus prior motifs suggests that these changes are not driven by global changes in the behavioural sleep state.

The prior coactivity motifs nested in LM^sink^ ripples appear more stable and yet not stationary over the hour-long sleep following recent wakefulness. Population patterns in LM^sink^ ripples, but not those in Rad^sink^ ripples, gradually drift toward the coactivity motifs defined during the recent waking activity. The reactivation strength of pre-exploration sleep coactivity during LM^sink^ ripples gradually decreased over the course of post-exploration sleep, while reactivation of recent motifs remained stable and significant, although weaker than during Rad^sink^ ripples. This suggests that the structure of neuronal coactivity motifs is temporally refined in LM^sink^ ripples, drifting away from the prior motif backbone to more closely resemble recent motifs, progressively increasing on a ripple-by-ripple basis the expression ratio of recent to prior motifs throughout sleep. Importantly, temporal dynamics in the coactivity among CA1 deep principal cells better explained this drift compared to those among superficial ones. This might by associated to the complex input-output relationships established by superficial and deep CA1 principal cells, and the possibility of bidirectional influences. CA1 superficial cells predominantly project to EC ^38,56,63,67,75^; CA1 deep cells preferentially receive inputs from EC. This could actuate a feedback loop to CA1 deep cells. CA1 superficial cells could also modulate deep cells by recruiting local inhibitory circuits (e.g., parvalbumin-expressing basket cells ^51,77^). By these means, CA1 superficial cells would influence both the hippocampal-entorhinal and the intra-hippocampal circuitries, shaping the core motifs of deep CA1 cell activity embedded in LM^sink^ ripples. That is, during post-exploration sleep, motifs of superficial cells reflecting recent waking experience reactivate in Rad^sink^ ripples. This could in turn bias EC activity, which projects back to CA1 deep cells, gradually shifting the CA1 deep motifs toward the most recent activity state space. This interactive process could help internal linking of the recent experience with previous ones in the hippocampus, and allow network updating with the most recently encountered information ^78,79^. Moreover, CA1 deep cells project to neocortical areas (prefrontal cortex) ^67^. The gradual drift of the hippocampal backbone, evolving from prior to recent coactivity spaces over hour-long post-exploration sleep/rest, could exert important influence on systems consolidation of neuronal ensembles in downstream neocortical circuits (e.g., prefrontal cortex ^67,80^). In parallel, this gradual drift would also update the local neuronal priors within the hippocampus, which would then serve as the foundation for the next low-dimensional backbone in LM^sink^ ripples, while Rad^sink^ ripples, following recent experience, continue to push the network out of this state through superficial cells, with high-dimensional patterns reporting recent wakefulness. Such a functional loop could support a network trade-off between stability and flexibility.

### Limitations of study

This study focused on offline CA1 ripples occurring during hour-long periods of sleep/rest. The number of awake ripples detected during exploration was insufficient to reliably characterise their laminar current profiles. CA2 contributes more strongly to awake ripples whereas CA3 participates more to sleep ripples ^81^. This may account for the very small subset ripples associated with a dominant current sink in stratum oriens, the main CA1 target of CA2 projections. Future work could investigate whether specific CSD profiles emerge in awake ripples, potentially revealing a more substantial subset of CA2-associated ripple types. This would require a high number of events to capture ripple-by-ripple variability robustly. In conditions with few detected ripples, it remains difficult to distinguish genuine diversity from sampling limitations.

The findings presented here are observational in nature. The specific hippocampal and extra-hippocampal circuits responsible for generating Rad^sink^ and LM^sink^ ripples remain to explore. Our results provide evidence that Rad^sink^ ripples involve more CA3 inputs while LM^sink^ ripples might be more closely associated with EC inputs to CA1. Establishing a causal link between these inputs and ripple profiles would require targeted manipulations. This approach remains complicated by the dynamic interactions between hippocampal and EC regions. In line with this, we observed that the occurrence of both Rad^sink^ and LM^sink^ ripples was generally higher during epochs of strong EC input (as inferred from the DG CSD signals), suggesting that both ripple types are driven, at least in part, by cortical inputs Moreover, LM^sink^ ripples are characterised by two temporally distinct current sinks around the ripple peak: an early, stronger sink in lacunosum-moleculare followed by a late sink in radiatum coinciding with increased CA3 activity. This suggests that CA3, the primary input during Rad^sink^ ripples, also contributes to LM^sink^ ripples as their second sink of current. Moreover, CA1 projects back to the EC, potentially influencing the trisynaptic loop, including CA3. This reciprocal connectivity makes it likely that manipulating either CA3 or EC affects the other, complicating any attempt to dissociate their respective roles in generating Rad^sink^ versus LM^sink^ ripples. Together, these findings highlight the complex interplay between intra-hippocampal and cortical inputs in shaping ripple dynamics, and caution against attributing ripple generation to a single region using standard manipulation techniques. Moreover, other regions (e.g., the nucleus reuniens ^82,83^) project to stratum lacunosum-moleculare, constituting a potential further input underlying ripple current sinks. Therefore, a more refined approach to probe for the role of specific inputs would involve closed-loop, ripple-type-specific interventions guided by real-time classification of laminar CSD profiles. Future work could develop such technology not yet available, leveraging the findings of the current study on ripple diversity for causal manipulations testing circuit mechanisms and functions of Rad^sink^ and LM^sink^ ripples.

## Conclusion

These findings underscore the diversity in the expression profiles of a given network pattern (e.g., CA1 ripples), which along the heterogeneity within a given neuronal population (e.g., CA1 principal cells), can instantiate parallel processing channels in the hippocampus. Here, Rad^sink^ ripples nest hour-long, temporally stable reactivation of recent waking population patterns, integrating recently recruited superficial cells with core activity motifs of deep cells. In contrast, LM^sink^ ripples nest time-varying reactivation of prior population patterns, undergoing hour-long gradual drift that updates core activity motifs with recent waking experience. This pre-existing hippocampal backbone could not only preserve a coherent population activity structure but also facilitates automatic integration of new waking information. Organising distinct waking experiences within a common “schema” could support flexible computations and further reduce energy demand compared to a framework imposing the *de novo* creation of entirely new high-dimensional population patterns from the outset each time ^76,84–86^. We propose that ripple-by-ripple diversity leverages differences in the activity level, structural organisation, and neuronal content of population patterns for parallel offline reactivation of prior versus recent activity in the hippocampal system.

## Resource Availability

### Lead contact

Further information and requests for resources and reagents should be directed to and will be fulfilled by the lead contact, David Dupret (david.dupret@bndu.ox.ac.uk).

## Materials availability

This study did not generate new unique reagents

## STAR Methods

### Experimental Model And Study Participant Details

#### Animals

These experiments used adult (4–6 months old) male C57BL/6J wild-type mice (Charles River Laboratories, UK). Animals were housed with their littermates up until the start of the experiment. All mice were held in IVCs, with wooden chew sticks and nestlets in a dedicated housing facility with a 12/12 h light/dark cycle (lights on at 07:00), 19–23°C ambient temperature and 40–70% humidity. They had free access to water and food *ad libitum* throughout the experiment. Experimental procedures were performed on mice in accordance with the Animals (Scientific Procedures) Act, 1986 (United Kingdom), with final ethical review by the Animals in Science Regulation Unit of the UK Home Office.

### Method Details

#### Surgical procedure

All surgical procedures were performed under deep anaesthesia using isoflurane (0.5–2%) and oxygen (2 l/min), with analgesia provided before (0.1 mg/kg vetergesic) and after (5 mg/kg metacam) surgery.

For silicon probe recordings, mice were implanted with a single-shank silicon probe (Table S1) under stereotaxic control in reference to bregma, using central coordinates -2.0 mm anteroposterior from bregma, +1.7 mm lateral from bregma, and an initial depth of 1.5 mm ventral from the brain surface to span the somato-dendritic axis of CA1 principal cells and reach the DG. Following the implantation, the exposed parts of the silicon probe were covered with Vaseline® Healing Jelly, after which its plastic drive was secured to the skull using dental cement and stainless-steel anchor screws inserted into the skull. Two of the anchor screws, both above the cerebellum, were attached to a 50 μm tungsten wire (California Fine Wire) and served as ground. For the recordings, the silicon probe was positioned along the radial axis of CA1 pyramidal cells, using the rotations applied to its holding screw.

For tetrode recordings, mice were similarly implanted with a single microdrive containing 14 independently movable tetrodes, each positioned to target the *stratum pyramidale* of either CA1 or CA3 in the dorsal hippocampus. Tetrodes were constructed by twisting together four insulated tungsten wires (12 μm diameter, California Fine Wire) which were briefly heated to bind them together into a single bundle. Each tetrode was loaded in one cannula attached to a 6 mm long M1.0 screw to enable its independent manipulation of depth. The drive was implanted under stereotaxic control in reference to bregma using the following coordinates. For CA1 pyramidal cell layer tetrodes, the span was between AP -2.0 to –2.4 mm and ML 1.6 to 2.3 mm. For CA3 pyramidal cell layer tetrodes, the span was between AP -1.8 to –2.2 mm and ML 2.0 to 2.7 mm. The initial depth of the tetrodes during the implantation surgery was 1.0 mm ventral from the brain surface. The distance between neighbouring tetrodes was 350 μm. Following the implantation, the exposed parts of the tetrodes were covered with paraffin wax, after which the drive was secured to the skull using dental cement and stainless-steel anchor screws inserted into the skull. Two of the anchor screws, both above the cerebellum, were attached to a 50 μm tungsten wire (California Fine Wire) and served as ground. For the recordings, each tetrode was lowered along the vertical axis to reach either the CA1 or CA3 pyramidale layers, using the rotations applied to its tetrode cannula-holding screw and the electrophysiological profile of the local field potentials in the hippocampal ripple frequency band, with final depth position subsequently confirmed by histology of anatomical tracks.

#### Recording Procedure

Following implantation surgery, mice were allowed to recover for at least seven days. They were then familiarized with the recording procedure, being handled daily in a dedicated towel, connected to the recording cable and exposed to the sleep box for at least 30 minutes per day for a minimum of four days. During this period, both silicon probes and tetrodes were gradually lowered toward the target cell layers. For silicon probe implants, probes were positioned to target the CA1 region, and once the appropriate depth was reached, they were left in place for the remaining days of the experiment. For tetrode implants, tetrodes were adjusted every day to target the CA1 and CA3 pyramidal cell layers. Electrophysiological profiles, including local field potential characteristics such as sharp-wave ripples and gamma oscillations, were used to guide daily placement. On each recording day, tetrodes were lowered into the target layers in the morning to capture ensemble spiking activity and left in place for approximately 1.5–2 hours before recordings began that day. At the end of the recording day, tetrodes were raised by approximately 150 μm to prevent mechanical damage to the hippocampal layers. The following morning, tetrodes were re-adjusted to locate new cells, minimizing the likelihood of recording from the same neurons across days.

Each recording day began with a baseline sleep/rest session (pre-exploration sleep/rest), followed by an exploration session to finish with a post-exploration sleep/rest session (post-exploration sleep/rest). The environments used in these recordings were either open-field arenas for exploration sessions (e.g., 41 cm diameter cylinder, 41 × 41 cm square box; all with 30 cm high walls) or the sleep box (12 × 12 × 28 cm; containing sawdust bedding and nesting material). After placing the mouse in the sleep box, experimenters monitored the animal’s movements and real-time raw electrophysiological signals to confirm that the mouse had started to sleep/rest. In the absence of electromyographic or any other signals (e.g., neuromodulator levels, respiratory patterns) to categorize sleep stages, we refer to these offline periods of extended immobility as sleep/rest. Exploration sessions lasted ~30 minutes; sleep/rest sessions lasted ~60–90 minutes. All experiments were conducted under dim light conditions (~20 lux) with low-level background white noise (~50 dB). In total, the silicon probe dataset included 38 sleep sessions [mean duration (IQR): 82.4 (59.5 – 105.9) minutes per sleep/rest] from 5 mice; the tetrode dataset included 244 sleep sessions [mean duration (IQR): 68.8 (48.0 – 90.2) minutes per sleep/rest] from 13 mice.

#### Acquisition of multichannel data and tracking of animal position

Extracellular signals were recorded using an integrated circuit mounted on the animal’s head (model RHD2164, Intan Technologies; http://intantech.com/products_RHD2000.html), which provided a frequency response from 0.09 Hz to 7.60 kHz during the amplification stage. The amplified and filtered signals were digitised at a sampling rate of 20 kHz. These digitised signals were stored alongside additional data streams, including digital pulses indicating the animal’s position (via transistor-transistor logic) and signals from a three-axis accelerometer integrated into the head-mounted device, which measured head movements and provided an additional measure of the animal’s movement. Positional data were obtained using an overhead colour camera (https://github.com/kevin-allen/positrack/wiki), which tracked the movement of LED clusters in three distinct colours affixed to the electrode assembly. These positional signals were captured at a rate of 39 frames per second.

#### Processing of Local Field Potential (LFP) signals

LFP signals underwent initial filtering using an 8th-order Chebyshev type I anti-aliasing filter, applied to the wide-band signals sampled at 20 kHz. These filtered signals were then downsampled to a rate of 1,250 Hz, employing the decimate function within Scipy’s signal submodule (version 1.11.2).

#### Sharp-wave/ripple (SWR) event detection

LFPs were first referenced to a channel without CA1 ripples. This differential signal underwent dual stage-filtering: through a ripple-specific bandpass filter (80-250Hz, 4th-order Butterworth Filter), and then through a high-frequency bandpass filter (200-500Hz, 4th-order Butterworth Filter). Instantaneous signal characteristics, including envelopes and phases, were derived using the Hilbert transform. SWR events were identified by detecting envelope peaks within the ripple band that exceeded a threshold of five times the median value. In instances of multiple peaks within a 20-ms window, only the peak with the highest amplitude was considered. For each event we then identified its onset and offset points as the points where the envelope fell below half of the established threshold. Analysis extended to quantifying the ripple cycle count within each event by examining phase shifts, with cycle calculation based on the unwrapped phase difference between event onset and offset as previously described ^37^. The mean frequency of each event was calculated by dividing the total cycle count by the event’s duration. Finally, we validated candidate SWR events using four criteria: (1) Ripple band power in the detection channel, calculated as the squared mean amplitude, needed to be double that of the reference channel, ensuring detected events were prominent; (2) The mean frequency of detected events must exceed 80 Hz; (3) Each event should contain at least four complete ripple cycles; (4) Ripple band power should be at least double that of the control high-frequency band. We focused this study to ripples detected during sleep/rest epochs, as too few ripples were detected during exploratory behaviour [median (IQR) number of detected awake ripples across both the silicon probe and tetrode datasets: 17 (4 – 53) events; n = 230 exploration sessions]. All detected ripples were included in the analyses unless stated otherwise, as appropriate [mean number of detected ripples (IQR): 3,341.6 (2,130.3 – 4,413.5) per sleep/rest]. Namely, for specific analyses requiring preventing influences by overlapping events (which would otherwise contaminate average signals), only isolated ripples were used (i.e., ripple events with no other detected ripple occurring within ±250 ms). This applies to ripple-triggered average signals (e.g., the triggered-average CSD in Figures 1C and S2F-I; the peri-ripple cells’ response in Figure 4B). All the ripple-triggered average signals were referenced to the ripple-envelope peak unless stated otherwise.

#### Determination of CA1 pyramidal layer reference channel

To identify the optimal reference channel for SWR events and theta oscillations within the CA1 pyramidal layer, we computed a ripple band score for each channel. This score was calculated by dividing the power in the ripple band (80–250Hz) by the power in an adjacent frequency range (70– 300Hz), using a Welch spectrum (4-second Hann windows overlapping by 50%). The channel with highest score was set as a reference channel within the CA1 pyramidal layer.

#### Extraction of theta oscillations from LFPs

To isolate theta oscillations from the LFP data in exploration sessions, we employed the masked Empirical Mode Decomposition method ^87,88^ as implemented by Quinn et al ^89^. With this, we adopted the mask sift procedure using specific mask frequencies set at 350, 200, 70, 40, 30, and 7 Hz, following the parameters optimized in ^90^ grounded in ^91^. For each mask, the amplitude was set to three times the standard deviation of the input signal. This procedure decomposes each LFP signal into oscillatory components termed intrinsic mode functions (IMFs) from faster to lower frequency components. Upon completion of this procedure with the parameters mentioned, six IMFs and a residue were computed, with IMF-6 effectively isolating theta oscillations.

To delineate individual theta cycles, we began by pinpointing peaks and troughs (i.e., the local maxima and minima, respectively) of the obtained theta IMF. The residue of the LFP not captured by the six IMFs was defined as the lower frequency component of the signal and its envelope was used as the amplitude threshold for retaining peaks and troughs for the next step. We then defined each peak-trough-peak sequence as a candidate theta cycle. We took as valid cycles sequences having their peak-trough and trough-peak intervals falling within the 31 to 100 ms range (corresponding to the half period of cycles with frequencies ranging from ~16 to 4 Hz); and peak-to-peak distance was between 71 ms (equivalent to ~14 Hz) and 200 ms (equivalent to 5 Hz).

For each validated cycle, we found six control points: the zero-crossing prior to the first peak, the peak itself, the subsequent zero-crossing post the first peak, the trough, and the zero-crossing following the trough. Then, we computed the instantaneous theta phase for each timestamp through a linear interpolation of the control points ^73,92^.

#### Wavelet Spectrograms

The spectrograms shown in Figure S2H were generated using the complex Morlet Wavelet Transform. For this analysis, 50 logarithmically spaced frequencies were selected, spanning from 2 Hz to 300 Hz unless otherwise specified. Each wavelet kernel was L1-normalised, meaning the sum of the absolute values of the elements in the kernel was set to 1. This normalisation ensured that the wavelet preserved the relative amplitudes of individual frequency components without amplifying or attenuating them.

#### Current source density analysis

We applied CSD analysis ^93,94^ to event-triggered LFP signals obtained using linear silicon probes. For ripple-related analyses, we centred LFP signals around the ripple-band envelope peak. Upon configuring these event-based signals, we calculated the CSD at a specific channel *n* and time point using the formula:

$$CSD_{n}=-\left(LFP_{n-1}-2\cdot LFP_{n}+LFP_{n+1}\right)$$

where *n* − 1 and *n* + 1 represent the channels directly above and below channel *n*, respectively. This way, for each ripple event we obtained its CSD signals. In this study, we focused on CA1 channels. We defined the location of *oriens, radiatum and lacunosum-moleculare* layers according to the ripple and sharp-wave laminar profiles and electrode spacing, as previously described ^37^. To ensure uniform spatial resolution of CSD measurements across silicon probes with different channel spacing (Table S1), we applied Gaussian kernel smoothing with a standard deviation parameter set to 50 *μm*. For the ripple-triggered average CSD signals in Figures 1C and S3A-E, we only included isolated ripples (see section ‘Sharp wave-ripple (SWR) event detection’).

#### Single-ripple CSD signatures

To obtain the CSD signature of each ripple, for each channel we computed the mean CSD signal within a 50-ms window around the ripple envelope peak. This way, for each ripple event we obtained a curve that described the average CSD signals during that ripple. We defined this curve as the CSD signature of this ripple event. To identify the dominant sink layer for each ripple, we checked which of the oriens, pyramidale, radiatum, or lacunosum-moleculare strata had the most negative value in the ripple’s CSD signature (Figure S1D,E). For example, a ripple was classified as having the dominant sink in radiatum if its CSD in that layer was negative and lower than in all other layers.

#### Principal component analysis of single-ripple CSD signatures

To analyse the variance across CSD signature profiles, we applied principal component analysis (PCA). For each recording session, we computed the variance explained by each extracted principal component (PC) as:

$$\text{Variance}\;\text{Explained}=\frac{\sigma_{i}^{2}}{\sum_{j=1}^{m}\sigma_{j}^{2}}$$

where σ_*i*_ is the singular value associated of the *i*^*th*^ PC and *m* is the total number of PCs extracted. To ensure consistency in the sign of PCs across different recording sessions, we adjusted the sign of the first PC so that it consistently exhibited negative weights within the *stratum radiatum*.

#### Identification of Rad^sink^ and LM^sink^ profiles from single-ripple CSD signatures

To investigate the variations in CSD profiles modulated by the first PC across different ripple events, we categorized the distribution of PC1 strengths into three groups, focusing on the extremes of the distribution to capture the most pronounced CSD signatures. Specifically, since PC1 was adjusted to exhibit negative weights in the *stratum radiatum* (indicating that a higher PC1 value corresponds to a stronger current sink in this layer), the ripples with PC1 strengths surpassing the 70th percentile were classified as Rad^sink^ events. Conversely, ripples with PC1 strengths falling below the 30^th^ percentile were designated as LM^sink^ events. The remaining ripples were placed in an ‘intermediate’ event category (e.g., used in Figure 3C,E and S2F-I,T).

#### Occurrence distribution of Rad^sink^ and LM^sink^ ripples in NREM sleep

To assess the temporal distribution of Rad^sink^ and LM^sink^ ripples during NREM sleep, we first delineated individual NREM bouts. For this, we computed the theta-power ratio from the CA1 pyramidal layer channel using short-time Fourier transform (scipy.signal.spectrogram), with a 2-s window and 90% overlap. The ratio was defined as the power in the theta band (5–10 Hz) divided by the total power in the 0.1–10 Hz range. We then downsampled the resulting time series to 1 Hz to extract its slower component, generating a signal that tracked relative theta power throughout the sleep session. Visual inspection (e.g., Figure S2Q) confirmed that epochs with low and high theta-power ratios corresponded to NREM sleep and REM sleep (or awake epochs), respectively, in line with the behavioural states labelled during the recording procedure (see section ‘Recording Procedure’). To distinguish NREM from REM (or awake) epochs, we applied a two-component Gaussian mixture model to the distribution of this theta-power ratio and additionally required maximum tracking speed to fall below 1.5 standard deviations from the session mean (~1.5–2 cm/s). REM and awake epochs were then distinguished based on animal speed in each temporal window, with REM epochs defined as periods that (1) had a maximum speed below the above-mentioned threshold and (2) followed a NREM epoch lasting at least 2 minutes.

We divided each identified NREM epoch into five equally spaced bins thus using a normalized time axis. For each bin, we calculated how ripples of each type were distributed by computing their proportion relative to the total number of that type across the entire NREM epoch. Only NREM bouts lasting at least 10 seconds were included in this analysis. To test whether the rate of each ripple type changed over NREM time, we concatenated the NREM ripple proportion curves from all sleep sessions and tested whether they were significantly correlated with NREM normalized time. Statistical significance was determined by comparing the observed correlation to a null distribution obtained by shuffling NREM time 10,000 times. From the whole silicon probe dataset, three out of five mice had tracking data available to be able to perform these analyses.

#### Control analyses for ripple frequency differences

In Figure S2M,N, we performed two analyses to control for the possibility that the observed difference in Rad^sink^ and LM^sink^ ripple frequency could be explained by rest/sleep transitions.

First, for each session lasting at least 60 minutes (excluding the initial 10 minutes of each session to account for potential rest-to-sleep transitions), we computed the time-resolved difference in ripple frequency between Rad^sink^ and LM^sink^ events (Figure S2M). We then assessed whether this difference changed over time by computing the correlation between the ripple frequency difference and the corresponding time bins. The resulting correlation values were compared against a null distribution obtained by shuffling the time bins.

Second, in Figure S2N, we recomputed ripple frequency estimates using only those events occurring within the five minutes preceding REM onset (see Methods: ‘Occurrence distribution of Rad^sink^ and LM^sink^ ripples in NREM sleep’), a period during which rest-to-sleep transitions are highly unlikely. This analysis was performed on sleep sessions where at least one bout of REM sleep was detected.

#### Rad^sink^ versus LM^sink^ sequential order

To investigate whether Rad^sink^ and LM^sink^ ripples co-occur interchangeably or tend to cluster in bursts of events, we modelled ripple types as a first order Markov process in which each event type (Markovian state). This framework captures the temporal structure of ripple occurrence by quantifying the transition probabilities between types (i.e., the likelihood of observing a given ripple type after another). These values fall in a range from 0 to 1, inclusive. A probability of 0 means the transition is impossible, and a probability of 1 indicates it is certain. For each state, the sum of transition probabilities to all states (including remaining in the same state) must equal to 1. We first identified chains of ripples in which successive events occurred within 250 ms of one another. Across sleep sessions, these chains most commonly consisted of two ripples, with the likelihood of longer chains declining monotonically (Figure S2S). Within each chain, we classified ripple events as Rad^sink^, intermediate, or LM^sink^, and used these labels to compute the probability of observing a ripple of type *n* given a preceding ripple of type *m*. These values were used to construct ripple-type transition probability matrices (Figure S2T). To account for class imbalance in the number of ripples per type, we generated surrogate transition matrices by randomly shuffling ripple-type labels while preserving the total number of events per class. This procedure disrupted the temporal structure while maintaining the overall class proportions. The shuffling was repeated 1,000 times, and each entry of the observed transition matrix was normalised by subtracting the corresponding mean value from the surrogate distribution.

#### Assessment of cortical Up/Down states

To test whether Rad^sink^ and LM^sink^ ripples are differentially distributed within cortical Up/Down states, we estimated the CSD energy within the molecular layer of the dentate gyrus (DG) as a proxy for EC input strength to the hippocampus ^11,39,40^. Specifically, we computed the DG molecular CSD energy as the average rectified CSD across recording channels in this layer. The resulting signal was smoothed with a 1-second median filter, downsampled to 5 Hz, and z-scored. This time series exhibited a bimodal distribution (Figure S3A). To identify Up and Down states, we fit a two-component Gaussian Mixture Model to this distribution using the GaussianMixture class from the sklearn.mixture module. The state probabilities from this mixture model were used to initialise the emission probabilities of a two-state Hidden Markov Model (HMM), implemented using the hmmlearn.hmm class from the hmmlearn package. The HMM was then trained on the downsampled DG molecular CSD signal using the Viterbi algorithm to infer the most likely sequence of Up and Down states. This procedure allowed segmenting each sleep session into epochs of high and low DG molecular CSD energy, which we refer to as Up^DG^ and Down^DG^ states, respectively (Figures 2B and S3A). These Up^DG^ and Down^DG^ epochs had comparable durations across sleep sessions [mean duration (IQR): Up^DG^: 5.74 (3.6 – 7.4) seconds; Down^DG^: 4.4 (3.4 – 4.2) seconds; p = 0.13; paired bootstrap test; n = 33 sleep/rest sessions], consistent with previous reports^39^. We next identified Up^DG^ → Down^DG^ and Down^DG^ → Up^DG^ transitions, considering only states changes preceded and followed by epochs lasting at least 500 ms (Figure S3B). For this analysis, we included only sleep sessions in which silicon probe spanned from the outer molecular layer to the granule cell layer of the DG, and in which the algorithm detected at least 100 Up and 100 Down states. This yielded a total of 26 sleep sessions from 5 mice. To examine how the proportion of LM^sink^ ripples varies with the DG molecular energy, we computed, for each session, their relative proportion across equally spaced bins of the z-scored DG molecular CSD energy (Figure 2D).

#### Explained variance of ripple LFPs by laminar CSD

In Figure 3B, we quantified the extent to which the ripple-by-ripple variations in the LFP waveforms from a given recording site placed in the CA1 *stratum pyramidale* can be accounted for by their underlying CSD across different CA1 layers, from *stratum oriens* to *stratum lacunosum-moleculare*. To this end, we trained linear decoders to predict for each individual ripple the strength and the sign of the CSD marking each CA1 layer from the LFP recorded in the *stratum pyramidale*. Each ripple-nested *stratum pyramidale* LFP trace was low-pass filtered through a Butterworth filter (4th order, with a cut-off frequency of 30 Hz) to focus specifically on the low-frequency component reflecting the ‘sharp-wave’, which we hypothesized contained all the information to distinguish the underlying current profiles. Then, the filtered LFP waveforms were standardized through z-score transformation to ensure uniformity in variance and mean across the signals of all ripples. We then applied principal component analysis to reduce the dimensionality of the 200-ms LFP traces down to six principal components, which explained more than 80% of the variance of all ripple-LFP waveforms (Figure S3I). Similarly, the CSD signals for each CA1 layer (*strata oriens, pyramidale, radiatum*, and *lacunosum-moleculare*) were normalized by dividing each CSD signal by its standard deviation calculated over multiple events. This normalization allowed maintaining the polarity information of the CSD signals while ensuring comparability across different magnitudes.

Subsequently, we employed linear regression models to predict the normalized CSD in each CA1 layer from the dimensionality-reduced LFP signals during ripple events. To validate the robustness and generalizability of our models, we performed a cross-validation 20 times (80% training, 20% testing) on the LFP and CSD data. For each iteration, the model was fitted to the training set and then evaluated on the testing set, thereby obtaining a coefficient of determination *R*^2^ for each run. The coefficient of determination, *R*^2^, was calculated as:

$$R_{\text{layer}}^{2}=1-\frac{\sum_{i=1}^{n}{\left(y_{i,\text{layer}}-\hat{y_{l,\text{layer}}}\right)}^{2}}{\sum_{i=1}^{n}{\left(y_{i,\text{layer}}-{\bar{y}}_{\text{layer}}\right)}^{2}}$$

where *y_i_,layer* and $\hat{y_{l,\text{layer}}}$ respectively represent the actual and the predicted CSD value for the *i* -th ripple in the specified ‘layer’; ${\bar{y}}_{\text{layer}}$ is the mean of the actual CSD in the specified ‘layer’; *n* is the total number of ripples in each recording session. The variance explained by each model was then determined by averaging the *R*^2^ values across all 20 cross-validation iterations, providing a measure of how well the CSD can be predicted from the LFP signals across the different CA1 layers. This cross-validated approach allowed us to assess the predictive power and reliability of our linear decoders in explaining the variance in CSD strength and sign from the LFP recordings during ripple events. The chance level of explained variance of the LFPs was determined by shuffling 500 times the true CSD values and then computing the variance explained by the shuffled data.

To control for the possibility that differences in explained variance of ripple LFPs were driven by rest/sleep transitions, we repeated the above procedure separately for the first and second half of the detected ripples in each recording session lasting at least 60 minutes (Figure S3C).

#### Structure index of LPF waveforms

Using the structure index measure ^45^, we further validated (in Figure S3D) the observed relationship between the ripple-triggered LFP signals recorded in the pyramidal cell layer and the CSD signals obtained in each CA1 layers (in Figure 3B). For each sleep/rest session, we first applied the same pre-processing steps to the LFP and CSD signals described above (see ‘Explained variance of ripple LFPs by laminar CSD’). Then, we computed the structure index for each layer to quantify the interplay between the time profiles of the pyramidal LFPs and the CA1 layer-selective CSD magnitude during ripples. To evaluate the significance of our results, chance levels were determined by shuffling the data 500 times as previously described ^45^.

#### Single-ripple CSD profile prediction from pyramidal LFP waveform

We applied Linear Discriminant Analysis (LDA) to predict both the polarity (sign) and the magnitude of the *lacunosum-moleculare* (LM) current for each ripple event based on the corresponding *stratum pyramidale* LFP time course. Ripple LFPs waveforms from the pyramidal layer were extracted and pre-processed as explained in section ‘Explained variance of ripple LFPs by laminar CSD’ (n = 117,658 isolated ripples from five mice; see Figures S3E-I). To classify each ripple event into the Rad^sink^ versus LM^sink^ category, the corresponding LM CSD was computed within a 50-ms window centred on the ripple peak. This CSD was averaged over three adjacent probe channels: one in *lacunosum-moleculare* and the channels immediately above and below it. To ensure comparability across sleep sessions while retaining information about current polarity, the ripple CSD was normalised by the standard deviation of all ripple events. Ripple events with a normalised negative CSD in the lowest 30th percentile were classified as LM^sink^ ripples, while those in the top 30th percentile were classified as Rad^sink^ ripples. Ripples with CSD values within the 30–70th percentile range were classified as ‘intermediate’ ripples (Figure S3E-I), characterised by relatively small current magnitudes.

To assess the robustness of the LDA model, we employed a leave-one-out cross-validation procedure, where each mouse was excluded from the training set in turn, and the model’s accuracy was evaluated on the left-out mouse. To address class imbalance in the training data, we downsampled each class to match the size of the smallest class, repeating this balancing procedure 1,000 times. For each permutation, we also trained a null model with shuffled training labels, disrupting the relationship between pyramidal LFP waveforms and LM currents to estimate chance-level accuracy. In each iteration, we computed the accuracy of both the true model and the null model on the testing data for the left-out mouse. This process provided a chance-normalised accuracy for each left-out mouse, calculated by comparing the mean accuracy of the true models to that of the null models (Figure 3D).

To further validate that the model could recover the expected CSD sink polarity and amplitude, we computed the mean CSD in stratum radiatum and lacunosum moleculare for ripples classified as Rad^sink^, intermediate, and LM^sink^ during each leave-one-out iteration (Figure 3E). As the ripple envelope peak does not coincide with the timing of current sinks, we used the training set to identify the time point of the maximal sink. We then aligned test-set events accordingly: LM^sink^ ripples were aligned to the deepest sink in lacunosum-moleculare, whereas Rad^sink^ and intermediate ripples were aligned to the deepest radiatum sink. Mean CSD amplitude was then computed in the testing set using a 10-ms window centred on these sink-optimised time points.

To test whether the LDA classifier could reliably generalise across different radial positions within the CA1 pyramidal layer, we applied the model described above to ripple waveforms recorded from channels located ±60 μm from the pyramidal layer centre (see Table S1) and evaluated performance against the CSD-based ground-truth labels (Figure S3K). We specifically assessed the classifier’s ability to discriminate between Rad^sink^ and LM^sink^ ripples, as these two profiles represent the two ends of the embedding continuum (Figure 3A and S3E-I). For each recording day, we concatenated all ripple waveforms recorded and then applied the model for prediction. These predictions were used to compute a discrimination index defined as the ratio of correctly classified Rad^sink^ and LM^sink^ ripples to those misclassified as the opposite type (e.g. a true Rad^sink^ predicted as LM^sink^). To obtain a conservative estimate of classification performance, we took the lower of the two values as the session’s discrimination index. This procedure was repeated across different recording depths within the pyramidal layer, allowing us to assess how classification performance varied as a function of radial position.

When this model was applied to the tetrode dataset, the pyramidal layer tetrode used for ripple classification was picked as the one with average LFP waveform most similar to the average waveform from the model’s training set. Applying this model to the tetrode dataset resulted in a total of 259,273 Rad^sink^ and 211,505 LM^sink^ ripples from 13 mice [mean number of ripples per sleep/rest (IQR): Rad^sink^, 1,062 (677.8 – 1,417.0); LM^sink^, 866.8 (490.8 – 1,146.3)].

#### Spike detection and unit isolation

Spike sorting and unit isolation used an automated clustering approach, leveraging Kilosort (https://github.com/cortex-lab/KiloSort) within the SpikeForest framework (https://github.com/flatironinstitute/spikeforest), as outlined in Pachitariu et al. (2016) ^95^ and Magland et al. (2020) ^96^. For data acquired using tetrodes, KiloSort’s algorithm was adapted to limit templates to channels associated with a specific tetrode bundle and to exclude all other recording channels. Data from all sessions recorded within a single day were concatenated and processed collectively, enabling continuous cell tracking across the day. The clusters generated were manually confirmed by examining cross-channel spike waveforms, auto-correlation histograms, and cross-correlation histograms. Units selected for analysis consistently exhibited stable spike waveforms, a distinct refractory period in their auto-correlation histograms, and no refractory periods in cross-correlation histograms with other units throughout the day.

#### Principal cell versus interneuron classification

Hippocampal principal cells and interneurons were distinguished using features of their spike waveforms, as described previously ^37^. Briefly, waveform consistency for each unit was evaluated using the waveform with the maximum amplitude across tetrode channels for each cluster. To quantify the prominence of a unit’s mean waveform amplitude relative to its spike-to-spike variability, we computed a waveform score:

$$wv_{score}=\sqrt{\sum_{i=1}^{n}\frac{{\left(\frac{w_{i}}{\sigma_{i}}\right)}^{2}}{n}}$$

where *w_i_*is the value of the mean waveform at sample *i*, σ_*i*_ is the standard deviation at sample *i* across all spikes, and *n* is the number of waveform samples. This metric reflects the relative magnitude of the mean waveform amplitude compared to variability across spikes. Units with a waveform score above 0.75 and fewer than 2% refractory period violations (intervals < 2 ms in the inter-spike interval distribution) were included for further analysis. Putative interneurons and principal cells were then classified based on the trough-to-peak latency of their waveforms. In a prior dataset of 4,000 neurons, the trough-to-peak latency exhibited a bimodal distribution. A one-dimensional, two-component gaussian mixture model was fitted to this distribution, and the intersection of the two components was used as the classification threshold: units with latencies above the threshold were classified as putative principal cells, and those below as putative interneurons. We applied the same inclusion criteria to the principal cells and interneurons in the tetrode dataset of this study. In total, this study includes 2,196 CA1 principal cells and 1,325 CA3 principal cells, with 408 CA1 interneurons and 333 CA3 interneurons, recorded across 83 days in 13 mice [mean number of cells per sleep/rest session (IQR): CA1 principal cells, 26.8 (15.3 – 38.0); CA3 principal cells, 19.8 (14.0 – 25.0); CA1 interneurons, 5.0 (3.3 – 6.0); CA3 interneurons, 5.0 (3.0 – 6.0)]. For analyses involving principal cell correlations or distances between population vectors across ripple types, we addressed the issue of highly sparse spike trains and imbalanced ripple counts across groups by using sleep sessions with at least 250 ripples from each group (Rad^sink^ or LM^sink^) and five principal cells with an average firing rate of at least 0.25 Hz over the entire recording day. This criterion was met by 208 sleep/rest sessions for CA1 principal cells and 171 sessions for CA3 principal cells, resulting in 1,580 CA1 and 866 CA3 principal cells across 13 mice.

#### Peri-event time histograms

In Figures 4B and S4E, we constructed Peri-event time histograms (PETHs) over 400-ms windows, spanning 200 ms on either side of the envelope peak of isolated ripples (see section ‘Sharp Wave-Ripple (SWR) Event Detection’), with a bin width of 0.8 ms. For each cell group (e.g., principal cells or interneurons in CA1 or CA3), we first computed the raw firing rate responses during Rad^sink^ and LM^sink^ ripples. These raw responses were smoothed using a Gaussian kernel (s.d. = 5 ms) and used to calculate the peak firing rate as the maximum rate within a 50-ms window centred on the ripple peak (Figures S4A,D). To visualize responses across all cells (Figures 4B and S4E), the responses were then z-scored relative to their mean and standard deviation (s.d.) during Rad^sink^ ripples and further smoothed with a Gaussian kernel (s.d. = 5 ms).

In Figure 4C, we aligned the CA1 and CA3 principal cells’ PETHs with the average CSD sinks in stratum radiatum, lacunosum-moleculare, and the middle molecular layer of the dentate gyrus from silicon probe recordings. CSD sinks were computed as the inverted CSD and normalised by dividing each session’s average sink profile by its standard deviation. Both the CSD signals and the spike times were referenced to the ripple envelope peak, enabling direct comparison between the silicon probe and the tetrode datasets.

#### Population correlation across ripple types

In Figure S4G-H, we assessed whether the neuronal populations engaged during Rad^sink^ ripples were related to those active at the pre-LM^sink^ ripple windows. For this, we first extracted the z-scored PETH (see ‘Peri-event time histograms’ section) of each cell individually and computed the mean z-scored rate within a ±25 ms window centred either 100ms before the LM^sink^ ripple peak or at envelope peak of LM^sink^ and Rad^sink^ ripples. For each cell type separately (i.e., CA1 or CA3 principal cells and CA1 or CA3 interneurons), we then computed the Pearson correlation between the resulting population vectors. Confidence intervals were estimated by bootstrapping cells (10,000 times). To reduce the impact of sparse firing, we applied the same minimum firing rate criterion described in the ‘Principal cell versus interneuron classification’ section.

#### Preferred ripple phase and phase coherence

In Figure S4B,C, we computed the coupling of CA1 principal cells to ripple oscillations during a single sleep session for each recording day. We first band-pass filtered the LFPs between 90 and 300 Hz using a 4th-order Butterworth filter and estimated the instantaneous phase of the ripple signal during each ripple event, from onset to offset. For each CA1 principal cell, we then calculated the probability of spiking relative to the local ripple phase, using the phase signal from the tetrode where the cell was recorded. The phase range was divided into 24 equally spaced bins between 0 and 2π, and the spike-phase probabilities were computed separately for Rad^sink^ and LM^sink^ ripples. From these spike-phase probabilities, we calculated the preferred ripple phase as the angular component of the mean resultant vector of the phase distribution, while the mean phase coherence was derived from the magnitude of this vector, reflecting the strength of phase locking to the ripple oscillations.

#### Interneurons to Principal cells firing ratio during Rad^sink^ and LM^sink^ ripples

In Figure S4F, we estimated the ratio of interneurons firing rate over principal cells firing rate during Rad^sink^ and LM^sink^ ripples. For both CA1 and CA3, we calculated the mean ripple firing rate of individual interneurons (*rate_interneurons_*) and divided it by the mean ripple firing rate of individual principal cells (*rate_principals_*) recorded on the same day:

$$Ratio=\log_{10}\left(\frac{{\text{rate}}_{\text{interneurons}}}{{\text{rate}}_{\text{principals}}}\right)$$

Positive values of this ratio indicated a relatively higher firing rate of interneurons compared to principal cells. This analysis was performed separately for Rad^sink^ and LM^sink^ ripples, and the resulting ratios were compared.

#### Population activity discrimination of Rad^sink^ versus LM^sink^ ripples

To determine whether the overall structure of principal cell population activity significantly differed between Rad^sink^ and LM^sink^ ripples, we trained a logistic regression model to predict ripple identity (i.e., whether an event was a Rad^sink^ or LM^sink^ ripple) based on the z-scored ripple-nested population vectors (PVs) containing spike discharge of CA1 or CA3 principal cells (Figure 4D). To control for the number of predictors across CA1 and CA3, we trained these models using multiples of 5 principal cells (i.e., 5, 10, 15, …, N, where N is the maximum number of cells divisible by 5). For each step in the number of cells, we performed 200 permutations, randomly selecting cells for the model. Each model was cross-validated 20 times (80% training, 20% testing), with accuracy measured as the mutual information between the true ripple classes and model predictions (0 bit = chance; 1 bit = perfect prediction). To account for class imbalance, we matched the number of events in each ripple class by resampling the larger class to match the size of the smaller class in each permutation. Additionally, for each step in the number of cells, we trained another model using surrogate PVs, where the coactivity of principal cells was shuffled while preserving the overall firing rate of each cell and the population rate within each ripple (see method section “Spikes shuffling control”). For each sleep session, we calculated the mean accuracy (or mutual information) across the 200 permutations as a function of the number of cells used for training. This was repeated for the shuffle control models.

These analyses were performed independently for CA1 and CA3. In Figure 4D, we compared the accuracy of the models trained with 15 principal cells.

In Figure S4I-K, we performed an alternative analysis where, instead of using the ripple-nested population vectors, we classified ripple identity based on the entire temporal pattern of spiking activity surrounding each ripple. For this, we extracted the spike train of each cell within a ±200 ms window around the ripple peak and smoothed this temporal pattern using a Gaussian kernel with a standard deviation of 3 ms. This resulted in a matrix for each cell with dimensions (*N_ripples_ x N _bins_*), where *N_ripples_* is the number of ripples and *N _bins_*) is the number of time bins in the ± 200 ms window. Principal component analysis (PCA) was then applied to this matrix to extract the first two principal components (PCs), which captured the main temporal patterns of the cell across ripples. For each cell, this produced a matrix of dimensions (*N_ripples_ x* 2) (representing the strength of the first two PCs across all ripples). These matrices were concatenated across all cells to create a larger matrix with dimensions (*N_ripples_ x* (2 · *N_cells_*)), where *N_cells_* is the total number of CA1 (or CA3) principal cells in that recording day. Each PC was then z-scored, and the resulting matrix was used as input for the classifier described above. A schematic of this method is shown in Figure S4I.

In Figure S4J, we report the accuracy of this temporal spike pattern classification as a function of the number of CA1 or CA3 cells used for training, and in Figure S4K, we compared the accuracy of CA3 and CA1 models when using 15 cells as predictors. For this analysis, to avoid contamination of the time course waveforms we used only isolated ripples (see section ‘Sharp wave-ripple (SWR) event detection’).

#### Spikes shuffling control

We developed a shuffling procedure on spike matrices that preserves both the overall firing rates of individual neurons and the total population activity across time bins while disrupting neuronal coactivity. The original matrix, where each row represents a neuron and each column represents a time bin, was shuffled to maintain the sum of spikes for each neuron (i.e., individual neuron firing rates) and the total number of spikes fired by the population (i.e., overall population firing rate) in each time bin (e.g., a given ripple). This procedure begins by calculating the total spike count for each neuron across all time bins, generating a list of indices corresponding to the times of these spikes. This list is then randomly shuffled to ensure a uniform distribution of spikes across the matrix. The shuffled spike indices are iteratively reassigned to a new matrix, preserving the total number of spikes per neuron (row) and per time bin (column) consistent with the original matrix. This approach controls for individual neuron firing rates and the overall population activity while disrupting the coactivity patterns of neurons within each time bin. This shuffling control is used in Figures 4D, 5F and S6A-C,G-I,L.

#### Neuronal coactivity graphs

To analyse the coactivity of CA1 (or CA3) principal cells during ripples, we constructed the corresponding neuronal graphs. For a given sleep session, we first computed the spike count of each principal cell within a 50-ms window centred at the time of each ripple envelope peak, yielding a matrix with dimensions defined by the number of cells and ripple events (*N_cells_, N_ripples_*). These matrices were constructed separately for Rad^sink^ and LM^sink^ ripples, and for CA1 versus CA3 principal cells. We then estimated coactivity using two methods, as described below.

Method 1: Population-conditioned coactivity. We fitted a linear regression to predict the activity neurons *j* from that of neuron *i*, while controlling for the population rate. This yield, for each pair (*i, j*) of neurons, a regression coefficient *β*_*ij*_ (Figure 5A):

$$x_{j}\sim \beta_{ij}x_{i}+\alpha_{ij}P$$

where *x_j_, x_i_* are the z-scored ripple-nested spike trains of individual neurons *j* (the target) and *i* (the predictor), and *P* is the summed activity of the other *N* − 2 neurons,

$$P=\sum_{n=0}^{N-\{i,j\}}x_{n}$$

with *α_ij_* weighting the influence of the population contribution to the activity of target neuron *j*. Hence, the recorded neurons (and their coactivity) defined the nodes (and their edges) in the coactivity graphs of each sleep session. We characterized each neuronal graph through its adjacency matrix, *A*, defined as an *N_cells_* × *N_cells_* square matrix that encapsulated the ripple-associated population-conditioned coactivity interactions across the network, resulting in a (signed and weighted) graph with no self-connections:

$$A=\left(\begin{array}{ccc} \beta_{0,0} & \cdots & \beta_{0,N}\\ \vdots & \ddots & \vdots \\ \beta_{N,0} & \cdots & \beta_{N,N} \end{array}\right)$$

with *β_i,i_* = 0 ∀*i in N*, and the additional requirement of symmetric connections $A=\frac{A+A^{T}}{2}$ forming an undirected graph.

Method 2: Fully-conditioned coactivity. In this case, we estimated coactivity by quantifying how well the activity of each cell can be predicted from the activity of all other neurons (individually, rather than as a summed activity). Specifically, for each target cell *i*, we fitted a regularised regression model (ridge regression with L2 penalty), using the Ridge algorithm from the sklearn.linear_model module, to predict its activity from all other simultaneously recorded neurons::

$$x_{i}\sim \underset{j\neq i}{\sum }\beta_{ij}x_{j}$$

These, *N_cells_* − 1 regularised regression coefficients for each neuron *i* quantified the predictive contribution of every other neuron onto *i*’s activity. These coefficients were then used to populate the rows of an adjacency matrix of fully-conditioned coactivity, resulting in a second signed and weighted graph with no self-connections:

$$C=\left(\begin{array}{ccc} \beta_{0,0} & \cdots & \beta_{0,N}\\ \vdots & \ddots & \vdots \\ \beta_{N,0} & \cdots & \beta_{N,N} \end{array}\right)$$

with *β_i,i_* = 0 ∀*i in N_cells_*. Note that *C* is not symmetric in contrast to the matrix *A* obtained from the population-conditioned coactivity.

#### Single-neuron coactivity strength

In Figure 5B and S5A-D, we defined the single-neuron coactivity strength as the average pairwise activity correlation of a given node with the other nodes in the weighted graph. As a reference, the strength in a weighted graph can be compared to the degree in a binary graph, which accounts for the number of the node’s neighbours. Here, the strength *S_i_*of a node *i* is the average across all the weights *β_ij_* of the edges projected from that node:

$$S_{i}=\frac{\sum_{j=0}^{N}\beta_{ij}}{N}$$

where *N* is the number of neurons *j* that node *i* projects to.

#### Structural balance

The combination of positive and negative edges in a network gives rise to both stable and unstable patterns of relationships ^49,97^. We determined the stability of the coactivity patterns in LM^sink^ and Rad^sink^ ripples by computing the structural balance of the neuronal graphs embedding the measured coactivity relationships (Figure S5E,F). We assessed structural balance by examining triads (three-node subgraphs), which are the smallest non-trivial motifs capable of expressing network consistency and are computationally efficient to analyse (i.e., the complexity of higher-order subgraphs increases combinatorially as *O*(*n*^*P*^) for P-node motifs). A triad is considered consistent (“balanced”) if it satisfies either of two conditions. First, all three nodes are positively connected (i.e., all edges are positive), indicating the absence of internal conflict (Figure S5E). Second, a triad is also balanced if two mutually positive nodes are both negatively associated to a third, a configuration often arising when a network is partitioned into two internally cohesive groups with antagonistic interactions (e.g., two coactive neurons while a third one is suppressed). By contrast, a triad is structurally inconsistent (“unbalanced”) if only one or all three edges are negative, as these sign configurations violate mutual consistency (Figure S5E). The greater the proportion of balanced triads in a network, the more structurally coherent its organisation (topology) of the relationships (here, coactivity) between its pairs of constituting nodes (here, neurons). In the context of neuronal networks, balanced triads typically reflect structured and internally consistent coactivation patterns, whereas unbalanced triads suggest more disorganised or stochastic coactivity. In Figure S5G,H, we computed the structural balance of the hippocampal graphs in Rad^sink^ versus LM^sink^ as the proportion of each graph’s triads which were balanced triads (Figure S5E,F).

#### Population-level sparsity

The sparsity (S) of a population firing vector (x) was computed using the Gini index ^98–100^ as:

$$S=\frac{\sum_{i=1}^{N}(2i-N-1)x_{i}}{N\sum_{i=1}^{N}x_{i}}$$

where *x* represents the population vector of spike counts for each principal cell, arranged in ascending order, within a 50-ms time window centred at the ripple peak (either Rad^sink^ or LM^sink^), *N* is the number of simultaneously recorded principal cells (i.e., the length of the vector), and *i* denotes the rank of spike count in ascending order. This ordering involved a fixed ranking based on the cluster indices assigned by the spike sorting algorithm (e.g., neuron 1, 2, …, N). Thus, the same ranking was used to compute the Gini index across all population vectors and all ripples recorded on the same day. Population vectors where spike counts are more evenly distributed across neurons have a lower Gini index (indicating lower sparsity), while those where spike counts are concentrated in a few neurons have a higher Gini index (indicating higher sparsity). In Figure 5D, we reported the sparsity for CA1 principal cells population vectors.

#### Population-level dimensionality

We quantified the intrinsic dimensionality of CA1 ripple-nested activity population vectors (i.e., within a 50-ms time window centred at the ripple peak) in Rad^sink^ versus LM^sink^ ripples using the angle based intrinsic dimensionality (ABID) measure ^46^. ABID is non-linear, making it suited for capturing complex neural activity patterns. Briefly, ABID estimates dimensionality (*D*) by analysing the cosine similarity between each ripple population vector (PV) and its k-nearest neighbours (k = 50). For a given sleep/rest session with *M* ripples (calculated separately for Rad^sink^ and LM^sink^), the ripple-nested PVs were z-scored, and the dimensionality of each ripple *m* was computed as:

$$D_{m}=\frac{k^{2}}{\sum_{i=1}^{k}\sum_{j=1}^{k}S_{ij}^{2}}$$

where *S_ij_* is the cosine similarity between the normalised k-nearest PVs of ripple *m*. Dimensionality is inversely related to the concentration of these similarities: when the cosine similarities (*S_ij_*) are high, indicating tightly clustered neighbours in the high-dimensional space, the sum of squared similarities $\left(\sum_{i=1}^{k}\sum_{j=1}^{k}S_{ij}^{2}\right)$ increases, resulting in lower dimensionality. Conversely, when similarities are lower, reflecting a more spread-out distribution of neighbours, the dimensionality is higher. The intrinsic dimensionality for each sleep/rest session was then computed as the average across all *M* ripples. To control for class imbalance between the two ripple types and across sleep/rest sessions, we randomly selected 100 PVs for each ripple type and calculated the intrinsic dimensionality of this subsampled data. This procedure was repeated 1,000 times, and for each sleep session, the mean intrinsic dimensionality for Rad^sink^ and LM^sink^ ripples was defined as the average across these 1,000 permutations (Figure 5E).

In Figure S5K, to control for the influence of population size on dimensionality estimates, we also computed the intrinsic dimensionality across random subpopulations of n = 5, 10, …, N CA1 principal cells for each session, using 1,000 permutations per subpopulation size. Moreover, to determine whether the differences in dimensionality between Rad^sink^ and LM^sink^ ripples reflect genuine coactivity structure rather than being purely driven by the sparsity differences (Figure 5D), we computed intrinsic dimensionality from surrogate datasets in which neuron identities were shuffled within each ripple. This procedure preserved the sparsity difference across ripple types while disrupting structured coactivation, thereby generating higher-dimensional population vectors (PVs) that represent unstructured, noisy activity. For each sleep session, the true intrinsic dimensionality of ripple events was then normalised by the corresponding value obtained from the surrogates, yielding a normalised dimensionality measure (Figure S5L) that reports how structured (i.e., low-dimensional) the true data are relative to a maximally uncorrelated version.

Finally, we cross-validated the intrinsic dimensionality findings by computing the linear dimensionality of Rad^sink^ and LM^sink^ ripples (Figure S5J). Linear dimensionality was estimated using the participation ratio, as described in Recanatesi et al. (2022) ^101^:

$$P=\frac{{\left(\sum_{i=0}^{N}\lambda_{i}\right)}^{2}}{\sum_{i=0}^{N}\lambda_{i}^{2}}$$

where *λ_i_* is the i^th^ singular value (sorted) and *N* denotes the number of cells recorded during that session. This metric quantifies how variance is distributed across principal components: high values indicate that variance is more evenly distributed, reflecting higher dimensionality; whereas low values indicate that variance is concentrated in fewer components, corresponding to lower dimensionality.

#### Controlling for individual firing rates in motif structure

To test whether the CA1 core motif structure during LM^sink^ ripples could be explained by differences in individual firing rates alone, we performed PCA on the corresponding z-scored LM^sink^ ripple–spike count matrix, where each row represented a neuron and each column a ripple (Figure S6A-C). The first principal component (PC1) captured the dominant pattern of joint activity; that is, the weighted combination of neurons that best explained shared variance in firing across LM^sink^ ripples. If motif structure was solely driven by individual firing rates, PC1 weights should be strongly correlated with each neuron’s average firing rate across ripples. We therefore computed the correlation between PC1 weights and each neuron’s mean firing rate across LM^sink^ ripples.

To assess whether any observed correlation could arise from firing-rate variability alone, we compared it to a null distribution obtained from surrogate LM^sink^ spike count matrices (see ‘Spikes Shuffling Control’). Importantly, these surrogates preserved the two main features of excitability-driven coactivation: each neuron overall firing rates and each ripple total spike count. This analysis was performed on sessions with at least 10 CA1 principal cells.

An illustration of this method is shown on simulated data in Figures S6A,B.

#### Coactivity motif extraction with Independent Component Analysis

We extracted coactivity motifs from population activity during LM^sink^ (or Rad^sink^) ripples using a previously published PCA/ICA-based assembly detection method ^47,48^. We first computed ripple-nested spike count of CA1 principal cells within a 50-ms time window centred at the ripple peak, thus obtaining a matrix of dimensions (*N_cells_* × *N_ripples_*). This activity matrix was then z-scored, resulting in matrix *Z*, where each neuron’s firing rate across events had zero mean and unitary variance. Principal component analysis was applied to *Z*, and we determined the number of significant motifs as significant the number of principal components with loadings above the Marčenko-Pastur distribution-based threshold $1+\sqrt{N_{cells}/N_{ripples}}$, which defines the upper bound for the concentration of variance expected from uncorrelated data. We then apply Independent Component Analysis to the *Z* projected onto the subspace spanned by these significant components to isolate meaningful coactivity patterns across ripples. We refer to the resulting components as LM^sink^ or Rad^sink^ motifs, depending on the ripple group analysed.

To test whether a LM^sink^ motif is embedded in the activity observed in Rad^sink^ ripples, we (1) quantified the cofiring of each CA1 principal cell with the LM^sink^ motif during Rad^sink^ and LM^sink^ ripples separately, and (2) tested whether there was a significant ‘contribution gain’ of that cell to the motif from LM^sink^ to Rad^sink^ ripples, accounting for differences in firing rate and sparsity; as detailed below.

To quantify the cofiring between a single neuron and a motif, we first computed the motif activation strength in each ripple as the inner product between the independent component unit vector (*v*) and the population activity in that ripple (i.e., a column in *Z*). To remove influence of the neuron being tested on the motif strength, we set the element of *v* corresponding to that neuron to zero before the computing the projection. We then calculated the correlation between the neuron’s activity and the motif activation across ripples. This correlation provided a measure of how strongly the neuron participated in the expression of the corresponding LM^sink^ motif during Rad^sink^ ripples. This procedure was repeated for all recorded principal cells.

We defined as the contribution gain of each neuron to each LM^sink^ motif as the difference between that neuron’s correlation to the motif strength obtained for LM^sink^ and Rad^sink^ ripples. To test if that contribution was significantly higher than expected given the increase in rate and lower sparsity in Rad^sink^ ripples, we compared the results to those obtained from surrogate activity matrices that preserved both the spike count of each neuron and the total spike count per ripple, while eliminating higher-order structure (see Section ‘Spikes Shuffling Control’).

An illustration of this approach is presented in Figure S6G.

This procedure was applied to each sleep session that included at least 10 CA1 principal cells. PCA and ICA (using the FastICA algorithm) were implemented via the scikit-learn library.

In Figure 5G, we report these mean changes across all cells for each motif. Moreover, for each sleep session, we computed the number of neurons that got ‘aggregated’ relative to what was expected from the surrogate distributions across all detected motifs (Figure S6I). As an additional control, we repeated the analysis in the opposite direction: extracting Rad^sink^ motifs and evaluating changes in individual neuron participation during LM^sink^ ripples (Figure S6H). Finally, in Figure 6D, we tested whether the aggregated neurons were more likely to correspond to superficial CA1 pyramidal cells by comparing these z-scored recruitment changes.

#### Cross-population vector inclusion of activity motifs

To quantify the degree of inclusion between the coactivity patterns in LM^sink^ versus those in Rad^sink^ ripples, we computed the overlap among their population vectors (PVs). Each ripple-nested PV contained the instantaneous firing activity of CA1 principal cells within a 50-ms window centred around the ripple peak. Each PV was then described as a binary vector where the presence of any spikes from a neuron is denoted by 1, and absence of any spike from a neuron is denoted by 0. This approach allowed constructing an adjacency matrix, where each entry provides a measure of overlap between the activity patterns of different PVs (see Figure S6D-E). Given two population vectors, *PV_m_* = [*p_m1_*, p_*m*2_, …, *p_mn_*] and *PV_q_* = [p_*q*1_, p_*q*2_, …, *p_qn_*], where *n* represents the total number of principal cells in the population, and *p_mk_, p_qk_* indicate the (binary) firing state of the *k* -th neuron in the pair of ripple-nested population vectors *m* and *q*, respectively. To quantify the overlap in active neurons between two PVs, we computed an asymmetric version of the Jaccard coefficient between *PV_m_* and *PV_q_*. Effectively, this metric, denoted as *I_m,q_*, quantifies the proportion of cells in *PV_m_* that are also active in *PV_q_*:

$$I_{m,q}=\frac{\left|PV_{m}\cap PV_{q}\right|}{\left|PV_{m}\right|}$$

where the ∩ symbol represents the intersection between the two sets of active cells, and the | · | operator represents the cardinality of the set, i.e. the number of (active) cells it contains. Thus, here a value of 1 indicated that all cells active in *PV_m_* were also active in *PV_q_*, and zero if none were active in both. Similarly, we defined the proportion of cells in *PV_q_* that are also active in *PV_m_*:

$$I_{q,m}=\frac{\left|PV_{m}\cap PV_{q}\right|}{\left|PV_{q}\right|}$$

Note the different denominator with respect to the previous equation. It is important to also note that this operation is inherently asymmetric due to the directional nature of the cardinality of the two sets, resulting in *I_m,q_* not necessarily being equal to *I_q,m_*. This asymmetry allowed exploring directional similarities in activity patterns and thus provided insights into the pattern similarities between PVs. To highlight the importance of this directionality (e.g., in Figure S6D-F), we employed notation *I_m,q_* = I_*m*→*q*_.

#### Neural inclusion of LM^sink^ into Rad^sink^ ripples

For each sleep session, we computed the inclusion between each pair (*m, q*) of ripple-nested PVs (as presented in the section above). Using pairs of PVs nested in LM^sink^ and Rad^sink^ ripples, we quantified the degree of active cells similarity in the sets of active neurons from LM^sink^ into Rad^sink^ ripples (see Figure S6F). This provided insights into the directional pattern similarities and differences between these ripple types. To account for the potential bias due to an imbalance in the number of instances between LM^sink^ and Rad^sink^ ripples, we standardized the comparison by matching the number of PVs from each class to the smaller of the two, denoted by *D*. Then, the mean overlap of LM^sink^ into Rad^sink^ PVs (I_*LM*→*Rad*_) was calculated as the average across these subsampled *D* PVs.

To further mitigate potential biases arising from subsampling specific sets of PVs, we adopted a resampling strategy. In each iteration, the inclusion *I*_LM→Rad_ was recalculated using a randomly selected subset of *D* PVs from the class with the larger number of instances. This procedure was repeated across 1,000 iterations to account for variability within the data. It is important to note that we avoided direct comparison between *I*_LM→Rad_ and I_*Rad*→*LM*_ because the sparsity differences between the two ripple sets could bias their respective overlaps.

#### Classification of CA1 principal cells into deep and superficial

We classified CA1 principal cells as either deep or superficial, following the approach described in previous work ^37^. Briefly, we extracted LFP features from the tetrode where each principal cell was recorded. These features were then projected onto a linearized trajectory, which estimates the cell’s depth within the pyramidal layer specifically, whether the associated tetrode is closer to the *stratum radiatum* or the *stratum oriens*. As illustrated in Figure 6A, the depth distribution of CA1 principal cells along this trajectory is bimodal. Using this distribution, we classified all recorded cells either as superficial or deep, with a threshold depth value of 6 separating the two groups, resulting in a total of 1,353 deep and 843 superficial cells. For analyses where the criteria described in ‘Principal cell versus interneuron classification’ were applied, the numbers were refined to 1,100 deep and 480 superficial cells. To validate that this method captures the expected characteristics of deep and superficial cells ^37,57,66,67^, we report in Figure S6J,K their mean firing rates during theta oscillations and ripple events.

#### Change in firing rate during ripples relative to baseline

In Figures 6C, we compared the change in firing rate deep and superficial principal cells relative to a pre-ripple baseline. This allowed comparing cells with different firing rates in ripples (i.e., deep versus superficial CA1 principal cells. To compute this measure, we calculated the mean instantaneous firing activity of each cell within a 50-ms window centred around the ripple peak (rate_ripple_). To estimate the baseline firing rate (rate_baseline_), we computed the mean firing activity in a time window from −200 ms to −100 ms relative to the ripple peak. Then, independently for Rad^sink^ and LM^sink^ ripples, we calculated the increase in firing rate as:

$$\text{Δ}\;\text{rate}=\log_{10}\left(\frac{{\text{rate}}_{\text{ripple}}}{{\text{rate}}_{\text{baseline}}}\right)$$

where positive values of Δrate indicate an increase in firing rate relative to the pre-ripple baseline, while negative values indicate a decrease. For this analysis, we included only isolated ripples to avoid contamination of the baseline rate (see section ‘Sharp wave-ripple (SWR) event detection’).

#### Additional cells active in Rad^sink^ but not in LM^sink^ ripples

In the analysis presented in Figure S6L, we investigated cells exhibiting preferential firing for Rad^sink^ but not LM^sink^ ripples (see Figure 5 and S6D-F). We first identified the cells active in each ripple, as described in section “Cross-population vector inclusion of activity motifs.” For each cell, we then calculated the conditional probability of it being active in a Rad^sink^ ripple given that it was inactive in an LM^sink^ ripple. To control for the intrinsic firing probability of each cell across ripples, we estimated a chance level for each cell using a shuffling procedure. This procedure independently shuffled the coactive neurons during Rad^sink^ and LM^sink^ ripples while maintaining the total number of ripples in which the cell was active and preserving the sparsity of each ripple (see “Spikes shuffling control” section). The shuffling process was repeated 200 times, and the resulting chance levels were used to z-score the observed conditional probabilities.

#### Offline reactivation

To analyse offline reactivation of CA1 or CA3 (Figure S7A,B) principal cell spiking patterns during sleep/rest, we first trained a linear regression model using awake theta-cycle activity from the exploration session of that recording day. Specifically, the model was trained to predict the firing rate of a target cell based on the activity of four other cells, ensuring that none of the predictor cells were recorded on the same tetrode as the target cell. This criterion allowed avoiding biases in correlations that could arise from shared global tetrode activity. Model accuracy was evaluated as the Pearson correlation between the actual firing rate of the target cell and the firing rate predicted by the model. To ensure robustness, we performed 100 bootstrap iterations, randomly selecting four predictor cells from the pool of cells recorded on the same day for each iteration. Next, we applied these cross-validated models to the z-scored ripple-nested (using a 50-ms window centred around the ripple peak) firing activity of the target cell during pre-exploration and post-exploration sleep/rest sessions. For each cell, the mean accuracy across the 100 cross-validated models was calculated separately for pre-exploration sleep and post-exploration sleep, providing two model accuracy values. Reactivation was defined by comparing the overall accuracy across all cells in post-exploration sleep to the corresponding accuracy in pre-exploration sleep. Specifically, significant reactivation was identified if the post-exploration accuracy was higher than the pre-sleep accuracy (1-tailed paired bootstrap tests). This analysis was performed independently for Rad^sink^ and LM^sink^ ripples during both pre- and post-sleep.

#### Deep and superficial cells reactivation

In Figure 6E-G, we computed the offline reactivation of awake spiking patterns for deep and superficial cells. This analysis followed the same approach described in the “Offline reactivation” section, with an additional criterion: predictor cells and the predicted cell were required to belong to the same CA1 pyramidal sublayer. Specifically, a set of four superficial cells was used to predict the activity of another superficial cell, and a set of four deep cells was used to predict the activity of another deep cell, as illustrated in Figure 6E. Together with the existing criterion that predictor cells and the predicted cell must be recorded on different tetrodes (see section “Offline reactivation”).

#### Recent-to-prior coactivity motif balance

We analysed whether the coactivity patterns observed in Rad^sink^ and LM^sink^ ripples were more aligned with recent wakefulness (i.e., theta coactivity patterns during open-field exploration) or with pre-existing patterns (i.e., ripple coactivity patterns during baseline sleep immediately before exploration). For this, we constructed coactivity matrices (Population-conditioned coactivity described in section “Neuronal coactivity graphs”) for each period: pre-sleep, awake, and post-sleep. As before, coactivities during the awake session were computed across theta cycles, while ripple events were used for the sleep sessions. For pre-sleep, we sampled an equal number of Rad^sink^ and LM^sink^ ripples, establishing a coactivity baseline.

To isolate coactivity motifs expressed during wakefulness but absent in pre-sleep, we modelled the change in coactivity from pre-sleep to wakefulness as a linear transformation:

$$A_{theta}=W_{recent}*A_{pre}$$

From this, we computed the transformation matrix:

$$W_{recent}=A_{theta}A_{pre}^{-1}$$

where *W_recent_* represented the coactivity motifs strengthened during wakefulness relative to pre-sleep. The *A*^−1^ matrix can be interpreted as a whitening step, effectively decorrelating the coactivity patterns observed during pre-sleep. Thus, $A_{theta}A_{pre}^{-1}$ captures the structure that emerges in awake after removing baseline patterns. We then computed, for each ripple:

$$recent-to-priorbalance=v^{T}W_{recent}v$$

where *v* is the population vector in a ripple in post-sleep. This measure reflects how strongly the ripple’s coactivity motifs align with recent (wake-specific) versus prior (pre-sleep) motifs. Intuitively, components of *v* that align with pre-sleep patterns (*A_pre_*) are down-weighted by the transformation, while components aligning with wake patterns (*A_theta_*) are emphasized. As a result, *v*^*T*^*W_recent_v* increases when the ripple coactivity pattern resembles motifs gained during wakefulness (relative to pre-sleep) and decreases when it resembles pre-sleep patterns. Similarly, one can also define the transformation $W_{prior}=A_{pre}*A_{theta}^{-1}$ which captures the coactivity motifs expressed in pre-sleep but not present in wakefulness.

Balance scores were computed separately for Rad^sink^ and LM^sink^ ripples during post-sleep. Within each sleep session (or time window), these values were averaged across ripples to yield the balance score for each ripple type.

In Figure S7G, we applied the same framework described above to assess CA3–CA1 coactivity motifs. Coactivity matrices were constructed as before but including all CA1 and CA3 principal cells. To isolate the contribution of CA3–CA1 pairs, all coactivity matrix entries were set to zero except those corresponding to pairs formed by one CA3 and one CA1 principal cell. Note that the CA1-only reactivation described above corresponds to retaining only CA1–CA1 entries and zeroing all others. These CA3-CA1 coactivity matrices were then used to project population vectors from both Rad^sink^ and LM^sink^ ripples, as before, but using activity from both CA1 and CA3 neurons.

An illustration of this method is shown through simulations in Figures S7C-F.

#### Recent-to-prior coactivity motif balance across CA1 sublayers

In Figure 7C, we measured the recent-to-prior motif balance separately within either deep or superficial CA1 principal cells. Specifically, we computed the post-sleep balance score (see ‘Recent-to-prior coactivity motif balance’) using groups of five randomly sampled deep cells or five superficial. For each session with more than five cells in a sublayer, we performed up to 500 permutations by randomly sampling five cells from the group and obtained the average across permutations. Recordings with fewer than 5 cells in either group were excluded from this analysis. Finally, each recording day we compared the balance scores between deep and superficial cells.

#### Resolving recent-to-prior coactivity motif balance across time

In Figures 7D and S7H-J, we quantified changes in the recent-to-prior motif balance (see ‘Recent-to-prior coactivity motif balance’) across post-sleep. For this analysis, we included recordings in which pre- and post-sleep epochs lasted at least 1 hour. Balance scores were computed as before, but within non-overlapping 10-minute windows spanning the first post-sleep hour. To ensure comparability across recordings with different numbers of recorded cells, scores were normalised within each recording by subtracting the global mean across all time bins and ripple types (Rad^sink^ or LM^sink^). To test for significant changes over time (as opposed to scores remaining constant), we fit the time course of each ripple type using an exponential model (see “Exponential fit of motifs dynamics throughout sleep” section).

#### Exponential fit of motifs dynamics throughout sleep

To determine whether motif expression changed over post-exploration sleep, we compared two models: a constant model representing the mean and an exponential decay model (see equation below). For each analysis (e.g., recent-to-prior balance, pre-sleep coactivity strength and wakefulness reactivation), we concatenated data across all sleep/rest sessions, creating two vectors: one containing the time point of each bin and the other containing the corresponding motifs balance (or reactivation) values.

We then fit both models and computed the Bayesian Information Criterion (BIC) to assess which model better explained the observed motif expression trends. BIC penalises model complexity, thereby preventing overfitting when comparing models of differing order:

$$BIC=k*\log(n)+n*\log\left(\frac{\sum_{i=0}^{n}{\left(y-y_{pred}\right)}^{2}}{n}\right)$$

where *k* is the model order (k = 1 for the flat line, and k = 3 for the exponential model), *n* is the number of samples, and $\sum_{i=0}^{n}{\left(y-y_{pred}\right)}^{2}$ is the sum of squared errors between the true motif dynamics and the values predicted by each model (flat or exponential). If the BIC of the exponential model was lower than that of the flat model, the dynamics were considered to follow an exponential trend and was further investigated as described below.

Dynamics changing exponentially were defined as:

$$motifstrength(t)=a\cdot e^{-\frac{t}{\tau }}+c$$

where *a* is the amplitude (scaling factor), *τ* is the time constant (decay parameter), and *c* is the offset or baseline.

A hyperparameter search was conducted to find the best initial estimates for *a*, *τ*, and *c*, maximising the fit between the model and the data. The goodness of fit was quantified using the Pearson correlation *r* between the observed motif strength values and the mode predictions. To estimate the mean fit score (*r*) and time constant (*τ*), we conducted a cross-validation by fitting the exponential function using a bootstrapped set of recording days. This procedure was repeated over 10,000 resamples, generating distributions of estimates for the *r* and exponential function parameters (i.e. *a*, *τ* and *c*), which were used to plot the model fit (see Figures 7E-F). Importantly, to control for early sleep stages transitions to bias estimates of trend over time, the initial 10 minutes of sleep were excluded from these analyses, as those epochs might be associated with a higher probability of rest-to-sleep transitions.

#### Recent-to-prior motif balance over time controlling for ripple occurrence frequency and ripple population sparsity

In Figure S7H-J, we evaluated whether the observed gradual changes in recent-to-prior motif balance over time during LM^sink^ ripples (see Figure 7D) were genuinely time-dependent (i.e., related to the ripple occurrence time in sleep/rest session) or driven rather related to changes in ripple occurrence frequency (i.e., how many ripples per unit of time) or ripple population sparsity (i.e., how many active neurons per ripple) (Figure S7H). To address this, we concatenated the time-binned recent-to-prior motif balance (during Rad^sink^ or LM^sink^ ripples) and trained a linear regression model to these values at each time bin (see section ‘Resolving recent-to-prior coactivity motif balance across time’). The predictors included: (1) the ripple occurrence time (i.e., the time of the bin), (2) the ripple occurrence frequency (mean number of ripples per minute in each time-bin), and (3) the ripple population sparsity (calculated as the mean proportion of active cells in ripples in each time-bin). Given the non-linear relationship between time and motifs balance (see Figure 7D), we applied a log transformation to the ripple occurrence times to linearise this relationship. To avoid potential biases introduced by the changes in behavioural states, we excluded the first 10 minutes from each session. The model was trained and cross-validated 20 times (80% training, 20% testing), with accuracy measured as the Pearson correlation between the observed recent-to-prior motif balance and the model’s predictions. To establish a chance level, we shuffled the recent-to-prior motif balance values 25,000 times and compared the model’s accuracy to these shuffled data (Figure S7I). To determine the contribution of each feature to the model’s accuracy, we trained additional models where individual features were shuffled 25,000 times, thereby removing specific information from the shuffled feature. The gain in accuracy for a given feature was calculated as the difference between the accuracy of the original model and that of the feature-shuffled model (Figure S7J). A high accuracy gain for a feature indicated strong predictive power, showing that the feature contributed significantly to changes in motifs balance. The significance of each feature was assessed by calculating p-values as the proportion of shuffled models with a gain less than 0 (indicating the feature significantly contributed to the model).

#### Reactivation strength of pre-sleep coactivity and dynamics in LM^sink^ and Rad^sink^ ripples

To measure whether the coactivity patterns nested in Rad^sink^ and LM^sink^ ripples changed from pre-exploration to post-exploration, we first isolated ripple-nested population vectors (PVs) that contained the instantaneous firing activity of CA1 principal cells within a 50-ms window centred around the ripple peak. This was done separately for pre-exploration sleep and post-exploration sleep/rest sessions. Using the z-scored PVs from pre-sleep, we trained a generalized linear model (GLM) to predict the firing response of each CA1 principal cell based on the activity of the rest of the population during each ripple. This process was repeated independently for Rad^sink^ and LM^sink^ ripples, yielding two models for each cell (one for LM^sink^ and one for Rad^sink^ ripples). Each model was cross-validated 20 times (80% training, 20% testing), and accuracy was assessed as the mean correlation between the predicted and true activity of the testing set for each iteration: *r_pre,i_*. To estimate the chance level for each GLM, we used a shuffling procedure that randomized the cell IDs within each

PV 500 times prior to training, breaking cell correlations but preserving the overall rate for each PV. To measure the overall coactivity changes from pre-to post-exploration rest, we applied the cross-validated model of each cell to the (z-scored) PVs from post-sleep and measured its accuracy (*r_post,i_*). For both Rad^sink^ and LM^sink^ ripples, we quantified the coactivity stability for cell *i* as:

$$Stability_{i}=1-\left(r_{pre,i}-r_{post,i}\right)$$

where *r_pre,i_* and *r_post,i_* are the accuracy values in pre- and post-sleep of the GLM of cell *i* which were normalised with respect to the chance level estimated through the shuffling procedure mentioned above (Figure S7K). A high stability score (~1) indicates that the model’s accuracy was similar between pre- and post-sleep, reporting that coactivity patterns did not undergo significant cross-session changes. Conversely, a low stability score reflects a substantial change in coactivity from pre-to post-sleep.

In Figure 7E, we adapted a similar framework to quantify the reactivation strength of pre-exploration sleep ripple coactivity throughout the time in post-exploration sleep ripples. Specifically, we tested whether the recent-to-prior motifs balance trends observed in Figure 7D reflected a disengagement from prior motifs, by assessing whether the expression of pre-exploration sleep coactivity in LM^sink^ ripples declined over the course of post-exploration sleep (“post-sleep”) lasting at least one hour. To do so, as described above, for each CA1 principal cell, we fit a linear model using the activity of all other cells during pre-exploration sleep ripples (including only pre-sleep with at least 250 Rad^sink^ and 250 LM^sink^ ripples). We then applied these models to predict activity during ripples occurring in successive, non-overlapping 10-minute windows of post-sleep. The average prediction accuracy across all neurons, in each post-exploration sleep window was taken as the reactivation strength of pre-exploration sleep coactivity. The observed dynamics of these pre-sleep coactivities were used to fit an exponential function whose predictions were used to generate the data shown in Figure 7E (see section “Exponential fit of motifs dynamics throughout sleep”).

#### Dynamics of sublayer-specific pyramidal reactivation of pre-sleep

In Figure S7L-M, we assessed whether the decay observed throughout post-exploration sleep in the reactivation strength of pre-exploration sleep coactivity during LM^sink^ ripples differed between deep and superficial CA1 principal cells. To do this, we repeated the analysis described in the section “Reactivation strength of pre-sleep coactivity and dynamics in LM^sink^ and Rad^sink^ ripples” separately for deep and superficial cells (e.g., predicting each deep cell’s activity using the other deep cells, and analogously for superficial cells), with no averaging across all cells. This allowed us to estimate the temporal decay of reactivation of pre-sleep coactivity during post-sleep LM^sink^ ripples separately for the two pyramidal sublayers.

To evaluate whether deep and superficial cells disengaged from prior motifs at similar or different speeds, we trained linear regression models to predict the reactivation strength of pre-sleep coactivity from time (log-transformed to account for exponential trends). The beta coefficient of each model represented the rate of decay, i.e., the slope of reactivation strength weakening over time (with negative values indicating a decline). To obtain robust estimates of this rate, we trained the models using data concatenated across a bootstrapped subset of sleep sessions (10,000 iterations), separately for deep and superficial cells. To assess statistical significance, in each bootstrap iteration we generated a chance-level beta by circularly shuffling the time labels. This preserved the autocorrelation structure of the time series while destroying the relationship between time and motif strength. In Figure S7L, we report the z-scored beta values of the true model relative to this surrogate distribution, separately for deep and superficial cells.

Using a similar framework, we also asked whether the LM^sink^ recent-to-prior motif balance trends observed in Figure 7D could be better explained by the decay of reactivation of pre-sleep coactivity in deep or superficial cells. To do so, we repeated the same regression procedure as above, but used the mean LM^sink^ motifs balance (rather than time) to predict the reactivation strength of pre-sleep coactivity. In this case, the beta coefficient reflected the correlation between LM^sink^ recent-to-prior motifs balance trends and the layer-specific expression of pre-sleep coactivity patterns across post-sleep windows.

#### Reactivation strength of awake coactivity and dynamics in LM^sink^ and Rad^sink^ ripples

To test whether the drift in expressed motifs observed in Figure 7D reflected an increase in the expression of recent motifs, we examined whether the reactivation of exploration-related coactivity patterns changed over the course of post-exploration sleep. To do this, we first extracted CA1 principal cells coactivity graphs from pre-exploration sleep, exploration, and post-exploration sleep sessions, as described in the section “Recent-to-prior coactivity motif balance”. We then trained linear regression models to estimate pairwise cofiring correlations between the awake and post-sleep periods, while regressing out pre-sleep cofiring. The resulting beta coefficients quantified the degree to which waking coactivity patterns were re-expressed during post-sleep ripples not explained by pre-sleep, i.e., the reactivation strength. This analysis was performed on ripple events grouped into successive, non-overlapping 10-minute windows across the post-sleep period, allowing us to track the temporal dynamics of awake coactivity reactivation. These reactivation values were then used to fit an exponential function, whose predictions generated the data shown in Figure 7F (see section “Exponential fit of motif dynamics throughout sleep”).

### Quantification And Statistical Analysis

Data analyses were conducted using Python versions 3.6 and 3.10, incorporating the following packages: DABEST ^103^, scikit-learn ^104^, NetworkX ^105^, NumPy ^106^, SciPy ^107^, Stats-Models ^108^,Matplotlib ^109^, Pandas ^110^, and Seaborn ^111^. Symmetric distribution assumptions underpinned the two-sided statistical tests, visualised using Gardner-Altman and Cumming plots from the DABEST framework. These plots illustrate effect sizes by comparing mean or median differences across groups. Each plot consists of two panels: the top shows raw data distributions with group means ± SEM (unless stated otherwise), and the bottom shows differences relative to a reference group, calculated from 5,000 bootstrapped samples. Black dots represent the mean, black ticks indicate 95% confidence intervals, and bootstrapped error distribution curves are included. Statistical comparisons included t-tests against a mean (to compare one distribution to a fixed value) and one-way ANOVA for multiple conditions, followed by a Tukey post-hoc test. To compare two conditions, bootstrap tests were employed. These tests, which accommodated both paired and unpaired comparisons, estimated the bootstrapped mean difference (either absolute or as a percentage relative to one of the two variables) by resampling the data 100,000 times (unless stated otherwise) with replacement. For paired comparisons, indices were resampled to preserve the relationship between pairs, whereas for unpaired comparisons, each condition was resampled independently. P-values for these tests were computed numerically, under the null hypothesis of zero difference. For one-sided tests, the p-value was calculated as the proportion of bootstraps where the difference distribution was either greater than or less than zero, depending on the test direction. For two-sided tests, the p-value was determined by multiplying the smaller proportion of bootstraps below or above zero by two. Unless otherwise specified, all p-values from bootstrap tests were two-sided. In some instances (e.g., Figures 5B, 6F-G, 7B-C), we visualised the bootstrapped mean differences using histograms, from which the corresponding p-values were derived. These histograms do not depict the distribution of raw data points but rather represent empirical estimations of the sampling distribution of the mean difference, obtained by the resampling approach described above. All confidence intervals (95% CI) were calculated via bootstrapping with 100,000 resamples (unless stated otherwise). For each interval, data were resampled randomly with replacement, and the 2.5^th^ and 97.5^th^ percentiles of the bootstrapped distributions determined the lower and upper bounds of the CI.

## Supplementary Material

Supplemental Figures and Tables

## Data Availability

The electrophysiology dataset reported in this study is being used in ongoing projects and can be accessed under a data transfer agreement. We welcome enquiries for sharing it; please contact david.dupret@bndu.ox.ac.uk. The LDA-based classifier for hippocampal ripple events has been deposited on GitHub and archived on Zenodo (https://doi.org/10.5281/zenodo.16995137) and is publicly available. Any additional information required to reanalyze the data reported in this paper is available from the lead contact upon request.
