# Supplementary material for: Hippocampal Ripple Diversity organises Neuronal Reactivation Dynamics in the Offline Brain
Source: Neuron. Author manuscript; Available in PMC 2025 Oct 10. (PMC7618225; doi:10.1016/j.neuron.2025.09.012)
Supplement: Supplemental Figures and Tables [file EMS208753-supplement-Supplemental_Figures_and_Tables.pdf]

Hippocampal Ripple Diversity organises Neuronal Reactivation Dynamics in the Offline Brain

Manfredi Castelli[1,#,*], Vítor Lopes-dos-Santos[1,#,*], Giuseppe P. Gava[1], Renaud Lambiotte[2], David Dupret[1,3,*]

[1] Medical Research Council Brain Network Dynamics Unit, Nuffield Department of Clinical Neurosciences, University of Oxford, UK

[2] Mathematical Institute, University of Oxford, UK

[3] Lead Contact

#These authors contributed equally.
*Correspondence: manfredi.castelli@ndcn.ox.ac.uk (M.C.),
vitor.lopesdossantos@ndcn.ox.ac.uk (V.L.-d.-S.), and david.dupret@bndu.ox.ac.uk (D.D.)

Supplementary Figures and Tables

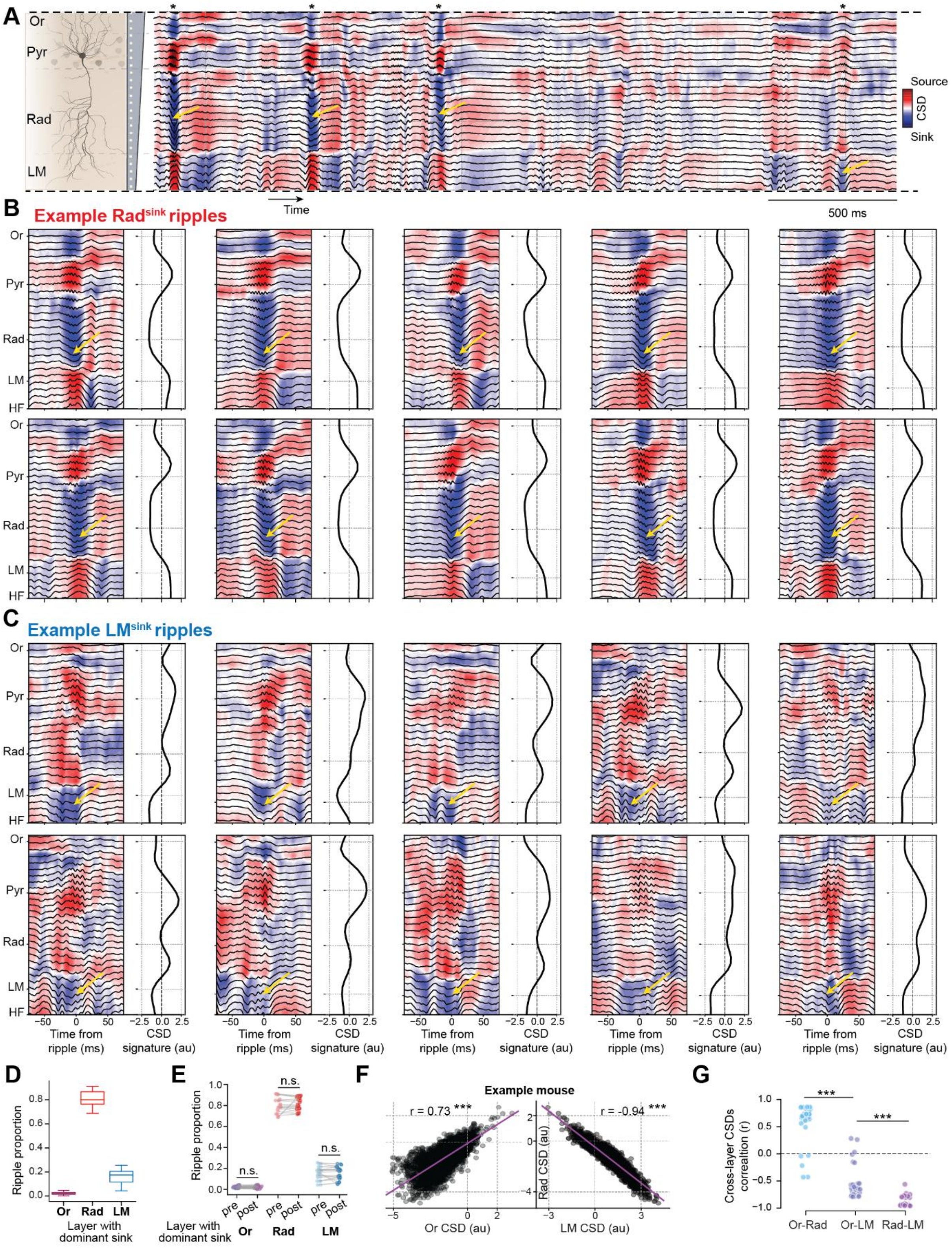

Figure S1: Individual Rad^{sink} and LM^{sink} ripples have distinct CSD profiles, related to Figure 1.

(A) Example silicon probe recording (a 3-sec sample for clarity) spanning the somato-dendritic axis of CA1 principal cells to show the instantaneous CSD (coloured hit map; red, source; blue, sink) and local field potential waveform (black traces) across the different neural layers (*strata oriens*, Or; *pyramidale*, Pyr; *radiatum*, Rad; *lacunosum-moleculare*,

LM) during a sleep session. Each black asterisk indicates an individual ripple, and each yellow arrow marks a current sink (in *stratum radiatum* or *stratum lacunosum-moleculare*).
(B-C) Shown are raw CSD signals (*left*, hit maps) with LFP waveforms (*left*, black traces), and the associated CSD signature (*right*, black curve) for ten Rad^{sink} ripples (B) and ten LM^{sink} ripples (C). Yellow arrows highlight a current sink in stratum radiatum or stratum lacunosum-moleculare, as appropriate.
(D) Box plots showing the proportion of ripples with a dominant current sink in oriens (Or), radiatum (Rad), and lacunosum-moleculare (LM), computed per sleep/rest session.
(E) Scatter plots showing the proportion of ripples with a dominant sink in oriens (Or), radiatum (Rad), and lacunosum-moleculare (LM) in pre-exploration (pre) and post-exploration (post) sleep. None of the different profiles significantly changed their proportion from pre- to post-sleep (each pair of dots represents a pair of pre and post sleep sessions; all Ps > 0.35; paired bootstrap test; n = 23 pairs of pre- and post-sleep from five mice).
(F) Relationship between individual ripple-CSD values in *stratum oriens, radiatum, and lacunosum-moleculare*. Each dot represents a ripple, with coordinates indicating the average CSD values across the three layers for an example sleep session. Purple lines represent the best linear fit; numbers report the Pearson correlation coefficients of ripple-CSDs across two layers.
(G) Distribution of mean Pearson correlation coefficients of ripple-CSDs across different layers. Each dot corresponds to a sleep session ($p = 5x10^{-48}$; one-way ANOVA; all pairwise $Ps < 10^{-6}$, Tukey post-hoc test).
*p < 0.05, **p < 0.01, ***p < 0.001. (D and G) shown data are from n = 38 sleep/rest sessions from five mice.

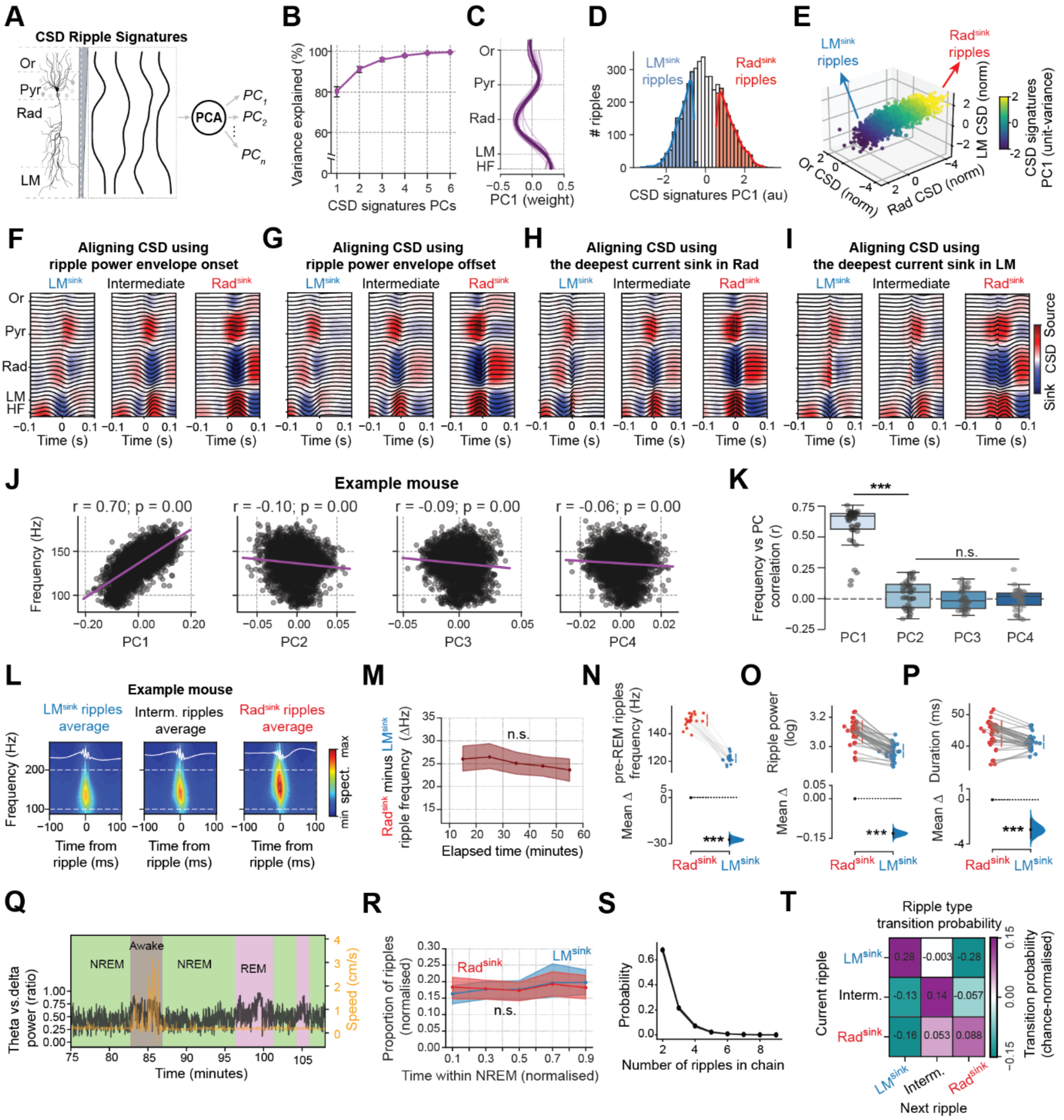

Figure S2: Rad^{sink} and LM^{sink} ripples can be distinguished by Principal Component Analysis of laminar CSD profiles, related to Figure 1.

(A) We applied Principal Component Analysis (PCA) on the CSD signatures (spanning the CA1 radial axis) of all individual ripples, as a dimensionality reduction method to identify CSD-signature components that explained the most variance across individual ripple events.

(B) Mean variance explained by each CSD signature principal component (PC) [mean variance explained of PC1 (95% CI): 80.3 (77.6 –82.6) %]. *Black error bars*, 95% CI.

(C) Laminar organisation of PC1 weights. Note the larger contributions from sinks in stratum radiatum, which align with the averaged CSD profile reported in Figure 1B. *Thinner lines*, individual sleep/rest sessions. *Thicker line*, mean PC1 across all sessions.

(D) Distribution of PC1 strength for all ripples in an example sleep session. Ripples at the lower and upper extremes of the distribution (bottom and top 30%) correspond to stronger current sinks in lacunosum moleculare (LM^{sink} ripples, blue) and radiatum (Rad^{sink} ripples, red), respectively (see Figure 1C).

(E) 3D scatter plot of ripples from sleep sessions in one example mouse, with coordinates representing instantaneous ripple-CSD values in stratum oriens, radiatum, and lacunosum-moleculare. CSD values normalised to be unit-variance. Points are color-coded by PC1 strength, highlighting the continuum of ripples in this lower-dimensional space. Ripples with stronger PC1 values align with the two profiles identified in (D), corresponding to Rad^{sink} or LM^{sink} ripples.
(F-I) By aligning CSD with different timestamps within the ripple event, we observed that the Rad^{sink} and LM^{sink} profiles consistently emerged regardless of how the LFP and the CSD signals were referenced. Shown are triggered average CSD and LFP signals across CA1 layers during LM^{sink}, Intermediate, and Rad^{sink} ripples (see methods) from one example mouse. Note that Rad^{sink} and LM^{sink} ripple profiles were consistent regardless of whether LFP and CSD signals were triggered using the ripple onset (F), offset (G), or ripple current sinks in the radiatum (H) or lacunosum-moleculare (I) layers. To avoid contamination of the averages by overlapping events, this analysis only includes ripples that were isolated (i.e., with no other ripple within ± 250 ms).
(J) Scatter plots showing the relationships between ripple frequency and CSD principal component (PC) strength for an example sleep session. Each point represents a ripple. Pearson correlation values computed between ripple frequency and the strength of each PC. Only PC1 showed a strong correlation with ripple frequency.
(K) Box plot and scatter plot showing the mean Pearson correlation between ripple frequency and strength of each PC. Each dot represents a sleep session. Note that the stronger PC1 strength, indicative of a stronger sink in the radiatum, is associated with higher ripple frequency ($p = 1x10^{-57}$, one-way ANOVA; PC1 versus PC2: $p = 6.9x10^{-14}$, Tukey post hoc test).
(L) Spectrograms of CA1 pyramidal LFPs from one example mouse across ripple types. The panels (from left to right) show the average spectrogram (with LFP waveform, white trace) for LM^{sink}, intermediate (interm.), and Rad^{sink} ripples within a ±100 ms window centred on the ripple-envelope peak. Note that from left to right the average ripple power increases as well as the mean ripple frequency.
(M) Mean ripple frequency difference between Rad^{sink} and LM^{sink} ripples, over time across sleep sessions. Note that this trend was constant, suggesting that the differential ripple frequency was not driven by changes in sleep/rest stages ($p = 8.5x10^{-2}$; bootstrap test; n = 28 sleep/rest sessions from five mice).
(N-P) Estimation plot showing the effect size for the difference in ripple frequency (N), ripple power (O), and ripple duration (P). Note that the difference in ripple frequency in (N) was computed for ripples preceding REM sleep, i.e. sleep epochs unlikely to correspond to rest-to-sleep transitions. This is in line with the results in Figure 1E (n = 20 sleep sessions with at least one REM bout detected from three mice). These results also show that the ripple power (O) and duration (P) are significantly higher in Rad^{sink} than LM^{sink} ripples (all $Ps < 10^{-5}$; bootstrap test). Each dot represents a sleep session.
(Q) Example ~30-minute window from one sleep session showing theta-power ratio and tracking speed. Theta-power ratio was computed as theta-range power (5-10 Hz) normalised to the total power in 0.1-10Hz. Together with tracking speed, this metric was used to distinguish NREM, REM, and awake epochs.
(R) Mean temporal distribution of Rad^{sink} and LM^{sink} ripples within NREM sleep epochs. For each NREM epoch, time was warped, and the proportion of ripples occurring in each warped-time bin was computed separately for Rad^{sink} and LM^{sink} events. No significant temporal changes were observed for either ripple type (all Ps > 0.35; bootstrap test; n = 22 sleep sessions from three mice; see Section 'Occurrence distribution of Rad^{sink} and LM^{sink} ripples in NREM sleep').

(S,T) To examine whether Rad^{sink}, intermediate, and LM^{sink} ripples occur interchangeably or cluster in bursts, we extracted chains of ripples and modelled ripple type as a first order Markov process (see Methods). (S) is the probability distribution of the number of ripples within a chain (*shaded area*, 95%CI). (T) is the ripple-type transition matrix showing the probability of a ripple of a given type (vertical axis) following a ripple of another type (horizontal axis) in ripple chains. Transitions probabilities were normalised relative to chance by subtracting the mean values from surrogate sequences obtained by shuffling the ripple-type labels. Note that within-ripple type transitions (e.g., $LM^{sink} \rightarrow LM^{sink}$) occurred significantly more often than expected by chance whereas cross-ripple type transitions (e.g., $LM^{sink} \rightarrow Rad^{sink}$) were significantly less frequent than chance.
$^{*}p < 0.05$, $^{**}p < 0.01$, $^{***}p < 0.001$. (B-C,K,O-P and S-T) data shown are from n = 38 sleep/rest sessions from 5 mice.

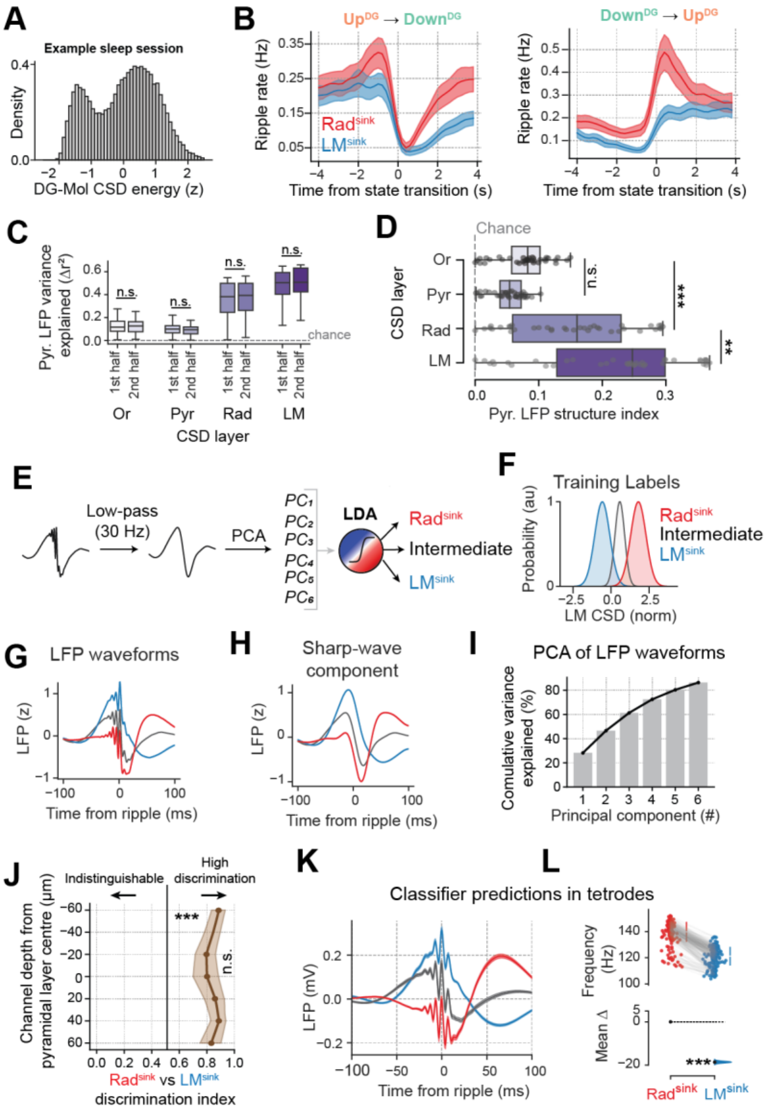

Figure S3: Occurrence of Rad^{sink} and LM^{sink} ripples in Up^{DG} and Down^{DG} states and relationship with pyramidal LFP waveforms, related to Figures 2, 3, and 4.

(A) Distribution of the CSD energy within the dentate gyrus molecular layer (DG-Mol) from an example sleep session.

(B) Triggered average Rad^{sink} and LM^{sink} ripple rate at the state transition times from Up^{DG} to Down^{DG} (left) and from Down^{DG} to Up^{DG} (right). Shaded areas, 95% CI (n = 23 sleep/rest sessions from five mice).

(C) Boxplots showing the variance in pyramidal layer ripple LFP explained by the CSD in each CA1 layer, separately for the first and second half of detected ripples within each session. This replicates the analysis from Figure 3B, here split into equally sized groups of earlier and later ripples. No significant differences were observed between the two sets for any layer, indicating that the results in Figure 3B are consistent throughout sleep (all Ps > 0.15; paired bootstrap test; n = 28 sleep/rest sessions from five mice).

(D) Pyramidal LFP Structure Index [1]. Boxplot showing the structure index (SI) of LFP ripple waveforms in CA1 pyramidal layer explained by the CSD values across CA1 layers. SI normalized by subtracting the average SI by chance (using surrogate models; see Methods). Note that currents in lacunosum-moleculare captured significantly more LFP variance than

other layers ($p = 2.7x10^{-17}$, one-way ANOVA; SI in LM CSD versus Rad CSD: $p = 2.4x10^{-3}$, Tukey post hoc; n = 38 sleep/rest sessions from 5 mice). Each dot represents a sleep session. Vertical gray dashed line indicates chance level.
(E-I) Rad^{sink} and LM^{sink} ripple profiles are predicted from the ripple waveform in *stratum pyramidale* LFPs. The schematic of the linear discriminant analysis (LDA) model used to classify Rad^{sink} and LM^{sink} ripples based on pre-processed LFP waveforms is shown in (E). Ripple events were divided into three classes (Rad^{sink}, Intermediate, and LM^{sink} ripples) based on digitised current values in the lacunosum-moleculare (F), the layer with the highest predictive power (see Figures 3B and S3C,D; and Methods). In (F), the red distribution represents Rad^{sink} ripples, the gray distribution represents Intermediate ripples, and the blue distribution represents LM^{sink} ripples. For each ripple, the pyramidal LFP waveforms were z-scored and low-pass filtered to isolate their 'sharp-wave' component (E, see method section 'Single-ripple CSD profile prediction from pyramidal LFP waveform'). The triggered average LFP waveforms and 'sharp-wave' components for the three ripple classes, as defined by the LM CSD values, are shown in (G) and (H). The filtered waveforms were then decomposed into six principal components (PCs), which collectively explained more than 80% of the variance across all ripples (I). These six PCs were used to train the LDA classifier (E) to predict the class of each ripple (i.e., Rad^{sink}, Intermediate, or LM^{sink}).
(J) Accuracy of the LDA model (from E; i.e., trained at the centre of the pyramidal layer) in discriminating Rad^{sink} and LM^{sink} ripples using pyramidal layer waveforms recorded from silicon probe channels located at ± 60 µm from the layer centre (see Table S1). Ripple types could be consistently discriminated along the radial axis, as shown by classification accuracy being above chance level (0.5) across depths. No systematic trend in accuracy was observed across recording depths (p = 0.45; one-way ANOVA; n = 14 recording days from five mice). *Shaded area*, 95%CI.
(K) Applying the silicon probe-validated CSD ripple-type classifier to the tetrode-recorded dataset retrieved LFP traces (referenced to the highest ripple-peak) for Rad^{sink}, intermediate, and LM^{sink} ripples that are consistent with those observed in the silicon probe-recorded dataset (Figure 1D).
(L) Estimation plot showing the effect size for the difference in the ripple frequency of events classified as Rad^{sink} and LM^{sink} by the ripple-type classifier in the tetrode-dataset (see Figure S3F-J). These match the ground-truth differences observed in the silicon probe dataset [Figure 1E; mean frequency (95% CI): Rad^{sink}, 139.1 (138.9 – 140.9) Hz; LM^{sink}, 122.1 (120.1 – 122.1) Hz; $p < 10^{-5}$; paired bootstrap test]. Each dot is a sleep session.
** p < 0.01, *** p < 0.001. (K and L) data shown are from n = 244 sleep/rest sessions from 13 mice implanted with tetrodes.

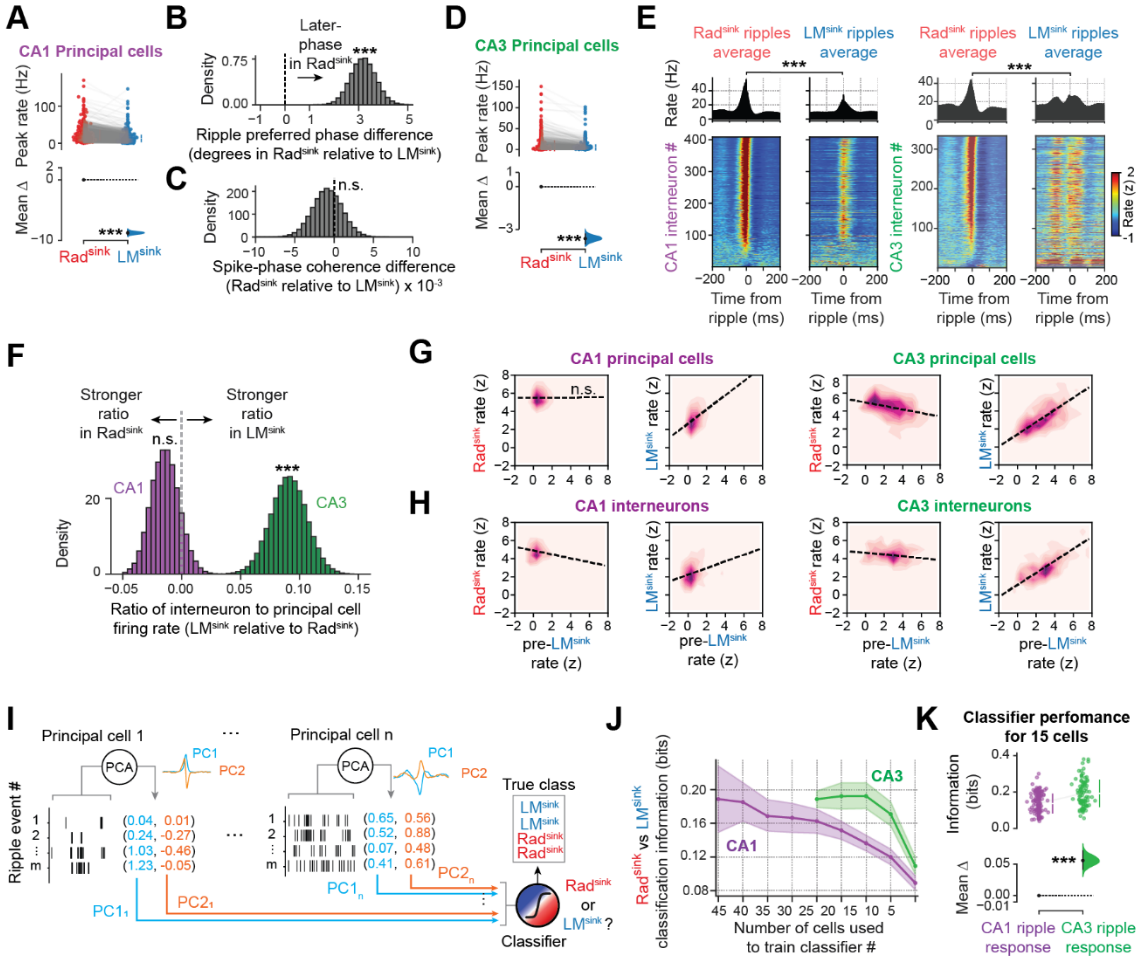

Figure S4: Differential CA1 and CA3 neuron responses in Rad^{sink} versus LM^{sink} ripples, related to Figure 4.

(A) Estimation plot showing the effect size for the difference in the peak firing rate of CA1 principal cells during Rad^{sink} versus LM^{sink} ripples. Each point represents a CA1 principal cell.

(B-C) Bootstrapped mean difference between Rad^{sink} and LM^{sink} ripples (relative to LM^{sink}) for ripple preferred firing phase (B) and spike-phase coherence (C) of CA1 principal cells. Overall, CA1 principal cells fired at earlier phases during LM^{sink} ripples ($p < 10^{-5}$; paired bootstrap test), while keeping similar phase coherence across the two ripple types ($p = 0.65$; paired bootstrap test; n = 2,196 CA1 principal cells).

(D) Same as (A), but for CA3 principal cells.

(E) Triggered average responses of interneurons in CA1 (left) and CA3 (right). *Top panels*, population average response in Rad^{sink} and LM^{sink} ripples. *Bottom panels*, firing response (z-scored) of individual interneurons. Cells sorted based on their firing rates in Rad^{sink} ripples. CA1 interneurons exhibited stronger responses in Rad^{sink} compared to LM^{sink} ripples (mean peak rate (95% CI): Rad^{sink}, 53.04 (48.36 – 57.85) Hz; LM^{sink}, 31.57 (28.55 – 34.70) Hz; $p < 10^{-5}$, paired bootstrap test; n = 408 interneurons). Similarly, CA3 interneurons showed heightened activity in Rad^{sink} ripples (mean peak rate (95% CI): Rad^{sink}, 41.48 (37.02 – 46.08) Hz; LM^{sink}, 27.57 (24.65 – 30.57) Hz; $p < 10^{-5}$, paired bootstrap test; n = 333 interneurons).

(F) Bootstrapped mean difference in the interneuron to principal cell firing ratio between LM^{sink} and Rad^{sink} ripples. Note that while this ratio is similar between Rad^{sink} and LM^{sink} ripples in CA1 ($p = 0.48$; paired bootstrap test), CA3 exhibits a significantly higher ratio (i.e.,

higher interneuron firing relative to principal cells) in LM^{sink} ripples compared to Rad^{sink} ripples ($p < 10^{-5}$; paired bootstrap test).

(G,H) Contour plots showing the 2D histogram of mean firing rates during Rad^{sink} and LM^{sink} ripples (y-axes) versus ~100 ms before LM^{sink} ripples (pre-LM^{sink}; x-axis), for each group of cells [CA1 and CA3 principal cells (G), or interneurons (H)]. Firing rates were z-scored relative to Rad^{sink} ripple rates, as in Figures 4B and E. Firing rates during LM^{sink} ripples were significantly correlated with pre-LM^{sink} rates (all $Ps < 10^{-5}$). In contrast, pre-LM^{sink} rates were significantly anti-correlated with Rad^{sink} ripple rates for all groups except CA1 principal cells ($p = 0.94$; all other $Ps < 10^{-3}$).

(I-K) Classification accuracy of Rad^{sink} and LM^{sink} ripples in CA1 and CA3. Shown in (I) is a schematic of the method used to classify Rad^{sink} versus LM^{sink} ripples based on the entire spike train around ripples (see section 'Population activity discrimination of Rad^{sink} versus LM^{sink} ripples'). The first two principal components (PCs) of these time responses were computed for each cell independently. PCs from all cells were then concatenated and used to train a logistic regression classifier to predict whether a given ripple was Rad^{sink} or LM^{sink}. Shown in (J) is the classifier accuracy, as a function of the number of cells used for the training stage, separately for CA1 and CA3. Shown in (K) is the corresponding estimation plot representing the effect size for the difference in the accuracy of the CA1 versus CA3 classifiers (trained on 15 principal cells). CA3 discriminated Rad^{sink} versus LM^{sink} significantly better than CA1; $p < 10^{-5}$; bootstrap test; n = 129 sleep sessions for CA1 and n = 89 for CA3; see also Table S2). This finding aligns with the observation that CA3 principal cells exhibit greater differences in response between Rad^{sink} and LM^{sink} ripples compared to CA1 (Figure 4).

*$p < 0.05$, **$p < 0.01$, ***$p < 0.001$.

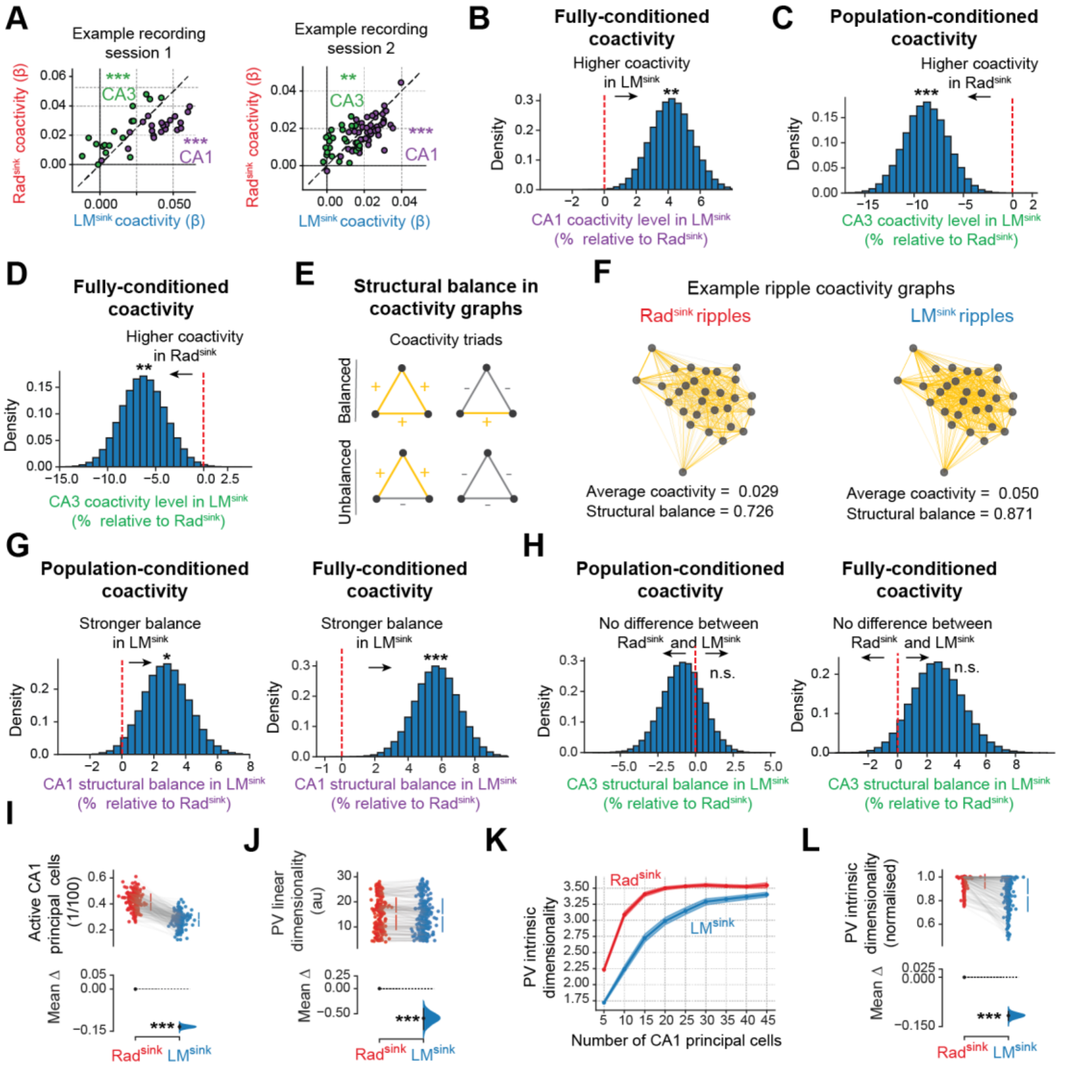

Figure S5: Coactivity and CA1 population vectors analyses between Rad^{sink} and LM^{sink} ripples, related to Figure 5.

(A) Scatter plots showing the mean population-conditioned coactivity of CA1 (purple) and CA3 (green) principal cells during LM^{sink} versus Rad^{sink} ripples from two example sleep sessions. Population-conditioned coactivity measured the coactivity between two principal cells while regressing out the population rate (see Figure 5A and Methods section 'Neuronal coactivity graphs'). Each point represents the average coactivity value of a CA1 or a CA3 principal cell. The dashed black line indicates the diagonal of the first and third quadrants. Note that CA1 principal cells exhibit higher coactivity during LM^{sink} ripples, whereas CA3 principal cells show higher coactivity during Rad^{sink} ripples (all Ps < 0.01; bootstrap tests; sleep session 1: n = 21 CA1 and n = 17 CA3 principal cells; sleep session 2: n = 48 CA1 and n = 25 CA3 principal cells).

(B) Bootstrapped mean difference in fully-conditioned coactivity CA1 principal cells (computed as LM^{sink} minus Rad^{sink} ripples), showing that cells were more coactive during LMs^{ink} than Rad^{sink} ripples ($p = 1.02 \times 10^{-3}$; paired bootstrap test). Difference expressed as a percentage relative to the mean coactivity in Rad^{sink} ripples. Fully-conditioned coactivity was measured by predicting one cell from all other neurons (see Methods section 'Neuronal coactivity graphs').

(C) Bootstrapped mean differences in population-conditioned coactivity of CA3 principal cells (LM^{sink} minus Rad^{sink}). These results indicate, as highlighted by the black arrow, that CA3 principal cells are more coactive during Rad^{sink} ripples than LM^{sink} ripples, contrasting with the pattern observed for CA1 cells (Figure 5B; $p = 2x10^{-5}$; paired bootstrap test; n = 866 CA3 principal cells from 11 mice).
(D) Same as (C) but for CA3 fully-conditioned coactivity ($p = 7.7x10^{-3}$; paired bootstrap test).
(E) Schematics of balanced and unbalanced triads of coactivity used to assess structural balance in ripple-nested neuronal graphs.
(F) Example neuronal graphs representing the ripple adjacency matrices shown in Figure 5A, including its average coactivity and structural balance.
(G) Bootstrapped mean difference in structural balance for CA1 ripple population-conditioned (left) and fully conditioned (right) coactivity graphs (Rad^{sink} minus LM^{sink}). Difference expressed as a percentage relative to the mean coactivity in Rad^{sink} ripples. These results show that LM^{sink} coactivity graphs have higher structural balance than Rad^{sink} graphs (all Ps < 0.05; paired bootstrap tests).
(H) Same as (G) but for CA3 principal cells coactivity graphs. There was no significant difference in structural balance between the two ripple types for CA3 coactivity graphs, unlike for CA1 graphs (all Ps > 0.10; paired bootstrap test; n = 171 sleep/rest sessions from 11 mice).
(I) Estimation plot showing the effect size for the difference in the proportion of active CA1 principal cells (i.e., cells that fire at least one action potential) between the two ripple profiles. Rad^{sink} ripples recruit a higher proportion of CA1 principal cells compared to LM^{sink} ripples. Each dot represents a single sleep session.
(J) Estimation plot showing the effect size for the difference in the linear dimensionality of population vectors CA1 principal in Rad^{sink} versus LM^{sink} ripples. Each dot represents a single sleep session.
(K) Intrinsic dimensionality as a function of the number of CA1 principal cells used to compute it. *Shaded areas*, 95%CI.
(L) Estimation plot showing the effect size for the difference in the chance-normalised intrinsic dimensionality of population vectors CA1 principal in Rad^{sink} versus LM^{sink} ripples. The dimensionality was normalised relative to the dimensionality of higher-dimensional surrogate population vectors, which preserved the difference in sparsity across the two ripple types but destroyed the coactivity structure. After accounting for the sparsity difference with these surrogate dimensionalities, Rad^{sink} were still higher dimensional than LM^{sink} ripples ($p < 10^{-5}$; paired bootstrap test). Each dot represents a single sleep session.
*** p < 0.001. (B,G,I-L) data shown are from n = 208 sleep/rest sessions from 13 mice.

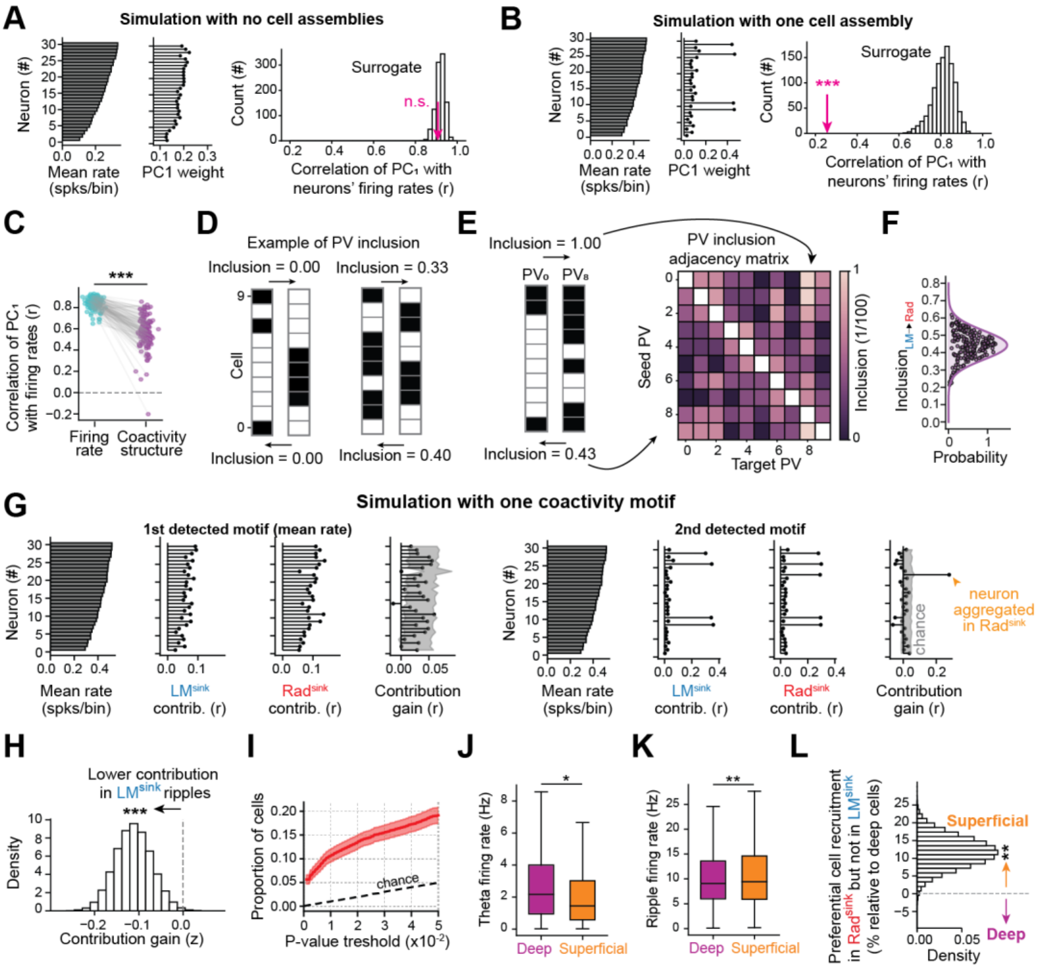

Figure S6: Core LM^{sink} versus composite Rad^{sink} ripples and CA1 deep and superficial recruitment, related to Figures 5 and 6.

(A-C) Accounting for individual firing rates in motif structure. Principal Component Analysis (PCA) framework to isolate the contribution of the firing rates alone in the expression of the coactivity motif structure.

(A) Simulation of neural data in which ripple recruitment is fully explained by neuronal excitability. We simulated a dataset of 30 neurons with varying excitability (defined as each neuron's probability of being active during a ripple), combined with population-level rate fluctuations (i.e., shared modulation across all neurons). Importantly, aside from the shared modulation, neurons were simulated independently of one another. In this setting, PC1 weights (centre panel) were highly correlated with individual firing rates (right panel), indicating the absence of structured coactivity. This correlation did not differ significantly from that observed in surrogate matrices that preserved individual firing rates and ripple-wise population rates. These results confirm that the observed motifs were fully explained by excitability, reflecting the absence of structured coactivity.

(B) Same as (A), but for a second simulation in which we introduced a coactivation motif shared by a subset of neurons, generating structured activity decoupled from the overall population rate. In this case, the correlation between PC1 weights and individual firing rates was markedly reduced and significantly lower than in surrogate datasets, indicating that PC1 now primarily captured structured coactivity beyond what could be explained by excitability alone.

(C) Correlation between the firing rates of tetrode-recorded CA1 principal cells and their PC1 weights derived from LM^{sink} ripples (purple). These values are compared to

correlations obtained from surrogate datasets that preserved individual firing rates but destroyed coactivity structure (cyan). Correlations in the real data were significantly lower than in the surrogates, indicating that excitability alone does not account for the observed motif structure (n = 136 sleep sessions from 10 mice).

(D-F) Inclusion analysis. Shown in (D) is a schematic illustrating the procedure (asymmetric Jaccard similarity) for calculating the inclusion of the set of active cells across two population vectors (PVs; one as the seed vector; the other as the target vector). Each active cell in each PV is depicted by a black field. Pairwise inclusion values are computed for each pair of PVs (m, n). Shown in (E) is an example adjacency matrix containing these inclusion values. Shown in (F) is the distribution of the average inclusion values of motifs of active CA1 principal cells in $LM \rightarrow Rad$ ripples [mean inclusion (95%CI): 0.44 (0.43-0.45)]. Each dot represents a sleep session (n = 208 sleep/rest sessions from 13 mice).

(G-I) Rad^{sink} ripples aggregation of neurons into LM^{sink}-defined core motifs.

(G) Simulation of neurons integrating into LM^{sink} motifs during Rad^{sink} ripples. We simulated data from 30 neurons with varying excitability, modulated by population-level rate fluctuations, alongside an embedded motif (i.e. an assembly) shared by a subset of neurons. To mimic the empirical differences in ripple power and sparsity between Rad^{sink} and LM^{sink} ripples, LM^{sink} events were simulated with lower population rates and a sparse motif. In Rad^{sink} events, the same motif was preserved, but an additional neuron was recruited, and overall population rates were higher. Motif detection in LM^{sink} ripples yielded two coactivity patterns: the first captured population-wide activation related to excitability (1st detected motif), and the second reflected the structured coactivation among the subset of neurons (2nd detected motif). For each detected motif, we plotted: (1) the mean firing rate of the neurons (left); (2) each neuron's contribution to the assembly during LM^{sink} (centre-left) and Rad^{sink} (centre-right) ripples; and (3) the contribution gain from LM^{sink} to Rad^{sink} , relative to chance (right), where chance was computed from surrogate data controlling for excitability. As expected, the population-rate-related motif (left) showed no significant gain in contribution beyond chance, indicating it was fully explained by excitability. In contrast, for the structured motif (right), the method successfully identified one neuron whose contribution significantly increased during Rad^{sink} ripples, consistent with aggregation into the LM^{sink} motif.

(H) Bootstrapped change in mean cell contribution (z-scored relative to chance) from Rad^{sink} to LM^{sink} ripples. For each detected motif, the contribution change was computed as the mean across all cells. Note that the mean of this change is significantly negative, indicating that cells tend to be integrated into LM^{sink} motifs during Rad^{sink} ripples (and not the other way around) more than expected based on their excitability ($p = 7.4 \times 10^{-3}$; bootstrap test; n = 231 motifs detected in 10 mice).

(I) Proportion of cells within each detected LM^{sink} motif that significantly increased their contribution during Rad^{sink} ripples, relative to their excitability. The y-axis shows the observed proportion of cells exceeding a given p-value threshold (x-axis), based on surrogate distributions controlling for each cell's excitability. The dashed diagonal indicates the false discovery rate under the null hypothesis. Since the observed proportion lies above this diagonal suggests that more cells than expected by chance were aggregated into LM^{sink} motifs during Rad^{sink} ripples. *Shaded area*, 95% CI (n = 136 sleep sessions from 10 mice).

(J-L) Differential firing properties of CA1 deep and superficial cells and recruitment in Rad^{sink} and LM^{sink} ripples.

(J,K) Boxplots showing the mean firing rates of classified CA1 deep and superficial cells during theta cycles (J) and ripple events (K). Deep cells fired at higher rates than superficial cells during theta (p = 0.017; bootstrap test) whereas superficial cells fired more strongly during ripples ($p = 8 \times 10^{-3}$; bootstrap test).

(L) Preferential cell recruitment in Rad^{sink} ripples. We computed the conditional probability of cells to be selectively active in Rad^{sink} ripples (given their inactivity in LM^{sink} ripples), normalised relative to chance, and shown as a percentage relative to the mean of deep cells. Superficial cells were more likely than deep cells to be active in Rad^{sink} and not in LM^{sink} ripples ($p = 2.7x10^{-3}$; bootstrap test; n = 1,100 deep and 480 superficial cells). *Dashed line*, chance level.

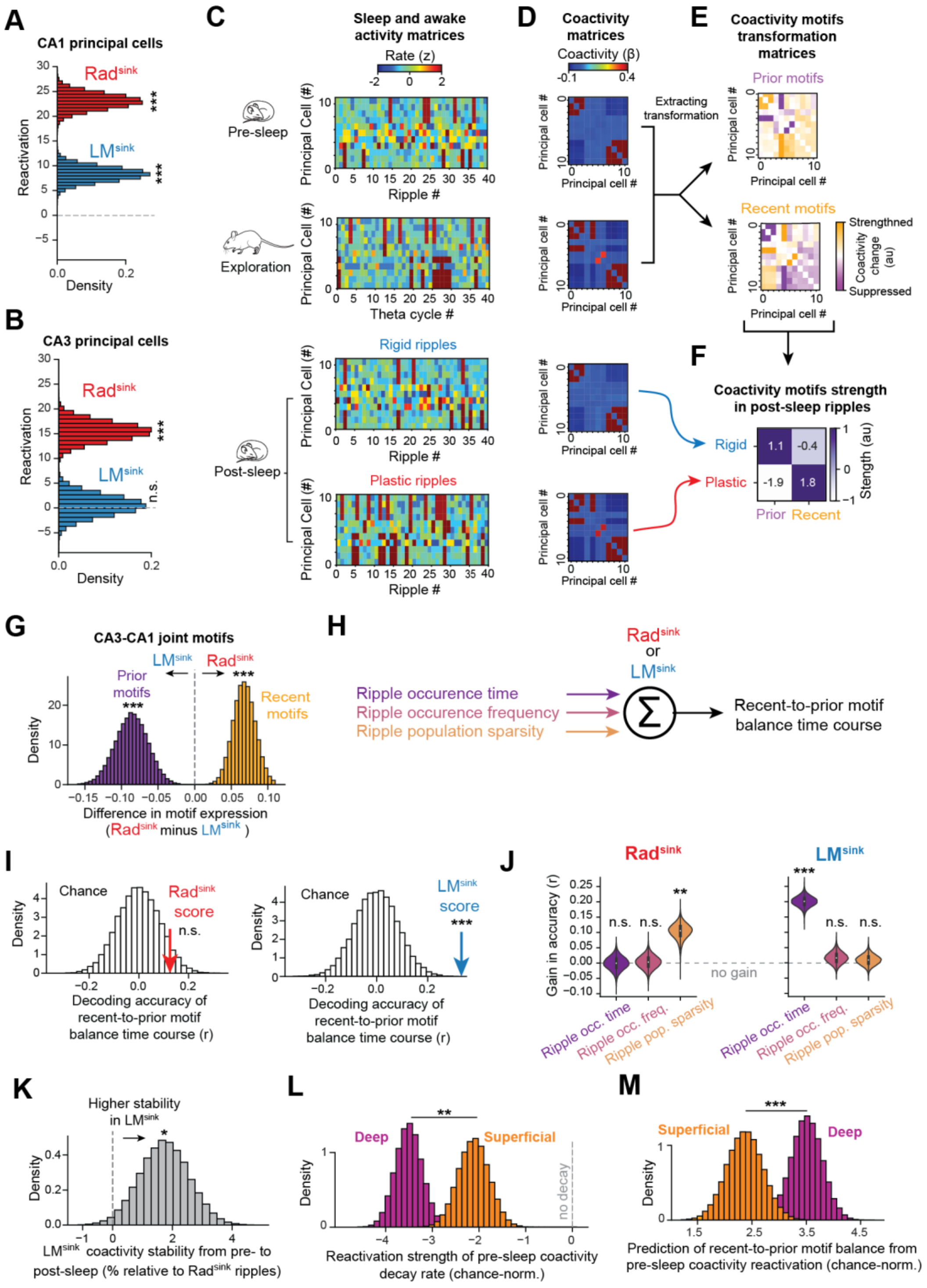

Figure S7: Reactivation of CA1 versus CA3 neurons and prior versus recent coactivity motifs, related to Figures 6 and 7.

(A,B) Offline reactivation of waking theta patterns in CA1 and CA3 principal cells. Shown in (A) is the bootstrapped mean difference in the reactivation of CA1 principal cells during Rad^{sink} and LM^{sink} ripples, expressed as a percentage relative to pre-sleep (all Ps < 10^{-5}; 1-tailed bootstrap tests). The same is shown in (B) but for CA3 principal cells, which reactivate in Rad^{sink} ripples ($p < 10^{-5}$; 1-tailed bootstrap test) but not in LM^{sink} ripples ($p = 0.39$; 1-tailed bootstrap test). Data shown are from n = 1,580 CA1 and 866 CA3 principal cells.

(C-F) Extraction and analysis of prior and recent coactivity motifs in simulated neural activity. We validated the method described in the section "Recent-to-prior coactivity motif balance" using a simulation. We first simulated a pre-sleep population activity matrix containing two pre-defined motifs of coactive neurons ("prior motifs"). Subsequently (i.e., during awake exploration), one of these prior motifs underwent re-organisation by gaining an additional neuron, while a new (third) motif of coactive neurons emerged ("recent motif"). We then simulated two post-exploration sleep population activity matrices: one only expressed the motifs previously observed in pre-exploration sleep ("rigid ripples"), and the other incorporated the exploration-related motif ("plastic ripples"). Shown are example activity matrices over time bins for each session (C), with the corresponding coactivity matrices (D). We next extracted two transformation matrices from the pre-exploration sleep and the exploration coactivity matrices: the prior coactivity motifs (present in pre-sleep) and the recent motif (emerging during exploration and absent in pre-sleep) (E). These transformation matrices featured the cell pairs composing these motifs, highlighted in orange to denote strengthened connections (top panels for prior motifs and bottom panels for recent motifs). These transformation matrices were then applied to the simulated post-exploration sleep activity to evaluate the strength of each motif (F). The heatmap in (F) summarizes the strength of prior and recent coactivity motifs in the two post-exploration sleep cases from (C,D), demonstrating that rigid ripples more strongly expressed prior motifs, while plastic ripples predominantly reactivated recently-expressed coactivity motifs.
(G) Bootstrapped mean difference in CA3-CA1 motif expression during post-sleep for prior and recent motifs. The difference was computed as Rad^{sink} minus LM^{sink} ripples, thus reflecting the relative strength of each motif type across the two ripple types. In line with Figure 7B, CA3-CA1 motifs during Rad^{sink} ripples better aligned with recently expressed theta coactivity motifs, whereas LM^{sink} ripples preferentially expressed prior motifs (all Ps <= $2x10^{-5}$; paired bootstrap tests; n = 111 pairs of pre- and post-sleep sessions from 11 mice).
(H-J) Factors explaining temporal changes in recent-to-prior motif balance over hour-long sleep (see Figure 7D). We trained linear regression models to predict the recent-to-prior motif balance over the hour-long sleep/rest (H). These models were trained independently for Rad^{sink} and LM^{sink} ripples. Three features were used: ripple occurrence (occ.) time, ripple occurrence frequency (freq.), and ripple population sparsity as predictors (in 10-minute bins). Shown in (I) is the accuracy of each model alongside its corresponding chance level (*left*, Rad^{sink}; *right*, LM^{sink}). Chance level estimated by shuffling recent-to-prior balance scores 25,000 times. Red/Blue arrow indicates the observed model accuracy. Note that the Rad^{sink} model did not significantly predict the recent-to-prior motif balance time course (p = 0.068; 1-tailed bootstrap test), consistent with the stable trend observed throughout sleep/rest (Figure 7D). In contrast, changes in recent-to-prior motif balance over time during LM^{sink} ripples were significantly predicted ($p < 4x10^{-5}$; 1-tailed bootstrap test). Shown in (J) are violin plots showing the contribution of each predictor to models' accuracy. *Left*, for the Rad^{sink} model, only ripple population sparsity significantly contributed ($p = 2.4x10^{-3}$, 1-tailed bootstrap test, Bonferroni corrected). *Right*, for the LM^{sink} model, only ripple occurrence time significantly contributed ($p < 4x10^{-5}$, 1-tailed bootstrap test, Bonferroni corrected).
(K) Bootstrapped mean difference in the stability of CA1 principal cell coactivity from pre-exploration to post-exploration sleep in Rad^{sink} and LM^{sink} ripples (expressed as a percentage relative to Rad^{sink} ripples). LM^{sink} motifs are more stable than Rad^{sink}, being more consistent across the two sleep sessions either side of exploration [mean stability in LM^{sink} ripples relative to Rad^{sink} ripples (95% CI): 1.74 (0.12 – 3.35) %; p = 0.018; 1-tailed paired bootstrap test; n = 140 pre- and post-sleep session pairs].
(L) Decay of the reactivation of pre-sleep coactivity across post-sleep in LM^{sink} ripples for deep and superficial CA1 cells. Reactivation strength was computed in non-overlapping 10-

minute windows, separately for deep and superficial cells, and regressed against the logarithm of post-sleep time. The slope of this regression reflects the decay rate of prior motif expression. For each session, a chance distribution was generated by circularly shuffling the temporal order of time bins. The mean of this null distribution was subtracted from the observed slope, yielding a chance-normalized decay rate. Histograms show the bootstrapped mean estimates of these slopes for deep and superficial CA1 principal cells. Both populations showed significantly negative slopes (all Ps < 10^{-5}; bootstrap test), indicating a decline in reactivation strength over time. However, this decay was significantly steeper in deep cells ($p = 1.4 \times 10^{-3}$; bootstrap test).

(M) The drift in recent-to-prior motif balance is better explained by reactivation of pre-exploration sleep coactivity in deep cells than in superficial cells. Pre-sleep reactivation strength and recent-to-prior balance were computed in non-overlapping 10-minute bins, separately for deep and superficial CA1 cells, as in (L). For each session, we calculated the correlation between the time courses. A chance distribution was generated by circularly shuffling the temporal order of one the trace, and the mean of this null distribution was subtracted from the observed correlation to yield a chance-normalized value. Histograms show the bootstrapped mean of these normalized correlations for each population. Deep cell reactivation time courses accounted for significantly more variance in motif balance dynamics than superficial cell reactivation ($p = 9.4 \times 10^{-3}$; bootstrap test).

*p < 0.05, **p < 0.01, ***p < 0.001. (L-M) data shown are from n = 715 deep and n= 378 superficial cells from nine mice.

Strain	RRID	n	Implant
C57Bl6/J	IMSR_JAX:000664	1	NeuroNexus A1x32-5mm-25-177-H32_21mm
C57Bl6/J	IMSR_JAX:000664	1	NeuroNexus A1x64-edge-6mm-20-177-H64LP_30mm
C57Bl6/J	IMSR_JAX:000664	3	Cambridge NeuroTech ASSY-236 H3 Chronic 64-Molex

Supplementary Table S1. Silicon probe details, related to Figures 1 and 2.

Classifier input	Ripple-peak population vectors	Entire temporal ripple response
CA1 principal cells	9.25 (8.54 – 9.99)	13.64 (12.70 – 15.59)
CA3 principal cells	6.17 (4.81 – 7.62)	19.23 (17.70 – 20.81)

Supplementary Table S2. Performances of models discriminating Rad^{sink} versus LM^{sink}, related to Figure 4.
Shown are the cross-validated performance [mean (95% CI); bits; x 10^{-2}] of classifiers trained to predict Rad^{sink} versus LM^{sink} ripple types based on CA1 or CA3 activity, using either their population vectors (15 principal cells each) at the ripple peak or the entire temporal spiking profile around the ripple. Performance quantified as the mutual information (M.I.) between the true and predicted ripple types.

Supplemental references list

1. Sebastian, E.R., Esparza, J., and Prida, L.M. de la. (2024). Quantifying the distribution of feature values over data represented in arbitrary dimensional spaces. PLOS Computational Biology 20, e1011768.
